# Advances in Metal Halide Perovskite Scintillators for X-Ray Detection

**DOI:** 10.1007/s40820-025-01772-7

**Published:** 2025-05-23

**Authors:** Ting Wang, Guoqiang Zeng, Yang Michael Yang, Zhi Yang, Tianchi Wang, Hao Li, Lulu Han, Xue Yu, Xuhui Xu, Xiaoping Ouyang

**Affiliations:** 1 College of Materials and Chemistry & Chemical Engineering, Nuclear Technology Key Laboratory of Earth Science, Chengdu University of Technology, Chengdu, 610059 People’s Republic of China; 2 State Key Laboratory of Intense Pulsed Radiation Simulation and Effect, Northwest Institute of Nuclear Technology, Xi’an, 710024 People’s Republic of China; 3 Nuclear Technology Key Laboratory of Earth Science, Chengdu University of Technology, Chengdu, 610059 People’s Republic of China; 4 Faculty of Materials Science and Engineering, Key Laboratory of Advanced Materials of Yunnan Province, Kunming University of Science and Technology, Kunming, 650093 People’s Republic of China; 5 Key Laboratory of Materials Physics of Ministry of Education, Laboratory of Zhongyuan Light, School of Physics, Zhengzhou University, Zhengzhou, 450051 People’s Republic of China; 6 State Key Laboratory of Modern Optical Instrumentation, Institute for Advanced Photonics, College of Optical Science and Engineering, Zhejiang University, Hangzhou, 310027 People’s Republic of China; 7 School of Mechanical Engineering, Institute for Advanced Materials, Chengdu University, Chengdu, 610106 People’s Republic of China

**Keywords:** Metal halide perovskites, X-ray detection, Scintillators, Radioluminescence properties, Engineering strategies

## Abstract

The relentless pursuit of advanced X-ray detection technologies has been significantly bolstered by the emergence of metal halides perovskites (MHPs) and their derivatives, which possess remarkable light yield and X-ray sensitivity. This comprehensive review delves into cutting-edge approaches for optimizing MHP scintillators performances by enhancing intrinsic physical properties and employing engineering radioluminescent (RL) light strategies, underscoring their potential for developing materials with superior high-resolution X-ray detection and imaging capabilities. We initially explore into recent research focused on strategies to effectively engineer the intrinsic physical properties of MHP scintillators, including light yield and response times. Additionally, we explore innovative engineering strategies involving stacked structures, waveguide effects, chiral circularly polarized luminescence, increased transparency, and the fabrication of flexile MHP scintillators, all of which effectively manage the RL light to achieve high-resolution and high-contrast X-ray imaging. Finally, we provide a roadmap for advancing next-generation MHP scintillators, highlighting their transformative potential in high-performance X-ray detection systems.

## Highlights

- The review highlights recent advancements in enhancing the intrinsic physical properties of metal halide perovskite scintillators, such as improving light yield and response times, to improve their X-ray detection performance.
- It discusses innovative engineering strategies to effectively optimize radioluminescent light management for high-resolution X-ray imaging.
- The problems encountered in the application of metal halides perovskites materials for X-ray detection are summarized, and the potential development direction in the future is prospected.

## Graphical abstract

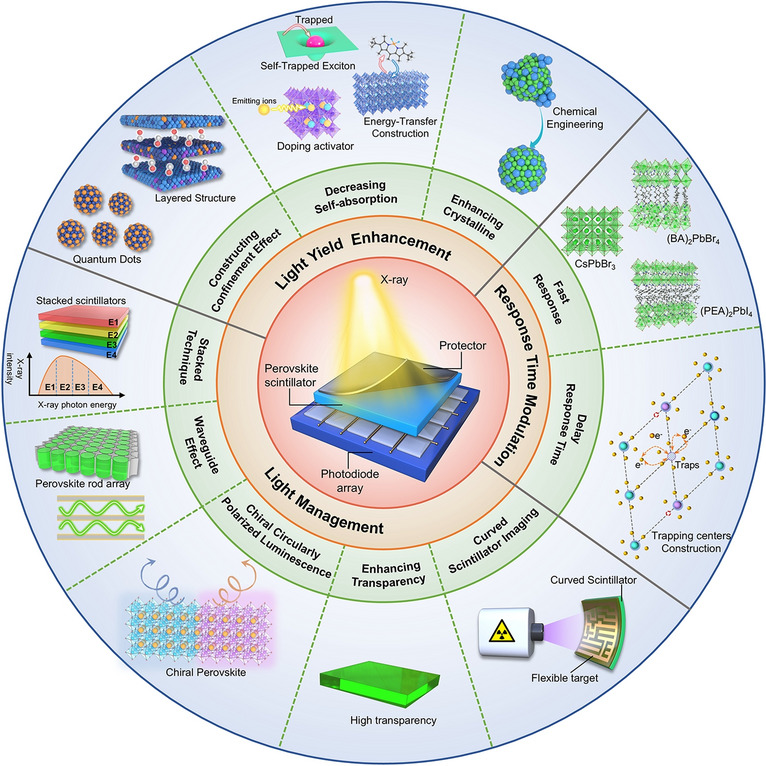

## Introduction

The imperative for sophisticated X-ray detection technologies has never been more critical, with applications spanning the realms of medical imaging, security surveillance, scientific research, and industrial non-destructive testing [1, 2] Based on their detection mechanisms, X-ray detectors are broadly categorized into direct and indirect types. Direct detectors use semiconductors to immediately convert X-rays into electrical signals, whereas indirect systems employ scintillators to first transform X-rays into visible light, then detected by photodetectors [3]. Despite faster response times in direct detection, indirect detectors dominate commercial applications use due to their higher efficiency, better stability, and lower costs [4–7]. In the realm of scintillators, however, traditional scintillators each have limitations that hinder their effectiveness across the practical applications. For instance, columnar CsI: Tl is commonly utilized in medical radiography for its high light yield and good resolution [8, 9], but its hygroscopic nature can degrade performance over time. Ceramic Gd_2_O_2_S (GOS), used in X-ray computed tomography (CT), offers high light output and stability [10–13], but has a relatively long decay time and can be costly to produce, limiting its use in cost-sensitive applications. Similarly, single crystalline Lu_1.8_Y_0.2_SiO_5_:Ce (LYSO: Ce) is favored in positron emission tomography (PET) for its high light yield and fast decay time [14, 15], yet it is expensive and challenging to grow in large, defect-free crystals, affecting its widespread adoption. These limitations underscore the need for ongoing research and development to create new scintillator materials that better meet the diverse demands of various imaging applications. The evolving demand for detecting intricate structures, high-precision equipment, irregular geometries, and efficiently storing imaging information necessitates the development of scintillators with exceptional characteristics. Therefore, developing novel scintillators with high light yield, high resolution, tunable response time, and adaptability is essential for pushing the boundaries of X-ray detection technology and for broadening their application scope.

Metal halide perovskites (MHP), with their exceptional optical properties and unparalleled X-ray detection capabilities, have risen to prominence as the forefront of scintillation materials. This is attributed to their high effective atomic number (*Z*_eff_) and near-unity luminescence efficiency [16, 17], which can be demonstrated by comparing the scintillation properties of commonly used materials and the recently reported MHPs (Table 1). The journey of perovskite materials in X-ray applications began in 2002 with the introduction of the hybrid organic–inorganic perovskite (C_6_H_13_NH_3_)_2_PbI_4_, by Shibuya et al. [18]. Fast forward to 2016, Birowosuto et al. [19] discovered a MHP material with an unprecedented light yield of up to 120,000 Ph MeV^−1^, setting a new benchmark for efficiency in scintillation-based radiation detection. Subsequently, a series of studies have been reported on optimizing the light yield, timing, and detected resolution of MHP scintillators [20–23]. The ability to tailor MHP properties through meticulous compositional adjustments and structural engineering has since opened up unprecedented opportunities for enhancing detection performance across a spectrum of modalities [24–27]. More importantly, researchers have been able to flexibly manipulate the bandgap, decay kinetics, and improve the stability of these materials, making them more viable for practical applications in medical imaging, security systems, and industrial inspection [28–30].

**Table 1:** RL peak, density, light yield, decay time, spatial resolution, and detection limit for commonly used scintillator materials

Scintillators	Emission peak (nm)	Density (g cm^−3^)	Light yield (Ph Mev^−1^)	Decay time (ns)	Spatial resolution (lp mm^−1^)	Detection limit (nGy_air_ s^−1^)	References
Bi_3_Ge_3_O_12_(BGO)	480	7.13	8,500	300	11	376	[31–33]
Lu_2_SiO_5_(LYSO)	420	7.4	29,000	40	3.8	1.33 × 10^4^	[33–35]
Gd_2_O_2_S: Eu	610	6.7	60,000	1 × 10^6^	–	–	[12]
CsI: TI	470	4.51	65,000	10^3^	10	180	[9, 31]
Y_3_Al_5_O_12_: Ce	550	4.57	46,400	70	–	–	[36]
CsPbBr_3_	520	4.55	26,000	5.97	12.5	120	[37, 38]
(BA)_2_PbBr_4_	427	4.05	7,900	2.52	–	318.3	[29, 39]
(BA)_2_(Pb_0.9_Mn_0.1_)Br_4_	618	4.05	85,000	7.28 × 10^5^	10.7	16	[40]
Cs_3_Cu_2_I_5_	445	5.6	127,376	9.6 × 10^2^	17	48.6	[39, 41]
CsCu_2_I_3_	570	5.01	36,000	110	15.6	88.9	[42, 43]
Rb_2_CuBr_3_	385	5.97	91,056	4.14 × 10^7^	27.9	63	[4, 40]
Cs_2_AgI_3_:Cu(I)	470	4.62	82,900	192.8	16.2	77.8	[33, 41]
(BPTP)_2_MnBr_4_	515	1.684	136,000	3.18 × 10^5^	10.1	282	[44]
(C_8_H_20_N)_2_Cu_2_Br_4_	468	1.944	91,300	5.6 × 10^4^	9.5	52.1	[45, 46]
(C_25_H_22_P)_2_MnBr_4_	512	1.586	78,937	3.3 × 10^5^	25.6	108.2	[4, 47]
Cs_2_ZrCl_6_	447	3.94	49,400	1.56 × 10^6^	18	65	[48, 49]
Cs_2_ZrCl_6_: Lu^3+^	460/90	3.94	94,190	9.0 × 10^4^	17.2	15.8	[23]

Numerous reviews have explored methods to enhance the performance of MHP scintillators for X-ray detection, focusing on aspects such as imaging resolution and detection limits [50]. These reviews often emphasize optimizing the light yield of the scintillators. However, improving intrinsic properties like light yield alone is not sufficient. The response time, another critical intrinsic property, plays a vital role in determining the effectiveness of X-ray detection, impacting both real-time and delayed detection capabilities. Additionally, the design and implementation of engineering techniques for effective radioluminescent (RL) management, encompassing the optimal collection and transmission of the light signals generated by the scintillators, are crucial for maximizing the detector's overall performance. Therefore, a comprehensive overview of the cutting-edge advancements and strategic approaches, integrating both the intrinsic physical properties and RL engineering techniques, is urgently needed to summarize current research progress and explore future prospects in this field.

This review begins with an elucidation of the MHP scintillation mechanism and the fundamental requirements of scintillators. It then summarizes recent research focused on strategies to effectively engineer the intrinsic physical properties of MHP scintillators, such as enhancing light yield and optimizing response times. These light yield enhanced strategies are summarized, which include optimizing crystallinity, constructing confinement effects to reduce non-radiative recombination pathways, suppressing self-absorption by exploiting self-trapped exciton (STE) emission, doping with emitting centers, and constructing an energy transfer (ET) channel. Additionally, the pivotal role of response time modulation in scintillators elucidates its impact on the dynamics of response and the overall effectiveness of detection systems, and details how the modulation of decay times can be tailored to meet the demands of various applications, from high-speed imaging to long-term radiation detection. Furthermore, the pursuit of high-resolution X-ray detection has driven the development of innovative engineering techniques, such as the construction of stacked imaging, waveguide effects, chiral circularly polarized luminescence (CPL), and the improvement in transparency. The emergence of flexible scintillators has further expanded the applicability of X-ray detection technologies, enabling conformal imaging on curved surfaces and in hard-to-reach areas. Ultimately, the review delineates the prevailing challenges and offers insightful perspectives for the future evolution and applications of MHP scintillators.

## Mechanism of MHP Scintillators

**Fig. 1:**
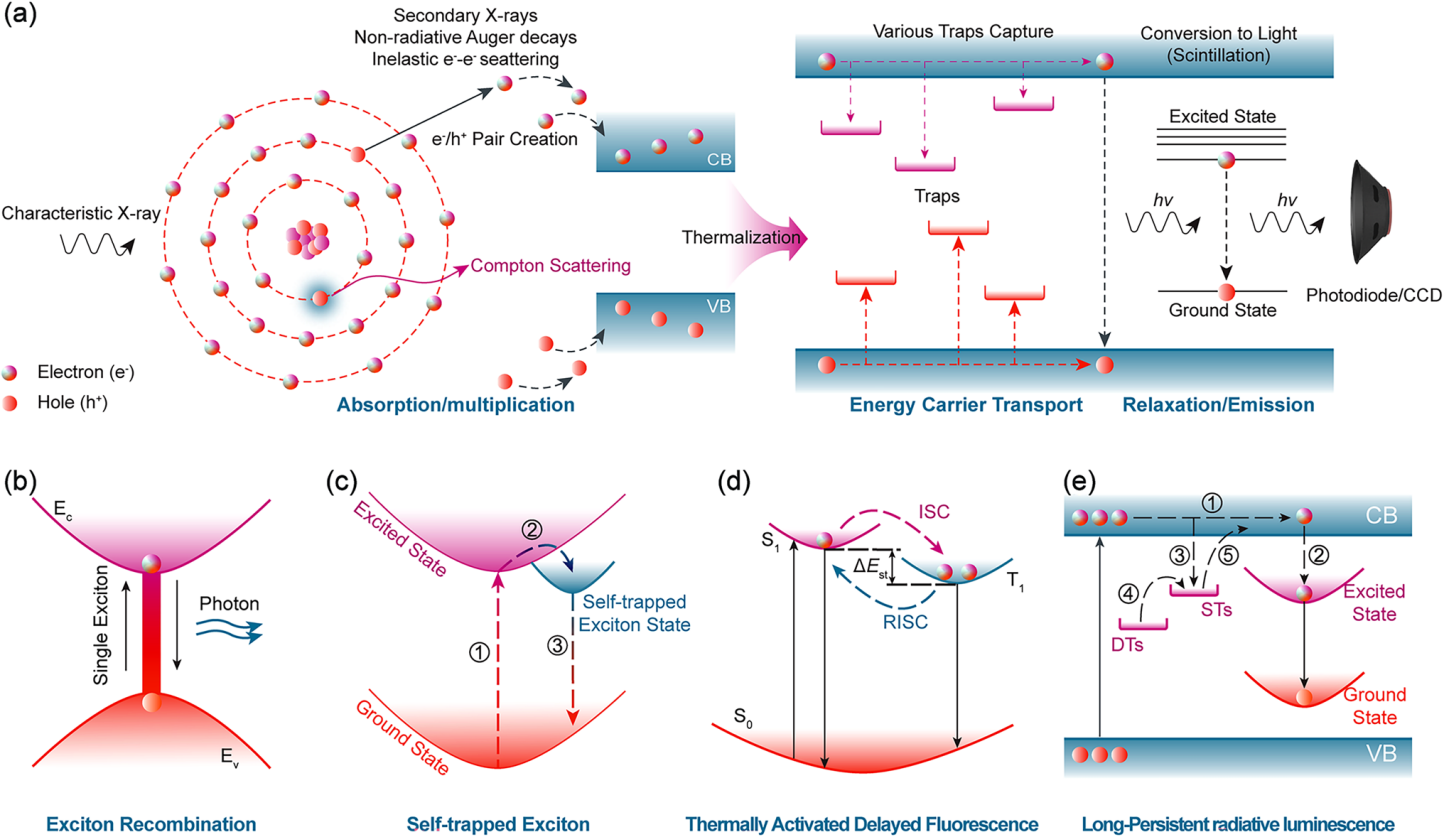
Mechanism of MHP Scintillators for X-ray Detection. **a** Three-stage scintillation processes: conversion of X-rays to UV/visible photons, transport of charge carriers, and luminescence via radiative recombination. **b** Exciton recombination mechanism: Excitons formed by electron–hole pairs migrate and emit photons upon recombination. **c** STE emission: Electrons and holes localize in a distorted lattice, forming a low-energy bound state that leads to longer lifetimes and broader emission. **d** TADF emission: Triplet excitons convert to singlet states via RISC, resulting in delayed radiative emission. **e** LPRL emission: Excitons are captured by trap states and released over time through thermal activation, leading to persistent radiative emission

In indirect-type X-ray detectors, scintillators serve as critical photon transducers that convert high-energy X-ray photons into detectable ultraviolet/visible photons through three sequential processes: X-ray-to-light conversion, charge transport dynamics, and radiative luminescence (Fig. 1a). This section systematically summarizes the fundamental mechanisms governing MHP-based scintillator operation.

*Scintillation Process Fundamentals.* Under X-ray irradiation, the first process in MHP scintillation involves the interaction of incident radiation with the scintillator. X-rays interact with matter through a number of physical processes including photoelectric effects, Compton scattering, and pair production [51]. At energies below several hundred keV, photoelectric absorption typically prevails. Conversely, beyond this range, Compton scattering becomes the dominant mechanism, wherein incident high-energy photons impart a fraction of their energy to the material. During photoelectric absorption, electrons in the inner shells of atoms are excited and ejected, leaving the atom ionized. Subsequently, an intricate electron–hole multiplication process occurs, involving secondary X-ray emission, Auger non-radiative decays, and inelastic electron scattering. When the photon energy exceeds 1.02 MeV, electron–positron pairs will be generated. Below this threshold, electrons primarily lose energy through Coulomb scattering. Electron–electron scattering and Auger processes generate a large number of secondary electrons, leading to the creation of charge carriers with lower kinetic energy. Subsequently, the energy of these charge carriers is thermally dissipated through interacting with the phonons. During this process, numerous low-kinetic energy electrons and holes gradually accumulate at the conduction band and valence band, respectively. In the second stage, the generated electron and hole migrate toward luminescent centers, transferring energy in the process. This stage, referred to as the transport stage, spans nanoseconds to picoseconds (10^–9^–10^–12^ s). During the transport stage, the migrating charge carriers can be captured by defects in the scintillator such as ionic vacancies and grain boundaries or self-trapped in the crystal lattice, thus leading to non-radiative losses and possibly radiative recombination delay. This final stage involves the emission of scintillation photons. The stored energy in excited states is released via radiative emission or non-radiative decay. Radiative photon emission can occur via the recombination of electrons and holes with luminescent ions. Finally, the emitted light can then be detected and measured using various techniques, such as photomultiplier tubes, photodiodes, or charge-coupled devices (CCDs). The intensity and wavelength of the emitted light can provide information about the type and energy of the incident radiation, as well as the properties of the MHP scintillator. Here, four predominant luminescence mechanisms in MHPs are illustrated in Fig. 1b–e: exciton recombination, STE emission, thermally activated delayed fluorescence (TADF), and long-persistent radiative luminescence (LPRL).

*Exciton Recombination*. Exciton recombination is one of the most common luminescence mechanisms in MHPs. When perovskite materials are excited by X-rays, electrons are promoted from the valence band to the conduction band, forming electron–hole pairs, known as excitons. These excitons migrate within the material and eventually release energy through a radiative recombination process, emitting photons (Fig. 1b).

*STE emission*. STE are a luminescence mechanism commonly observed in soft lattice perovskites. In this process, when a material is excited by X-rays, electrons and holes are generated. Instead of forming free excitons, these charge carriers can become localized in a distorted local lattice environment, forming a bound state with lower energy. This localization enhances the overlap of the electron and hole wavefunctions, leading to longer lifetimes and broader emission spectra (Fig. 1c).

*TADF emission*. TADF is a special luminescence mechanism where triplet excitons are converted to singlet excitons through a process called Reverse Intersystem Crossing (RISC). In MHPs, TADF is observed in systems with a narrow singlet–triplet energy gap (Δ_EST_), such as Zr-based variants. When the material is excited by X-rays, electrons are promoted from the ground state (*S*₀) to the excited singlet state (*S*₁). The excited singlet state (*S*₁) can undergo intersystem crossing to form a triplet state (*T*₁). This non-radiative process involves a spin-state transition of the electron. Subsequently, the triplet state (*T*₁) can be thermally activated to return to the singlet state (*S*₁) through reverse intersystem crossing (RISC). This process is facilitated by the small energy difference between the triplet and singlet states. Finally, the singlet state (*S*₁) formed through RISC can then undergo radiative recombination, emitting a photon and returning to the ground state (*S*₀) (Fig. 1d).

*LPRL emission.* LPRL is a phenomenon where a material continues to emit light for a prolonged period after the X-ray source is removed. This luminescence mechanism is typically associated with defect states or trap states of the perovskites. When the material is excited by X-rays, electrons are promoted from the valence band to the conduction band, forming electron–hole pairs (excitons). Some of these excitons are captured by trap states, acting as trapping centers, within the material. The trapped excitons remain in these trapping centers for a significant period, which can range from seconds to hours. The depth and number of trapping centers determine the duration of the energy storage. Deeper and more numerous trap states can lead to longer persistence times. Over time, the trapped excitons can be thermally activated, meaning they gain enough energy to escape from the trapping centers. Once the excitons escape from the trap states, they can return to the valence band through radiative recombination, thereby emitting persistent luminescence (Fig. 1e).

## Intrinsic Physical Properties Modulation

Light yield and response time, as the core performance parameters of scintillators, critically determine the spatial resolution and detection limit of X-ray imaging [52]. Light yield, which reflects the efficiency of converting X-ray energy into visible light, directly enhances the signal-to-noise ratio (SNR) of imaging systems by reducing statistical noise, thereby improving spatial resolution. For instance, in medical CT scans, higher light yield enables the identification of microtumors or microcalcifications in soft tissues. Simultaneously, elevated light yield lowers the detection limit, allowing the system to capture low-dose X-ray signals, which is vital for radiation-sensitive applications such as mammography and pediatric imaging. Response time, governing the decay rate of scintillation emission, refers to how quickly the scintillator can emit light after absorbing X-ray energy. A fast response ensures image clarity in real-time angiography or high-speed industrial CT scans. Moreover, rapid response time enhances detection sensitivity by enabling precise temporal discrimination between weak signals and background noise, making it indispensable for time-resolved X-ray fluorescence imaging or transient process studies, such as in operando observations of battery charging/discharging. Therefore, by synergistically optimizing light yield and response time, next-generation scintillators can meet the dual requirements of emerging technologies like photon-counting CT (enabling energy discrimination and multispectral imaging), X-ray fluorescence tracking (trace element detection), and real-time industrial inspection (e.g., 3D printing process monitoring). In this section, we focus on strategies for enhancing light yield and modulating response time, aiming to optimize these intrinsic properties to meet the demands of advanced X-ray imaging technologies.

### Light Yield Enhancement

The pursuit of enhanced light yield in MHPs represents a critical pathway toward high-sensitivity X-ray detection. The light yield quantifies the efficiency of converting absorbed radiation energy into detectable light. Specifically, it measures the yield of UV/Visible photons for every unit of energy absorbed, a critical factor for scintillator performance. Typically, the yield of UV/Visible photons, denoted as *N*_ph_, generated during the scintillation process for each incoming X-ray photon's energy E, is quantified using following equation: *N*_ph_ = (*E*/*βE*_gap_) × SQ [53], which takes into account the scintillator band gap (*E*_gap_), the transmission efficiency of electron–hole pair energy to the luminescent center (*S*), and the quantum efficiency (*Q*) [51]. Besides, the parameter *β*, which represents the average energy required to generate one electron–hole pair, proves in a range of 2–3 in most scintillator. MHPs exhibit inherent advantages for light yield optimization compared to conventional scintillators, as evidenced by their narrow bandgaps (1.5–3.2 eV) versus LaBr₃ (5.90 eV), Bi₄Ge₃O₁₂ (4.9 eV), NaI (3.9 eV), and CsI (5.14 eV). The inverse correlation between bandgap and theoretical light yield suggests extraordinary potential (129,000–250,000 Ph MeV⁻^1^) [54], yet practical achievements remain constrained by several challenges, such as non-radiative recombination losses, obvious self-absorption processes with the crystals, optical loss during optical transmission process, etc. Recent advances address these limitations through three key approaches [1, 16, 55]: implementing confinement effects to enhance photon emission, increasing Stokes shift to minimize self-absorption, and enhancing crystallization to suppress non-radiative pathways.

#### Constructing Confinement Effect

The strategic construction of confinement effects has proven effective for enhancing light yield in MHP scintillators. By spatially localizing excitons and suppressing non-radiative recombination, confinement engineering significantly improves radiative efficiency. Two primary strategies are widely adopted: (1) synthesizing MHP nanocrystals (NCs) or quantum dots (QDs) to exploit quantum confinement and (2) designing layered perovskite structures to decouple light absorption and emission processes. This section systematically examines these strategies and their transformative impact on MHP scintillator performance.

##### Nano MHP

**Fig. 2:**
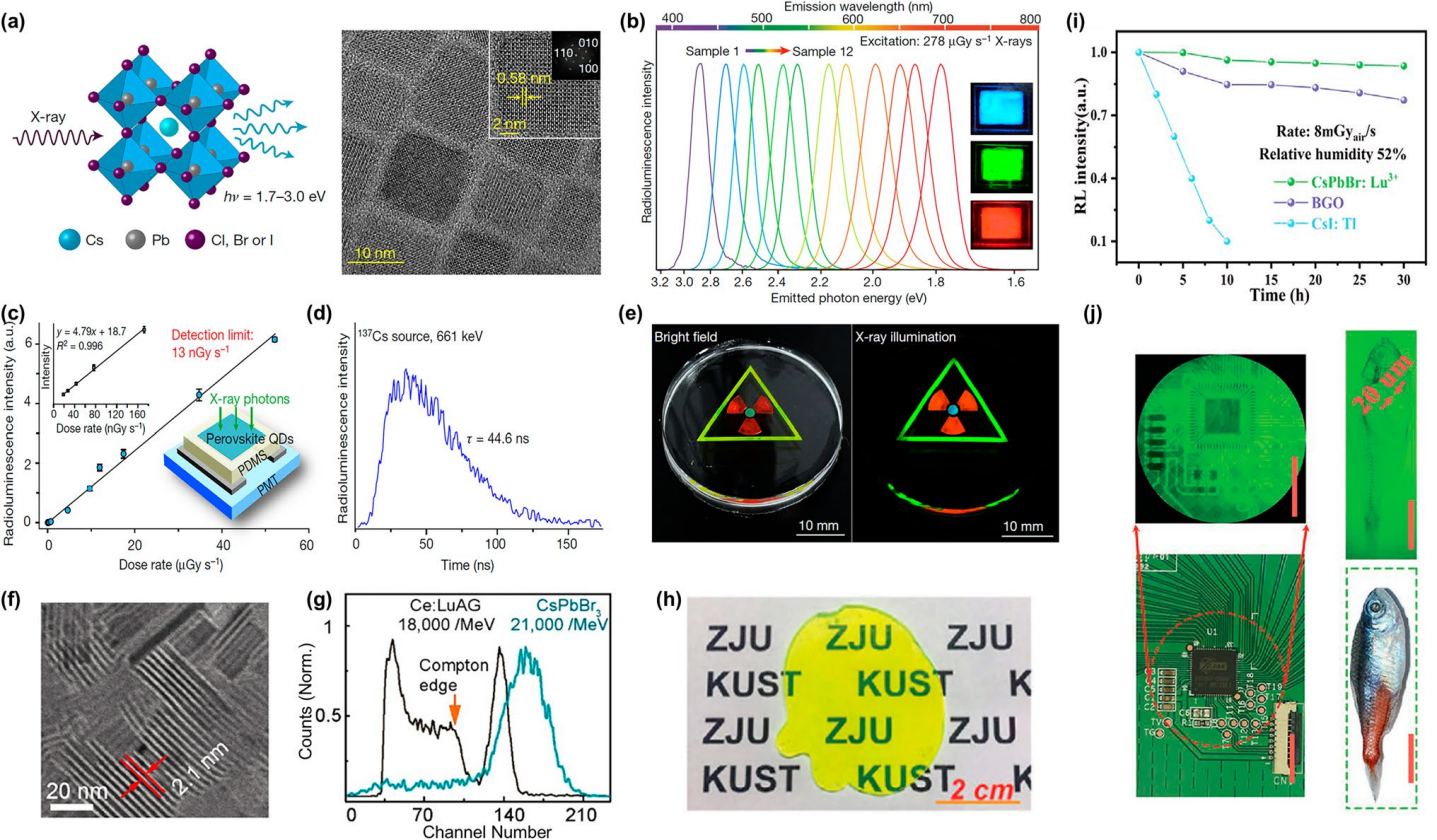
Scintillator performances of MHP NCs. **a** Structural schematic and corresponding TEM image of synthesized CsPbBr_3_ QDs. **b** Tunable X-ray-induced luminescence spectra of CsPbX_3_ QDs. **c** RL intensity versus X-ray dose rate for CsPbBr₃ QDs. **d** RL decay kinetics of CsPbBr_3_ QDs under ^137^Cs irradiation. **e** Multicolor X-ray scintillation from CsPbX₃ QDs: orange (CsPbBr₂I), green (CsPbBr₃), blue (CsPbClBr₂) [58]. **f** TEM image of assembled CsPbBr_3_ nanoplatelets. **g** Pulse height spectra comparing CsPbBr₃ nanoplatelets with LuAG:Ce scintillator [59]. **h** Photographs of CsPbBr_3_ CG. **i** Normalized RL intensity under prolonged X-ray exposure for CsPbBr_3_ CG, CsI: Tl, and BGO. **j** Photographs of a transistor panel in a cellphone and a fish recorded under daylight and X-ray irradiation, respectively [38, 60]

Reducing three-dimensional of 3D MHP to QDs represents an effective strategy for confining X-ray-generated excitons through spatial quantum confinement [56]. This approach enhances exciton wavefunction overlap by compressing carriers within dimensions below the Bohr radius, thereby significantly increasing exciton binding energy. This enhancement not only mitigates the impact of thermal energy on exciton stability but also augments the material's ability to efficiently convert and retain the energy from X-ray generated excitons. In 2017, Tang et al. [57] pioneered systematic investigations of CsPbBr_3_ QDs under diverse solvents, concentrations, and X-ray irradiation environments. Their study revealed a significant linear correlation between RL intensity and X-ray tube current, establishing MHP QDs as promising scintillator materials. Subsequent advancements by Liu et al. [58] demonstrated solution-processable CsPbX₃ QDs (X = Cl, Br, I) with tunable X-ray-induced emissions (Fig. 2a and b). Unlike conventional bulk scintillators, these nanostructures enable low-temperature processing and spectral tunability through anion composition engineering. These features allow the fabrication of flexible and highly sensitive X-ray detectors with a detection limit of 13 nGy_air_ s^−1^ (Fig. 2c), a fast decay time (Fig. 2d), and a high spatial resolution of 2.0 lp mm^−1^. Notably, the color-tunable perovskite CsPbX_3_ QDs scintillators can provide a convenient visualization tool for X-ray radiography (Fig. 2e). Sang et al. [21] further optimized CsPbBr_3_ QDs (9 ± 1.5 nm) using a solution chemistry method and fabricated a scintillator film through photopolymerization. The engineered nanostructure minimized light scattering while achieving a higher spatial resolution of 9.8 lp mm^−1^, a faster response time of 200 ns, and > 95% of photoluminescence quantum yield (PLQY). In addition to QDs, Omar F. Mohammed et al. [59] developed CsPbBr_3_ nanoplatelets scintillators (Fig. 2f) for high-resolution X-ray imaging screens, which can be readily cast into uniform, crack-free, large-area films with the necessary thickness. These two-dimensional (2D) architectures demonstrated an ultrahigh light yield (∼21,000 Ph MeV^−1^) (Fig. 2g) and sustained performance under prolonged irradiation. While these studies highlight the exceptional scintillation properties of MHP NCs, their practical implementation faces critical challenges. The inherent soft lattice structure of MHPs makes them susceptible to degradation under harsh conditions including high temperature, moisture, and intense irradiation. In X-ray imaging applications, such instability not only compromises spatial resolution, but also increases operational costs through frequent component replacement. These limitations underscore the imperative for developing robust encapsulation strategies.

Xu et al. [38, 60, 61] addressed these challenges through a self-limited growth strategy that embeds CsPbBr_3_ QDs within inorganic glass matrices (Fig. 2h). The encapsulation of CsPbBr_3_ QDs within glass matrix ensures complete isolation from the external environment, simultaneously blocking atmospheric/water infiltration and preventing lead leakage. This architecture enables groundbreaking performance with a low detectable dose of 50 nGy_air_ s^−1^ (Fig. 2i), and a spatial resolution of 16.8 lp mm^−1^ (Fig. 2j) is realized [38, 61]. The matrix-encapsulated design not only enhances operational stability but also addresses toxicity concerns, establishing a viable pathway for commercial deployment of perovskite-based radiation detectors.

##### Layered Structures

Layered perovskites, particularly 2D MHPs, exhibit a quantum well architecture comprising alternating inorganic halide layers and organic ammonium spacers [62]. In this sandwich structure, the organic layers act as barriers due to their higher energy gap, while the inorganic layers act as wells due to their lower energy gap. Thus, the excitons can be confined in the inorganic layers. Subsequently, excitons from the inorganic layer have a high binding energy and oscillator strength, which endow the excitons in 2D MHPs with high light yield. This section will discuss how such structural engineering enables advanced radiation detection.

**Fig. 3:**
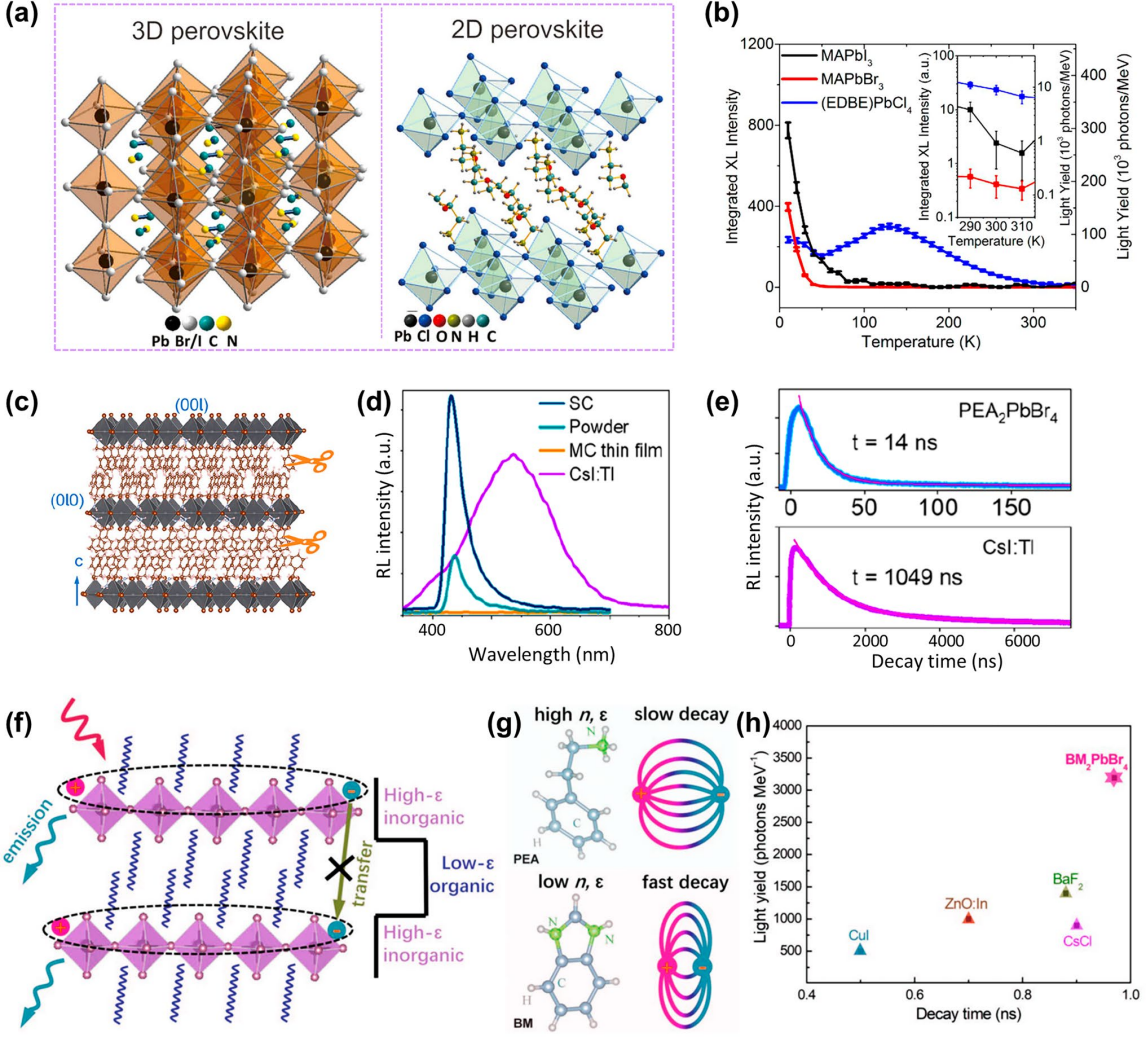
Scintillator performances of 2D MHPs. **a** Crystal structure representation of MAPbX_3_ (X = I, Br) 3D, and (EDBE)PbCl_4_ 2D MHPs. **b** Temperature dependence of the light yields of MAPbI_3_, MAPbBr_3_, and (EDBE)PbCl_4_ at various temperature [19]. **c** Crystal structure of the 2D layered (PEA)_2_PbBr_4_. **d** RL comparison of the (PEA)_2_PbBr_4_ SC, powder, and MC thin film and CsI: Tl SC under the same X-ray irradiation. **e** RL decay time comparison of (PEA)_2_PbBr_4_ SC and CsI:Tl [63]. **f** Crystal structures of 2D hybrid MHP with self-assembled 2D multiple quantum well structure. **g** Design strategy of enhancing exitonic dielectric confinement by choosing organic amines with low refraction index (n). **h** Comparison of the light yield of sub-nanosecond scintillators [64]

Soci et al. [19] pioneered comparative studies of 3D (MAPbI_3_ and MAPbBr_3_) and 2D (EDBE)PbCl_4_ perovskites under X-ray excitation (Fig. 3a). It is observed that low-temperature X-ray excited luminescence measurements have shown that the X-ray luminescence yield can be as high as ~ 120,000 Ph MeV^−1^ in (EDBE)PbCl_4_ at *T* = 130 K, and in excess of 150,000 Ph MeV^−1^ in MAPbI_3_ and MAPbBr_3_ at *T* = 10 K. While light yield of 3D perovskite MAPbI_3_ and MAPbBr_3_ is significantly reduced at room temperature (< 1,000 Ph MeV^−1^), the 2D MHP (EDBE)PbCl_4_ is less affected by thermal quenching (~ 9,000 Ph MeV^−1^ at room temperature) thanks to its large exciton binding energy (Fig. 3b). This demonstrated that the large exciton binding energy in 2D perovskite makes them more robust against thermal quenching compared to their 3D counterparts, opening a new door for the application of 2D MHPs in scintillators. Subsequently, researchers have persistently pursued the development of novel 2D MHP and investigated strategies to augment their light yield, thereby pushing the boundaries of their performance. For instance, Liu et al. [63] designed an airflow-controlled solvent evaporation system to grow an inch-sized 2D (PEA)_2_PbBr_4_ SC (Fig. 3c), resulting in high light yield of 73,226 Ph MeV^−1^ (Fig. 3d) and a fast response time of 14 ns (Fig. 3e). Additionally, capitalizing on these superior properties, a high-resolution X-ray imager was fabricated by incorporating a (PEA)_2_PbBr_4_ SC with dimensions of 57 × 41 mm^2^, and a high spatial resolution of 11.1 lp mm^−1^ is achieved. Liang et al. [40] demonstrated that the Mn^2+^-doped BA_2_PbBr_4_ 2D MHP demonstrates an ultrahigh sensitivity for X-ray, with a high X-ray scintillation light yield of up to 85,000 Ph MeV^−1^ and low detection limits of 16 nGy_air_ s^−1^.

Recently, Birowosuto et al. [65] engineered dual-cation 2D perovskites by incorporating benzylammonium (BZA) into phenethylammonium (PEA) frameworks. The lattice contraction and bandgap widening from BZA doping enhanced electron–hole transfer efficiency by 60%, yielding > 70% fast decay components. Liang et al. [66] synthesized (C_4_H_9_NH_3_)_2_PbBr_4_ (BPB) scintillators exploiting heavy-element inorganic layers for superior X-ray absorption. As a result, the BPB scintillator exhibited excellent performance, including a high light yield (22,000 Ph MeV^−1^) and fast response times (τ _decay_ = 10.3 ns) and high resolutions of 17.3 lp mm^−1^; these are among the highest resolution levels for 2D MHP scintillators. Beyond their application in X-ray detection, this perovskite exhibits promising potential for high-energy radiation detection, encompassing the detection of alpha particles (α-particles), neutrons, and gamma rays, thereby extending their utility across a broader spectrum of radiation detection scenarios.

Additionally, process optimization can further enhance their scintillator performance. Niu et al. [64] presented a novel strategy for developing sub-nanosecond 2D MHP scintillators through dielectric engineering. The introduction of benzimidazole (BM) effectively reduced the polarity and dipole moment of the organic molecules, leading to a decrease in the dielectric permittivity and an increase in exciton confinement (Fig. 3f, g). This approach resulted in a significantly large exciton binding energy of 360.3 ± 4.8 meV, a light yield of 3,190 photons MeV^−1^ (Fig. 3h), and an ultrafast emission decay time of 0.97 ns. Additionally, this scintillator also demonstrated excellent performance in detecting γ-rays, neutrons, and α-particles, with a theoretical coincidence time resolution (CTR) of 65.1 ps for PET applications.

#### Decreasing Self-absorption

Due to the need for substantial thickness to ensure complete X-ray attenuation, MHP experience reduced light output efficiency, as the emitted light is partially absorbed within the structure itself. This issue highlights the critical need for strategies that can reduce self-absorption via expand the Stokes shift. To address this challenge, researchers have been exploring various approaches to minimize self-absorption, such as the development of STE emission in novel MHP materials, the introduction of emitting centers, and constructing the ET process. The following subsections will delve into these approaches in detail.

##### Self-trapped Exciton Emissions

The quest to enhance scintillation light yield in MHP scintillators has placed significant emphasis on leveraging STE emissions. These materials exhibit a large Stokes shift and high PLQY, which are pivotal for efficient scintillation. During MHP emission process, excitons in MHPs strongly interact with phonons (lattice vibrations), leading to self-trapping and inhibition of their movement within the crystal. This results in the formation of local lattice deformations around the exciton. The key scintillation parameters of typical STE MHPs are summarized in Table 2.

**Table 2:** Key scintillation parameters of the typical STE MHPs

Materials	Structural dimension	Emission wavelength (nm)	Stokes shift (nm)	Light yield (Ph MeV^−1^)	Decay time	Detection limitation	Resolution (lp mm^−1^)	Published year and Refs
Rb_2_CuBr_3_	1D	385	20	91,056	41,400 μs	121.56 nGy_air_ s^−1^	–	2019 [4]
Cs_2_Ag_0.6_Na_0.4_In_1-y_Bi_y_Cl_6_	2D	600	230	39,000 ± 7000	16 μs	19 nGy_air_ s^−1^	4.4	2020 [67]
[BAPMA]Cu_2_Br_5_	0D	526	244	43,744	50.2 μs	74 nGy_air_ s^−1^	15	2023 [68]
Cs_2_HfCl_6_	2D	447	227	21,700	–	55 nGy_air_ s^−1^	11.2	2023 [69]
Cs_5_Cu_3_Cl_6_I_2_	1D	466	212	66,000	39 μs	11 nGy_air_ s^−1^	27	2024 [49]
CsPbCl_x_Br_3-x_:Yb^3+^	3D	1000	550	11,200	1242 μs	176.5 nGy_air_ s^−1^		2023 [70]
[DADPA]InCl_6_·H_2_O: Sb^3+^	0D	554	196	51,875	2750 μs	98.3 nGy_air_ s^−1^	25.15	2023 [71]
CsMnCl_3_	3D	630	200	13,400	376 μs	470 μGy_air_ s^−1^	4.0	2022 [72]
Cu_3_Cu_2_I_5_	0D	450	230	123,736	0.734 μs	183, 800 nGy_air_ s^−1^	6.8	2022 [41]
C_4_H_12_nCl_3_	0D	646	370	50,500	–	–	12.2	2022 [73]
CsPbBr_3_	3D	515	150	15,800	–	120 nGy_air_ s^−1^	12.5	2021 [38]
Cs_2_AgI_3_	1D	475	185	82,900	0.1928 μs	77.8 nGy_air_ s^−1^	16.2	2022 [33]
Cs_2_ZrCl_6_	2D	440	190	49,400	83.55 μs	65 nGy_air_ s^−1^	18	2022 [74]
Cs_2_AgI_3_	1D	500	190	27,000	0.288 μs	–	–	2021 [75]
Cu_3_Cu_2_I_5_:K^+^	0D	530	280	-	107.57 µs	63.5 nGy_air_ s^−1^	5	2022 [76]
Cs_3_Cu_2_I_5_:2% In^+^	0D	450	227	70,169	–	3.58 μGy_air_ s^−1^	15	2023 [77]
Cs_2_NaGdCl_6_:Tb^3+^	3D	470	205	39,100	–	41.32 nGy_air_ s^−1^	10.75	2024 [78]
Cs_2_ScCl_5_·H_2_O:Tb^3+^	0D	548	308	–	1516 μs	–	9.5	2024 [79]
Cs2AgIn_0.9_Bi_0.1_Cl_6_	2D	550	230	32,000	–	87 nGy_air_ s^−1^	12	2024 [80]
[FMPPA]ZnBr_4_	0D	500	180	29,300 ± 600	2.64 μs	352 nGy_air_ s^−1^	12.5	2024 [81]
(C_20_H_20_P)_2_SbCl_5_	0D	600	245	12,535	–	3160 nGy_air_ s^−1^	30	2024 [82]
CsCdMnBiCl	2D	580	220	34,450	490.41 μs	183.6 nGy_air_ s^−1^	16.7	2024 [83]
Eu:CsCaCl_3_	3D	430	65	9,300	0.991 μs	213.8 μGy_air_ s^−1^	30.0	2024 [84]
Cs_5_Cu_3_Cl_6_I_2_	1D	470	170	19,865	41.09 μs	152 mGy_air_ s^−1^	20	2024 [85]
CsPbCl_3_:Br_0.02_	3D	430	65	–	0.343 ns	–	4.0	2024 [86]
Cs_2_TeCl_6_	3D	575	180	38,523	4.88 μs	258 nGy_air_ s^−1^	15.9	2024 [87]
Cs_2_NaTbCl _6_	3D	548	306	–	2360 μs	79.09 nGy_air_ s^−1^	10	2024 [88]
CsPb_2_Br_5_	3D	700	320	24,160	–	162.3 nGy_air_ s^–1^	21	2024 [89]
Cs_3_Cu_2_Cl_5_	0D	510	195	95,000	112 μs	2.70 μGy_air_ s^−1^	105	2024 [90]
Cs_2_SnCl_6_:Te^4+^	3D	600	235	–	3.82 μs	132 nGy_air_ s^−1^	20	2023 [91]

**Fig. 4:**
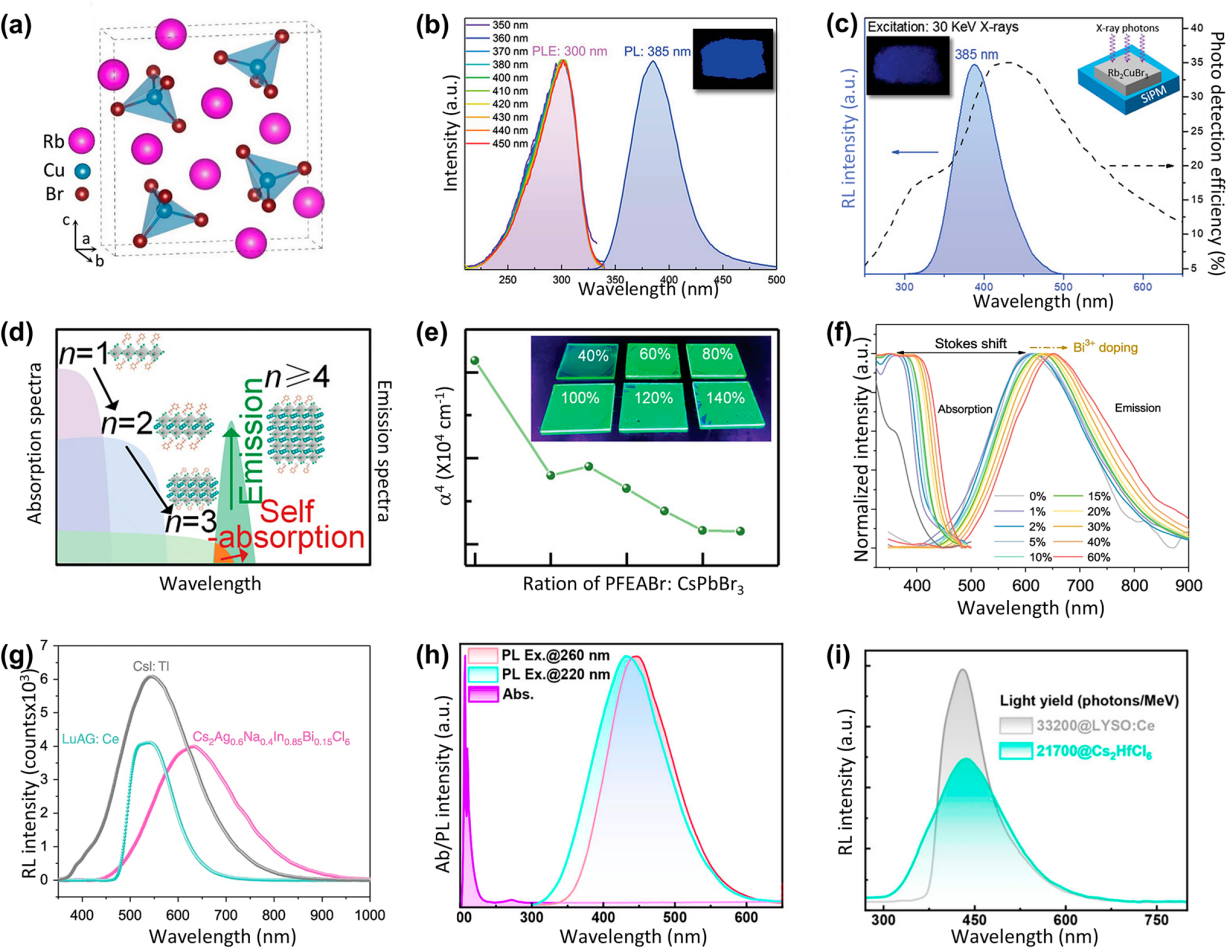
Scintillator performances of STEs MHPs. **a** Crystal structure of Rb_2_CuBr_3_. **b** PL spectrum of Rb_2_CuBr_3_ under 300 nm excitation and PLE spectra monitored at emission wavelengths from 350 to 450 nm. **c** RL spectrum of Rb_2_CuBr_3_ under 30 keV X-ray excitation and wavelength-dependent photon detection efficiency of the silicon photomultiplier (SiPM). **d** ET in quasi-2D MHPs contributes to a larger Stokes shift. **e** The calculated self-absorption coefficient of the quasi-2D perovskite film [92]. **f** RL spectra of Cs_2_Ag_0.6_Na_0.4_In_0.85_Bi_0.15_Cl_6_, LuAG: Ce and CsI: Tl scintillators. **g** Stokes shift of Cs_2_Ag_0.6_Na_0.4_In_1-y_Bi_y_Cl_6_ with different Bi^3+^ contents [67]. **h** Absorption and PL spectra of Cs_2_HfCl_6_. **i** RL spectra of Cs_2_HfCl_6_ film and LYSO: Ce [69]

STEs generally exist in materials with a soft lattice and strong exciton–phonon coupling. They are small polarons trapped by a local potential in the distorted lattice, which can be viewed as “excited-state defects.” When the exciton strongly couples to the phonon, the soft lattice will distort with the exciton self-trapped into a potential minimum it creates. Once the lattice is distorted, due to the lower energy, excitons will be quickly self-trapped and then relax back to the ground state accompanied by emitting photons with broad emissive bands and large Stokes shifts [53]. The discovery by Tang et al. of the lead-free halide Rb_2_CuBr_3_ scintillator highlighted the potential of 1D MHP with soft lattice structures to create STEs (Fig. 4a). These STEs not only exhibit a substantial Stokes shift of 85 nm (Fig. 4b) but also achieve an extraordinary PLQY of 98.6%, along with a record high light yield of ≈91,056 Ph MeV^−1^ (Fig. 4c).

Low-dimensional MHPs commonly exhibit STE emission due to their robust electron–phonon interactions. In these structure, photo-excited excitons possess significant binding energy, resulting in shared electron–hole bonds. Lei et al. developed the zero-dimensional hybrid cuprous halide [BAPMA]Cu_2_Br_5_, which promotes STEs formation via electron–phonon interaction, exhibiting a substantial Stokes shift of 244 nm and exceptional scintillator performances with a light yield of 43,744 Ph MeV^−1^ and a low detection threshold of 74 nGy_air_ s^−1^. Tang et al. [92] proposed an efficient ET mechanism within the quasi-2D perovskite structure to further reduce the self-absorption coefficient. They utilized the general formula L_2_A_n-1_B_n_X_3n+1_ for quasi-2D MHP, where large organic cations (L) segment the octahedral frame [BX_6_]^2−^, creating a "quantum well" that increases the electronic bandgap and exciton binding energy due to quantum and dielectric confinements. Consequently, the Stokes shift is successfully increased (Fig. 4d), and its self-absorption coefficient is reduced by threefold by controlling the phase distribution of varying *n* values (Fig. 4e). Furthermore, another representative STE emission family of low dimension MHP is alkali copper(I) perovskites, which has been demonstrated to be excellent scintillators with decent light yield and low detection limits, as evidenced by materials such as Cs_3_Cu_2_I_5_ [41], Cs_5_Cu_3_Cl_6_I_2_ [85], CsCu_2_I_3_ [93], etc. For instance, Wu et al. [94] doped Tl^+^ into Cs_3_Cu_2_I_5_ single crystal (SC), achieving a light yield of 150,000 Ph MeV^−1^, an extremely low afterglow of 0.17% at 10 ms, and low detection limit of 66.3 nGy_air_ s^−1^. This scintillator also possesses a remarkable energy resolution of 3.4% at 662 keV and an ultrahigh light yield of 87,000 Ph MeV^−1^ under ^137^Cs γ-ray irradiation.

Beyond low-dimensional MHPs, double perovskites also exhibit notable STE emissions. For instance, the large Stokes shift in the Cs_2_Ag_0.6_Na_0.4_In_1-y_Bi_y_Cl_6_ scintillators contributes to high PLQY and light yield up to 39,000 ± 7,000 Ph MeV^−1^ [67]. In this case, the incorporation of Bi^3+^ into the lattice not only enhances the X-ray absorption efficiency but also modulates the luminescence properties. As the Bi^3+^ concentration increases, the PL peak shifts to longer wavelengths due to bandgap narrowing, resulting in a substantial Stokes shift. This shift is attributed to the formation of STEs, which minimize self-absorption and promote efficient light emission (Fig. 4f, g). Shi et al. [69] constructed a novel double-perovskite scintillator, Cs_2_HfCl_6_, exhibiting a high light yield of 21,700 Ph MeV^−1^(Fig. 4h, i), a low detection limit of 55 nGy_air_ s^−1^, and a high resolution of 11.2 lp mm^−1^.

While STE emission effectively suppresses self-absorption by leveraging large Stokes shifts, its performance is inherently constrained by material-specific properties such as lattice softness and exciton-phonon coupling strength. For example, although the STE emission in low-dimensional MHPs can achieve high light yield, yet their emission wavelength and efficiency are still closely related to the material's dimensionality and composition, limiting design flexibility. To address this, researchers have further explored the strategy of doping luminescent ions, which introduces extrinsic activators to modulate ET pathways, thereby achieving self-absorption suppression in a broader range of material systems.

##### Doping Emitting Ions

The introduction of emitting ions represents a strategic approach to mitigate self-absorption in scintillators, thereby enhancing their radiation detection efficiency and sensitivity. This methodology capitalizes on the modified energy levels and tailored absorption–emission profiles of dopants to optimize the scintillation process. Particularly, the incorporation of transition metal ions, notably Mn^2^⁺, offers an alternative effective route to suppress self-absorption in MHPs scintillators, resulting in the enhancement of RL performances [95–100]. The key scintillation parameters of the Mn^2+^-doped MHPs are summarized in Table 3.

**Table 3:** Scintillation properties of the Mn^2+^-doped MHPs

Materials	Emission wavelength (nm)	Stokes shift (nm)	Light yield (Ph MeV^−1^)	Decay time	Detection limitation	Imaging resolution (lp mm^−1)^	Published year and references
Cs_2_CdBr_2_Cl_2_:Mn^2+^	495	210	64,950	70.74 μs	17.82 nGy_air_ s^−1^	12.3	2023 [101]
BA_2_PbBr_4_:Mn^2+^	610	222	85,000	727.83 μs	16 nGy_air_ s^−1^	10.7	2022
CsMnCl_3_	650	132	13,400	460.5 μs	470 μGy_air_ s^−1^	4.3	2023 [102]
Cs_3_Cu_2_I_5_:Mn^2+^	445	135	95,772	1.086 μs	–	–	2023 [103]
Cs_3_Cu_2_I_5_:Mn^2+^	565	185	65,000	–	49 nGy_air_ s^−1^	11.8	2023 [104]
Cs_2_NaBiCl_6_:Mn^2+^	586	67	28,350	1.22 ms	45.2 nGy_air_ s^−1^	14.76	2023 [105]
Cs_2_CdCl_4_:Mn^2+^	585	340	88,138	24.3 ms	31.04 nGy_air_ s^−1^	16.1	2023 [106]
Cs_3_Cu_2_I_5_:Mn^2+^	442	132	67,000	–	–	–	2019 [107]
SS-/RR-1:Mn^2+^	530	165	14,912	729 μs	58.1 nGy_air_ s^−1^	–	2023 [108]
Cs_7_Cd_3_Br_13_: Mn^2+^/Pb^2+^	576	248	41,000	125 ns	15.92 nGy_air_ s^−1^	10.9	2023 [109]
CsCdCl_3_:Mn^2+^	605	315	–	7.92 ms	–	12.2	2023
Cs_3_Cu_2_I_5_:Mn^2+^	562	272	71,000	1.2 μs	127.5 nGy_air_ s^−1^	16.2	2023 [110]
CsCu_2_I_3_:Mn^2+^	580	250	32,000	58.92 ns	80.2 nGy_air_ s^−1^	16.6	2023 [111]
CsCdCl_3_:Mn^2+^, Zr^4+^	520	95	–	–	–	12.5	2023 [112]
DA_2_PbBr_4_:Mn^2+^	620	365	35,292	–	108.17 μGy_air_ s^−1^	10	2023 [113]
MAPbCl_3_:Mn^2+^	600	260	451	1.5 ns	–	–	2020 [114]
CsCdCl_3_:Mn^2+^, Sb^3+^	600	346	74,828	–	29.9 nGy_air_ s^−1^	20	2024 [115]
Cs_2_ZnBr_4_:Mn^2+^	526	161	15,600	255.90 μs	1.16 μGy_air_ s^−1^	5.06	2023 [116]
Cs_4_CdBi_2_Cl_12_:Mn^2+^	580	220	34,450	800 μs	183.6 nGy_air_ s^−1^	16.7	2024 [83]
Cs_2_SnF_6_:Mn^2+^	630	165	3,000	6.97 ms	0.72 μGy_air_ s^−1^	22	2022 [117]
(C_38_H_34_P_2_)MnBr_4_	517	157	80,000	318 μs	72.8 nGy_air_ s^−1^	–	2019 [118]
[TPPen]_2_Mn_0.9_Zn_0.1_Br_4_	515	65	68,000	296.34 μs	204.1 nGy_air_ s^−1^	11.2	2023 [119]
(Br-BzTPP)_2_MnBr_4_	534	171	80,100	300 μs	30 nGy_air_ s^−1^	14.06	2024 [45]
(Br-PrTPP)_2_MnBr_4_	517	237	68,000	280 μs	45 nGy_air_ s^−1^	12.78	2024 [120]
(C_25_H_22_P)_2_MnBr_4_	512	258	78,937	330 μs	108.2 nGy_air_ s^−1^	25.61	2024
(ETP)_2_MnBr_4_	525	60	66,000	264 μs	1.7 μGy_air_ s^−1^	26.8	2024 [121]
HTP_2_MnBr_4_	520	155	38,000	318.11 μs	0.13 μGy_air_ s^−1^	17.28	2023 [122]

**Fig. 5:**
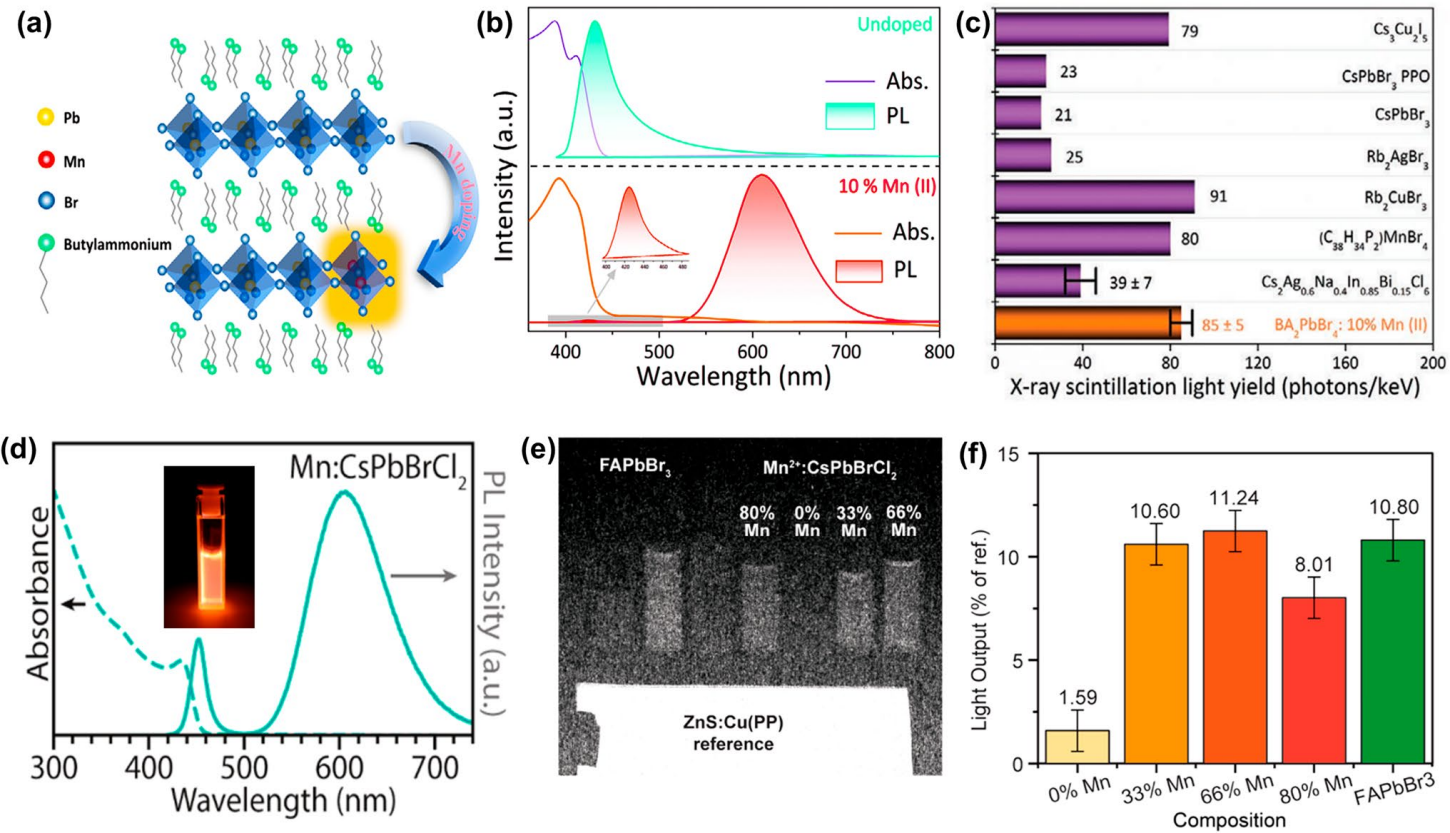
Scintillator performance of Mn^2+^-doped MHPs. **a** Schematic diagram of Mn^2^⁺-doped BA_2_PbBr_4_. **b** Absorption and PL spectra of undoped and 10% Mn^2^⁺-doped BA_2_PbBr_4_. **c** Comparison of the X-ray scintillation light yield of BA_2_PbBr_4_:10% Mn^2+^ with several previously reported MHP scintillators [40]. **d** Absorption and PL spectra of CsPb(BrCl)_3_: Mn^2+^ NCs. **e** Radiograph of CsPbBrCl_2_: Mn^2+^ NC under fast neutron irradiation as compared with FAPbBr_3_ NCs and a commercial ZnS: Cu screen. **f** Light output performance of FAPbBr_3_ NCs [124]

Liang et al. [40] demonstrated the efficacy of Mn^2^⁺ activation in 2D perovskites by developing BA₂PbBr₄:Mn^2^⁺ scintillators. The Mn^2^⁺ ions act as activators, inducing a pronounced Stokes shift that reduces self-absorption losses, which otherwise decrease light output efficiency (Fig. 5b). Optimized BA_2_PbBr_4_: 10%Mn^2+^ achieved an exceptional X-ray light yield of up to 85,000 Ph MeV^-1^ (Fig. 5c) and low detection limits of 16 nGy_air_ s^-1^. Further advancements by Chen et al. [123] revealed that Mn^2+^ ion doping significantly boosts the performance of 2D perovskite, phenylammonium cadmium chloride (PACC), achieving a quantum yield (PLQY) as high as 193%. In this case, Mn^2^⁺ ions integrated into the PACC structure act as ET mediators, facilitating Dexter ET from organic triplets to inorganic triplets, amplifying red phosphorescence. Moreover, Mn^2^⁺ ions effectively passivate deep trap states, curbing non-radiative decay of excitons and thereby enhancing overall luminescence efficiency. In addition to the X-ray detection, McCall et al. [124] extended Mn^2^⁺ doping applications to neutron imaging by developing CsPb(Br/Cl)₃:Mn^2^⁺ NCs. The as-obtained NCs exhibit an apparent large Stokes shift, which simultaneously improves PLQY and minimizes internal light loss (Fig. 5d–f). This breakthrough highlights Mn^2^⁺'s versatility in addressing self-absorption challenges across diverse radiation detection modalities.

**Fig. 6:**
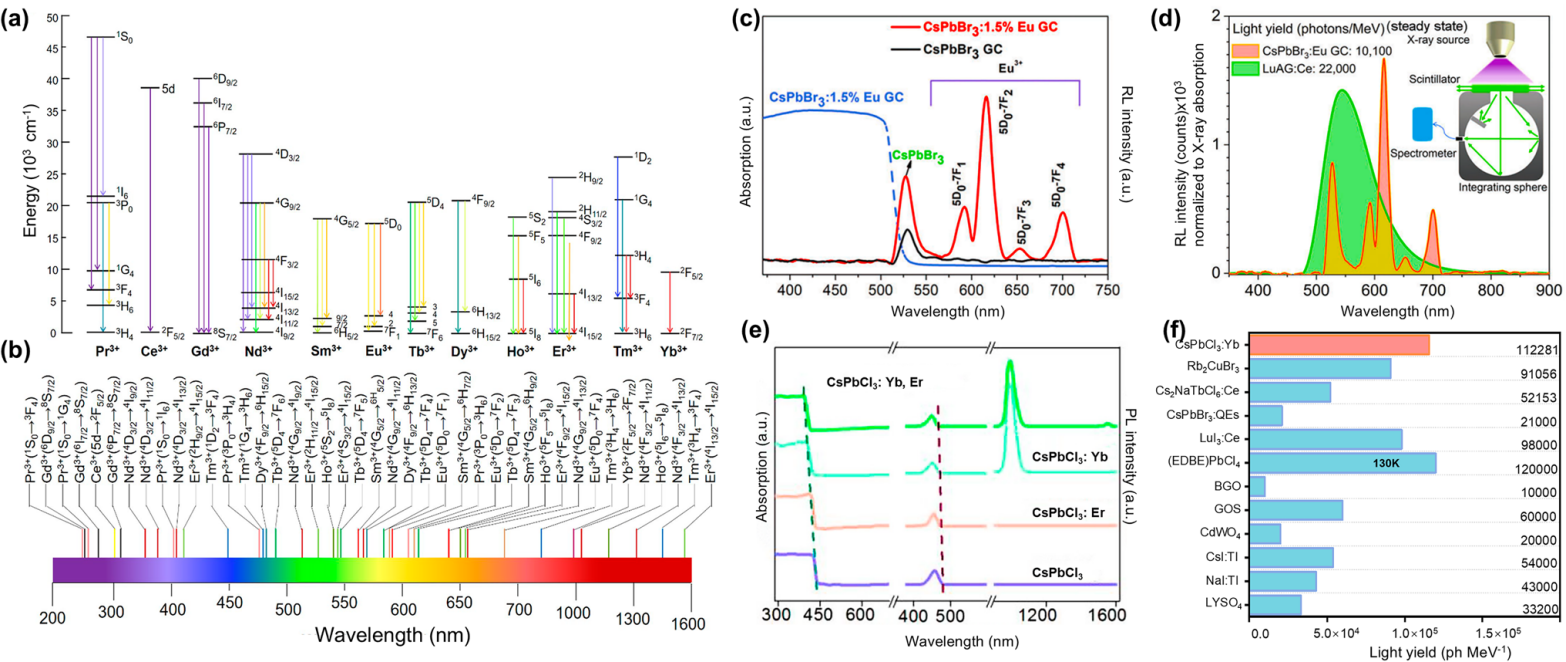
Lanthanide-doped MHP scintillators. **a** Partial 4f-energy level diagram for trivalent lanthanide activators. **b** Main luminescent transitions of the lanthanide activators in the electromagnetic spectrum [129]. **c** RL spectra of CsPbBr_3_ CG, CsPbBr_3_: Eu^3+^ CG under X-ray excitation. **d** Steady-state light yield calculation of CsPbBr_3_: Eu^3+^ CG, commercial LuAG: Ce. **e** Absorption spectra, visible emission spectra and near-infrared emission spectra (*λ*_ex_ = 365 nm) of lanthanide-free and -doped CsPbCl_3_. **f** Comparison of the light output between CsPbCl_3_: Yb.^3+^ scintillators and several typical scintillators [70]

Additionally, lanthanide ions have garnered significant research attention due to their distinctive spectroscopic properties. These ions, owing to their numerous energy levels derived from the 4f electrons, exhibit a broad spectrum of emission bands. This characteristic enables the tailoring of the RL emission wavelengths, serving as versatile additional luminescent centers. By strategically incorporating lanthanide ions, the RL spectra can be strategically broadened or fine-tuned, effectively mitigating self-absorption phenomena within the scintillating matrix. This approach not only optimizes light yield but also enhances the material's adaptability across diverse applications requiring specific wavelength emissions (Fig. 6a, b) [125–129].

In 2017, Song et al. [130] reported a significant boost in the overall PLQY of lanthanide-doped CsPbCl_3_ NCs over their undoped counterparts. This enhancement is largely attributed to the intrinsic luminescence of the lanthanide ions. Remarkably, even with ET from the excitons to the lanthanide ions, the PLQY of the excitonic emission in the CsPbCl_3_ NCs matrix itself experiences an increase. As for the X-ray properties, Xu et al. [37] pioneered a strategy that entails doping lanthanide Eu^3+^ ions into CsPbBr_3_ ceramic glass (CG) to effectively curb self-absorption phenomena. This approach achieves dual benefits: improved crystallinity/narrowed size distribution (enhancing spatial resolution) and introduction of Eu^3^⁺ red emission centers to suppress self-absorption (Fig. 6c, d). This innovative integration not only improves structural integrity but also optimizes the scintillation efficiency, highlighting the strategic value of rare-earth doping in advancing scintillator technology. Song et al. [70] further revolutionized X-ray detection through Yb^3^⁺-doped CsPbClₓBr_3-x_, leveraging quantum cutting to convert high-energy photons into multiple low-energy photons. This strategy led to a remarkable boost in the PLQY to an impressive 149%, accompanied by a substantial Stokes shift exceeding 550 nm (Fig. 6e). Finally, the scintillator achieved an exceptional light yield of approximately 11,2000 Ph MeV^-1^ (Fig. 6f), coupled with an impressively low detection limit of 176.5 nGy_air_ s⁻^1^. This dramatic improvement is attributed to the quantum cutting phenomenon, wherein Yb^3^⁺ ions efficiently transmute a single high-energy photon into multiple lower-energy photons.

Despite the success of ion doping, their practical application still faces many challenges, including precise control of doping concentration, uniform distribution of ions, and energy matching between host matrix and activator ions. Over-reliance on specific dopants may also restrict material universality. To break through these bottlenecks, ET channel construction in heterostructured composites (e.g., perovskite-dye hybrids) has emerged as a versatile strategy, which will be discussed in the next section.

##### Developing Composite Materials

**Fig. 7:**
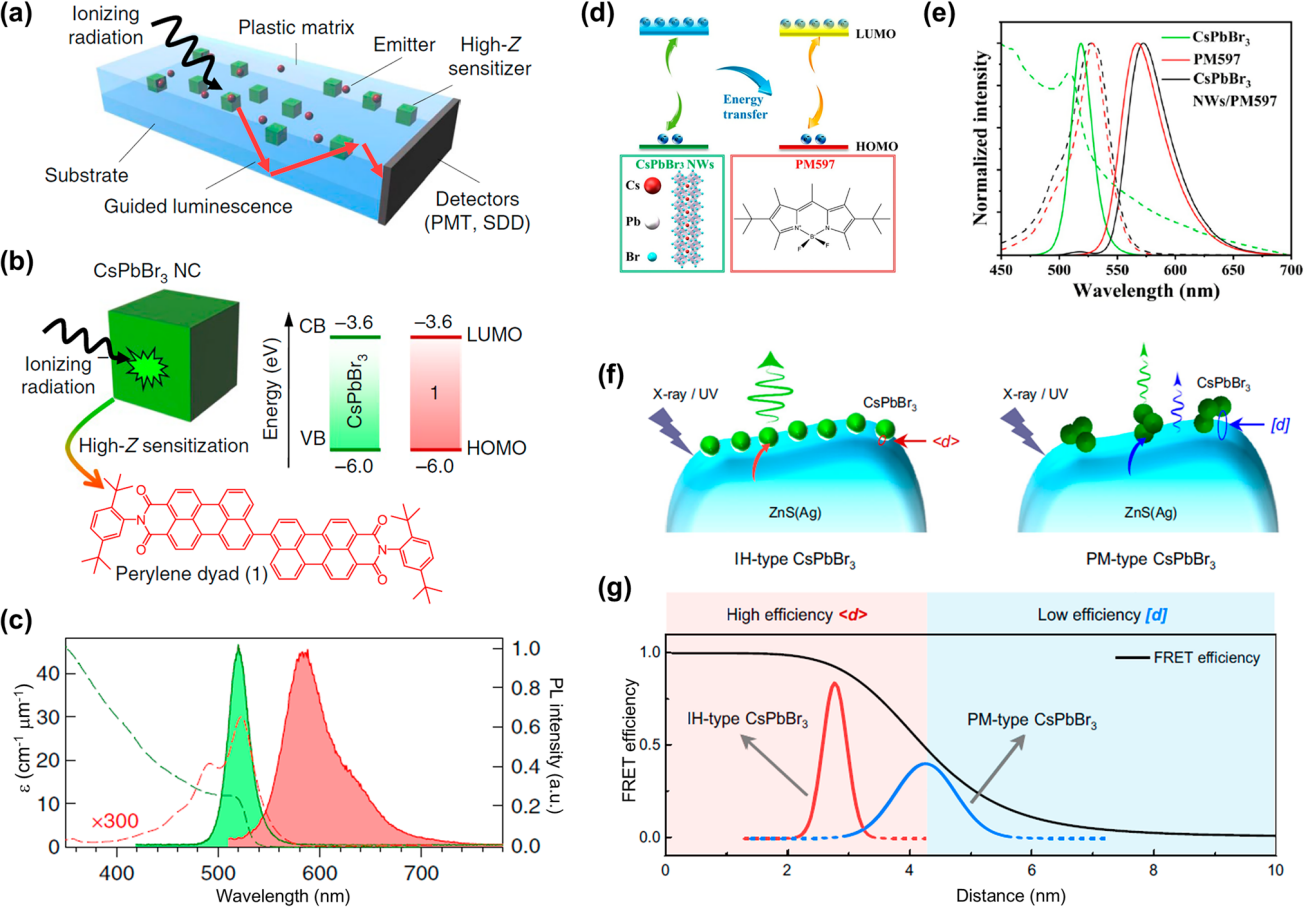
ET strategies for MHP scintillators. **a** Schematic depiction of composited scintillator for constructing the ET process. **b** Sketch of a CsPbBr_3_ NCs sensitizing the perylene dyad 1. **c** Molar extinction coefficient and PL spectra of CsPbBr_3_ NCs and perylene dyad 1 [131]. **d** Diagram of ET between the CsPbBr_3_ NWs and PM597 organic dye molecules. **e** The optical absorption and PL spectra of CsPbBr_3_ NWs, PM597 organic dye, and CsPbBr_3_ NWs/PM597 organic dye [132]. **f** Schematic of two kinds of structural configurations, IH-type CsPbBr_3_ and PM-type CsPbBr_3_. **g** Calculated FRET efficiency of IH-type CsPbBr_3_ and PM-type CsPbBr_3_ scintillator [133]

The construction of ET channels by synthesizing the MHP-based composite materials leverages the synergistic interaction between different components to facilitate efficient ET. 2020, Sergio Brovelli et al. [131] devised a strategy where CsPbBr_3_ NCs act as sensitizers for an organic emitter (perylene dyad 1) (Fig. 7a). The organic emitter is specifically designed to have a large Stokes shift between its absorption and emission spectra (Fig. 7b). This strategic design ensures minimal overlap between the emission spectrum of the sensitizer and its absorption spectrum, thereby reducing self-absorption (Fig. 7c). By efficiently transferring energy from the CsPbBr_3_ NCs to the organic emitter, the composted scintillator achieves a light yield of 9,000 Ph MeV^−1^ and a fast emission lifetime of less than 4 ns. Building upon this foundation, Li et al. [132] further demonstrated the versatility of this approach (Fig. 7d). By incorporating organic dyes, pyrromethene 597 (PM597 organic dye) for example, into purified CsPbBr_3_ nanowires (NWs), they achieved effective ET, leading to a 60 nm red-shift in RL and accelerated RL recombination rates (Fig. 7e). Consequently, the light yield was enhanced by approximately 4.1 times compared to pure perovskite NWs scintillators. This approach yielded a low detection limit of 152 nGy_air_ s^−1^ and an impressive resolution of 11.5 lp mm^−1^.

Beyond mere composite formation, optimizing the ET process through refinement of fabrication techniques is essential. Yang et al. [133] designed multi-site ZnS(Ag)-CsPbBr_3_ heterostructures to modulate the ET process (Fig. 7f, g). They found that external energy from ZnS(Ag) can effectively transfer to CsPbBr_3_ based on the non-radiative Förster resonance energy transfer (FRET), which is crucial for inhibiting photon self-absorption. This efficient FRET process resulted in a light yield of 40,000 Ph MeV^−1^ for the heterostructure (IH)-type CsPbBr_3_ scintillator, which is significantly higher than the 30,000 Ph MeV^−1^ for the physically mixed (PM)-type CsPbBr_3_ scintillators. Finally, the IH-type scintillator exhibits a short radioluminescence (RL) decay time of 36 ns and a high spatial resolution of 30 lp mm^−1^, enabling high-speed X-ray imaging at 200 frames s^−1^.

#### Enhancing Crystallinity

Effectively enhancing the crystallinity of MHP is also considered at a significantly for improving their light yield. By refining the crystal growth process and reducing defects, such as grain boundaries and impurities, the scintillator material becomes more efficient at converting incoming radiation into detectable light. This is because better crystallinity minimizes non-radiative recombination events, which can otherwise lead to energy loss without photon emission. Consequently, a more uniform and defect-free crystal structure allows for a higher probability of radiative recombination, resulting in an increased number of photons being produced per unit of energy deposited, thereby enhancing the overall light yield and sensitivity of the MHP scintillator. By employing a secondary annealing process, amorphous clusters are given sufficient time to rearrange, thereby eliminating amorphous clusters. For instance, Huang et al. [136] demonstrated the effectiveness of solvent annealing in enhancing the crystallinity and grain size of organometal trihalide perovskite, specifically methylammonium lead trihalide perovskite (CH_3_NH_3_)PbX_3_. Liu et al. [137] also demonstrated that by using the two-stage annealing strategy to improve the crystallinity of CH_3_NH_3_PbI_3_ films. However, conventional annealing risks material decomposition or grain aggregation, which may degrade device performance.

**Fig. 8:**
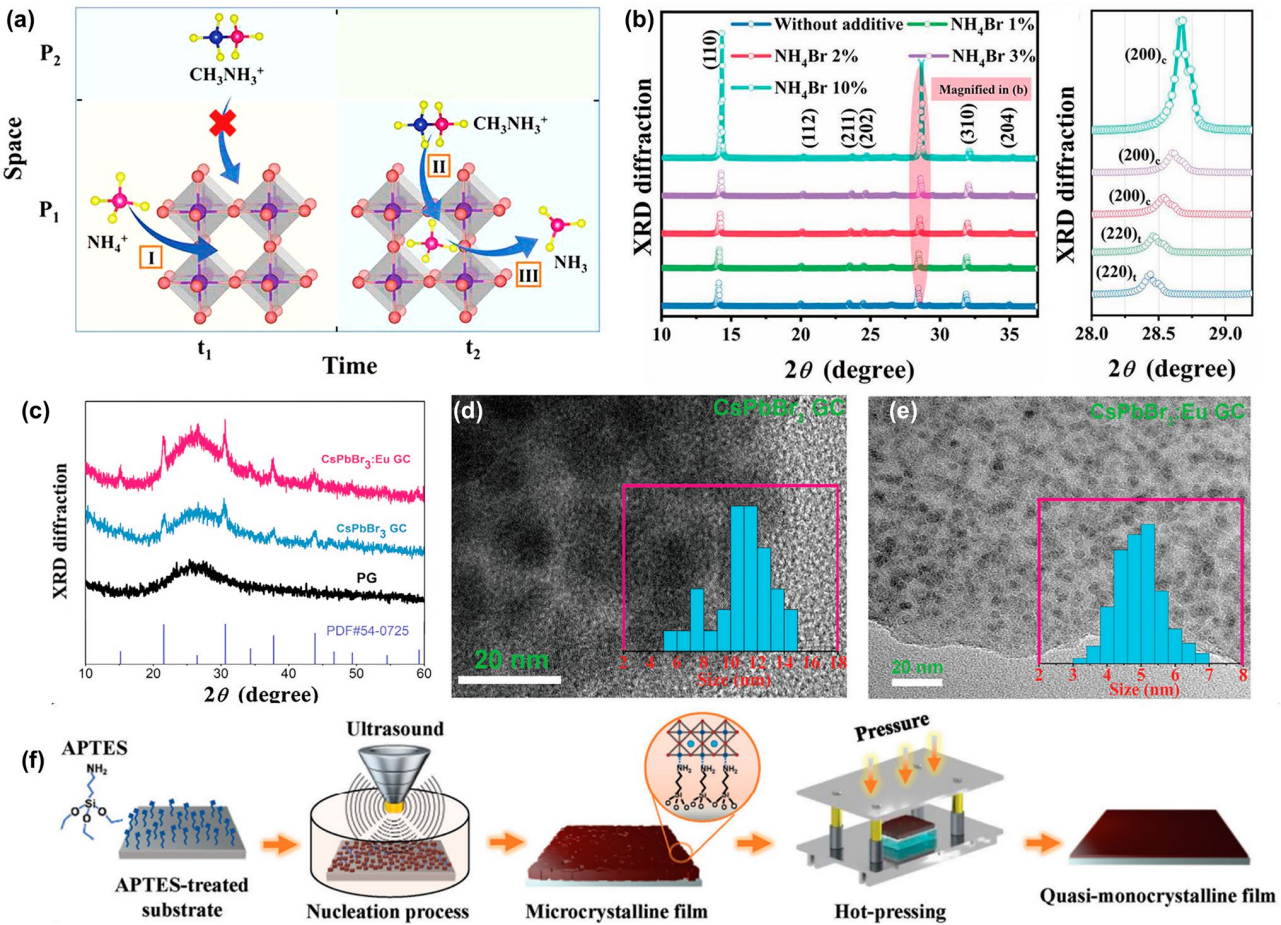
Crystalline enhancement strategies for MHP scintillators. **a** Schematic diagram of the A-site management strategy. **b** XRD patterns of MA-based MHP films with varying NH₄Br concentrations [134]. **c** XRD patterns of precursor glass (PG), CsPbBr_3_ and CsPbBr_3_: Eu^3+^ CG. TEM images of **d** CsPbBr_3_ and **e** CsPbBr_3_: 1.5%Eu.^3+^ CG [37]. **f** Schematic illustration of the fabrication process for MHP microcrystalline film via ultrasound-assisted crystallization and hot pressing [135]

Furthermore, site engineering has also been demonstrated to enhance the crystallinity of MHP, as it involves strategically manipulating the chemical composition and structure of the MHP material. By carefully selecting and incorporating specific ions or molecules into the MHP lattice, the crystal quality can be improved; defects can be reduced. Zhang et al. [134] proposed an innovative approach to enhance crystallinity of hybrid organic–inorganic perovskite, CH_3_NH_3_I, through a strategic A-site management technique (Fig. 8a). In this case, the strategy involves the introduction of an A-site placeholder cation, NH_4_^+^, to offset the deficient methylammonium (MA^+^) precipitation by occupying the cavity of Pb-I framework, thereby promoting to offset the precipitation rate discrepancy between the inorganic PbI_2_ and the organic MA^+^ components. The enhanced intensity and reduced full-width at half-maximum of the (110) diffraction peak from 0.18° to 0.13° indicate that NH_4_^+^ addition significantly improves the MHP’s crystal quality and preferred orientation (Fig. 8b), and then result in the reduction in defect density and prolonged carrier lifetime. This strategy is also demonstrated to be universal for other mixed-cation MHP systems, providing a significant advancement in the synthesis of high-quality MHP materials. Yuan et al. [138] reported that the introduction of rubidium ions (Rb^+^) into the precursor solution significantly boosts the crystalline quality of phenylethylammonium (PEA^+^)-based Ruddlesden–Popper (RP) MHPs. It is demonstrated that the added Rb^+^ ions are found to accumulate at the crystal growth front, forming a Rb^+^-rich region that acts as a mild inhibitor, slowing down the absorption and diffusion of organic cations and thus improving the crystalline quality. Wang et al. illustrated that adopting an impurity doping strategy also significantly improves the crystallinity of lead-free MHP Cs_2_AgIn_x_Bi_1-x_Cl_6_. Through meticulous adjustment of the In^3+^ to Bi^3+^ ratio, they fabricated Cs_2_AgIn_0.9_Bi_0.1_Cl_6_ MHP films with exceptional scintillation properties, with a remarkable light yield of up to 32,000 Ph MeV^−1^ and an impressive X-ray detection sensitivity down to 87 nGy_air_ s^−1^. In 2021, Yang et al. [37] successfully showed that integrating lanthanide ions, specifically Eu^3+^ ions, into a CsPbBr_3_ glass–ceramic (GC) matrix significantly enhances the crystallization process (Fig. 8c), leading to a more homogeneous dispersion of the CsPbBr_3_ CG (Fig. 8d, e). This uniform distribution not only mitigates light scattering but also boosts the photoluminescence intensity of the CsPbBr_3_ CG. Ultimately, this finding revealed that the CsPbBr_3_: 1.5%Eu^3+^ CG scintillator achieved an impressive estimated light yield of 10,000 Ph MeV^−1^, highlighting a substantial advancement in scintillator performance by site engineering.

Further optimization of the crystallinity can be also achieved through controlling the nucleation process. Kuang et al. [135] developed a high-quality microcrystalline thick film of mixed-cation perovskite MA_0.42_FA_0.58_PbI_3_ by manipulation the nucleation process. They utilized an ultrasound-assisted crystallization method combined with a hot pressing technique for the crystallization control (Fig. 8f). Ultrasound ensured homogeneous nucleation of microcrystals, while subsequent hot pressing eliminated grain boundaries and voids through plastic deformation. This dual approach enhanced carrier mobility and mobility lifetime products by 13-fold and 18-fold, respectively, ultimately achieving exceptional detector sensitivity (1.16 × 10⁶ μC Gyair⁻^1^ cm⁻^2^) and ultralow detection limits (37.4 nGyair s⁻^1^).

### Response Time Modulation

Scintillators are materials that convert high-energy radiation into visible light, and their decay time is a critical parameter affecting the response dynamics and overall performance of detection systems. The decay rate of the luminescence center is primarily determined by the transition of the magnetic dipole moment from the excited state back to the ground state. This rate can be augmented by non-radiative quenching or ET processes that divert from the excited state. In the simplest case of exponential decay, the emission intensity *I*(*t*) can be expressed as *I*(*t*) = exp(− *t*/*τ*), where *τ* is the decay time. It is noteworthy that in practical scenarios, the decay time of the scintillator is sometimes approximated by a simple 1/e or 1/10 decay time, which is the duration for the light intensity post-high-energy excitation to drop to 1/e or 1/10 of its initial value. In the realm of X-ray detection, the significance of both rapid and delayed response times cannot be overstated [20]. Rapid response times are crucial for applications demanding high temporal resolution, particularly in high-definition dynamic and real-time X-ray imaging scenarios. Conversely, delayed response times offer distinct advantages in specific detection scenarios, such as non-destructive testing in complex environments, including nuclear radiation dosimetry and the examination of intricate structures. In this section, the MHP scintillators in both fast and delayed response time detection are comprehensively discussed.

#### Fast Response Time

A rapid decay time is an extremely desirable trait for advanced radiographic technologies such as Time-of-Flight Positron Emission Tomography (TOF-PET) and real-time X-ray imaging. In TOF-PET, where the precise timing of photon detection from positron annihilation is crucial, a swift decay of the scintillation signal significantly enhances image resolution and SNR. By capturing and differentiating the arrival times of these photons with high precision, the spatial resolution of PET scans improves, allowing for more accurate localization of the radiotracer within the body, thereby increasing diagnostic accuracy and efficiency. In the field of real-time X-ray imaging, including applications in medical surgeries, industrial inspection, and airport security, a fast response from the scintillator enables continuous and rapid image updates, essential for capturing dynamic processes in real time. It minimizes motion artifacts and enhances image clarity and contrast, allowing for immediate and accurate assessments by medical professionals and inspectors. Furthermore, in applications requiring high frame rates, such as cardiovascular imaging, a swift decay ensures no residual signal between frames, preventing image blurring and ensuring crisp, continuous imaging. Thus, the development and implementation of scintillators with fast decay times are pivotal advancements driving the evolution of these sophisticated imaging technologies, enhancing diagnostic precision, and treatment effectiveness. Generally, decay time is an intrinsic property of scintillating materials, which is determined by the material's composition and structure. Previous work has established that high carrier mobility and short carrier lifetimes, which are desirable for fast scintillation response, are predominantly found in lead-based MHP materials. Therefore, we summarized the recent reported lead-based MHPs as scintillators and the corresponding strategies to further optimize light yield, decay time.

In 2018, Heo et al. [21] revolutionized a CsPbBr_3_-based X-ray detector by incorporating the CsPbBr_3_ NCs with methyl methacrylate (MMA) and a photo-initiator, boasting superior spatial resolution (9.8 lp mm^−1^ at an MTF of 0.2), rapid response times of approximately 200 ns, and comparable stability under intense X-ray exposures exceeding 40 Gy_air_ s^−1^. Simultaneously, Liu et al. [58] reported the as-obtained CsPbBr_3_ NCs via solution method, exhibiting a very fast response (*τ* = 44.6 ns) upon ^137^Cs source excitation, making them ideal for dynamic real-time X-ray imaging. To optimize the decay time or light yield, researchers have also delved into the exploration of fabrication processes for further refinement. For instance, Xu et al. [22] doped CsPbBr_3_ NCs with lanthanide ions, Lu^3+^, which resulted in a significant increase in the internal quantum efficiency from 25.67 to 65.7%. Consequently, the optimized CsPbBr_3_:Lu^3+^ NCs exhibit a fast decay time (*τ* = 27 ns). This enhancement is attributed to the effective improvement in the crystallinity of CsPbBr_3_ NCs and the enhanced X-ray absorption efficiency. Lee et al. [139] proposed a biphasic MHP scintillator formed by introducing CsPb_2_Br_5_ into CsPbBr_3_ to improve the scintillator behaviors. This approach mitigated surface defects on CsPbBr_3_, thereby enhancing photostability, hydrophobicity, thermal resilience, and luminescence. This optimized dual-phase material exhibited a PLQY of 65.2%, a swift response time of 2.93 ns, a remarkable light yield of 19,200 Ph MeV^−1^, and an outstanding spatial resolution of 8.19 lp mm^−1^. Aside from CsPbBr_3_, there are also other emerging fast decay time scintillators, such as organic–inorganic hybrid MHPs. In 2019, Mykhaylyk et al. [140] demonstrated that MAPbBr_3_ exhibits rapid and intense scintillation at cryogenic temperatures (50–130 K). At 77 K, the material achieved a light yield of 90,000 ± 18,000 Ph MeV⁻^1^, which further increased to 116,000 ± 23,000 Ph MeV⁻^1^ at 8 K. Notably, the decay components feature ultrafast (sf) and slow (ss) components of 0.1 ns and 1 ns, respectively, indicating the potential for achieving high temporal resolution. Muhammad et al. [141] advanced the field by optimizing the synthesis process for MAPbBr_3_ SCs, yielding millimeter-sized crystals with high quality. The optimized crystals displayed a remarkable scintillation response with a dominant ultrafast decay component of 0.52 ns (82.2%) and a secondary component of 1.80 ns (17.8%), emphasizing the pivotal role of synthesis optimization in boosting the scintillation properties of MHPs.

Additionally, 2D lead-based MHPs and their derivatives have garnered significant attention due to their unique layered structures, which are beneficial in reducing non-radiative recombination and potentially enabling faster response times. For instance, (PEA)_2_PbBr_4_ scintillator stands out as an optimal scintillator, combining high light yield with rapid nanosecond decay times. Pioneering the use of (PEA)_2_PbBr_4_ as a scintillator, Kishimoto et al. [142] demonstrated a light yield of 10,000 Ph MeV^−1^ and a swift fluorescence decay time of merely 9.9 ns. Utilizing a 0.9-mm-thick (PEA)_2_PbBr_4_ crystal as an X-ray detector, they successfully detected nuclear resonance scattering in N61i through synchrotron radiation, achieving a temporal resolution of 0.7 ns and a high detection efficiency of 24%. This study underscores the potential of 2D lead-based MHPs scintillators in sub-nanosecond time-resolved X-ray measurements. Building on this foundation, the library of 2D lead-based MHPs as scintillator materials was improved research. For example, Liu et al. [72] successfully synthesized 2D lead-based MHPs (C_4_H_12_N)_2_PbBr_4_ (BA_2_PbBr_4_), which exhibited a fast decay time of 2.66 ns and demonstrated a high resolution of approximately 1 lp mm^−1^ in neutron imaging. Mohammed et al. [66] introduced the organic–inorganic hybrid MHP (C_4_H_9_NH_3_)_2_PbBr_4_ (BPB) as a scintillator for both fast neutron and X-ray detection. The hydrogen-rich organic component in BPB acts as a fast neutron converter, generating detectable recoil protons, while the heavy atom-rich inorganic part efficiently deposits the energy of charged recoil protons and provides a large X-ray cross section. The as-obtained BPB scintillator had a decay time of 10.3 ns and showcased high-resolution imaging of about 1 lp mm^−1^ in neutron imaging and 17.3 lp mm^−1^ in X-ray imaging. Liu et al. [63] employed an airflow-controlled solvent evaporation system to grow inch-sized 2D (PEA)_2_PbBr_4_ SCs, which demonstrated a high light yield of 73,226 Ph MeV^−1^ and a rapid response time of 14 ns. Recently, Niu et al. [64] designed a 2D lead-based MHPs by intercalating benzimidazole (BM) with a low dielectric constant between [PbBr_6_]^2−^ layers. The resulting BM_2_PbBr_4_ single crystal SCs exhibited a remarkably large exciton binding energy of 360.3 ± 4.8 meV, leading to an ultrafast emission decay time of 0.97 ns, which is the smallest among all MHP materials. The material's theoretical coincidence time resolution was calculated to be 65.1 picoseconds (ps), indicating its potential for high-speed imaging applications.

#### Delayed Response Time

**Fig. 9:**
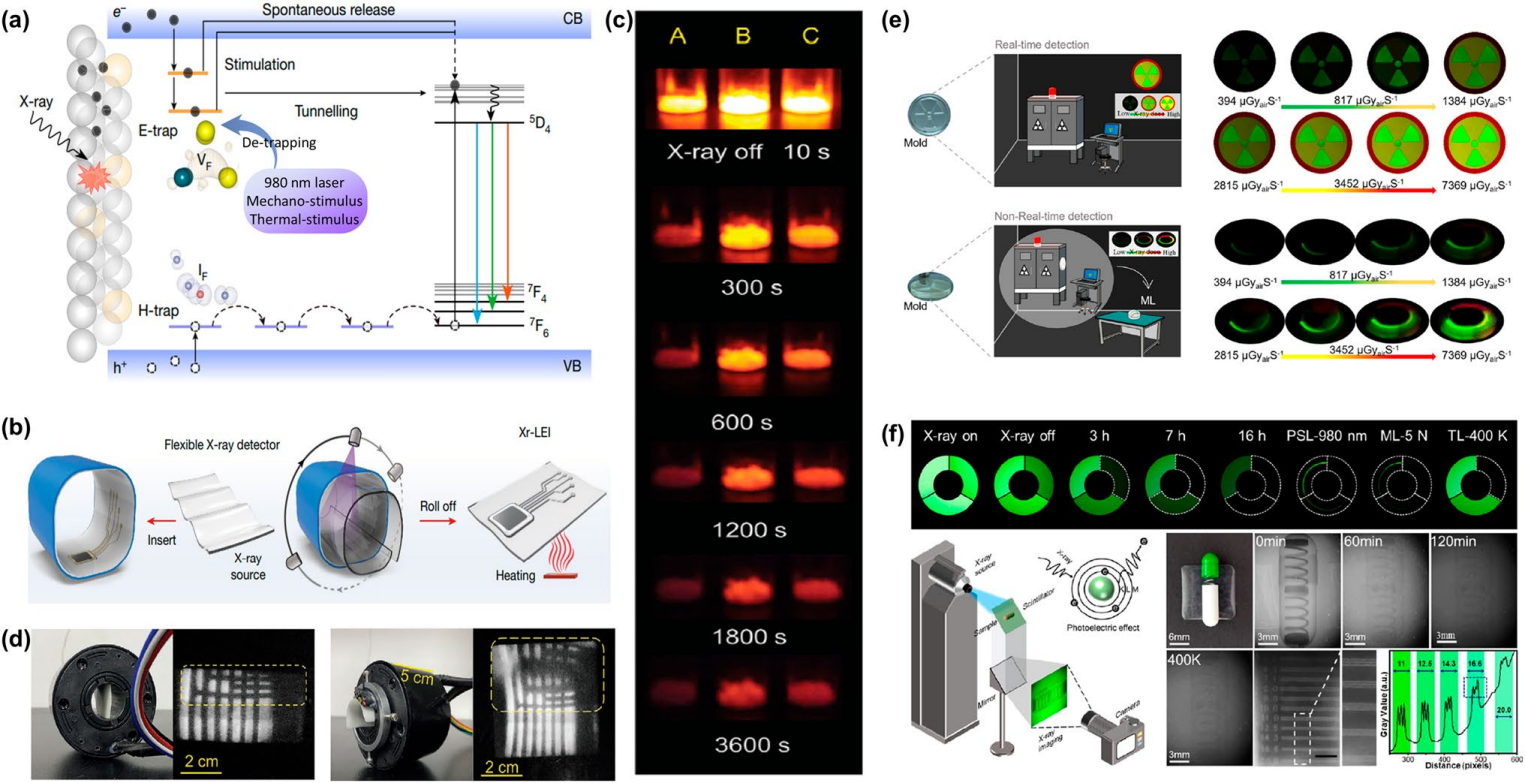
Delayed X-ray performance of the scintillators. **a** Mechanism diagram of X-ray-induced multi stimulus-induced de-trapping process. **b** Schematic diagram showing non-destructive inspection of 3D curved objects enabled by X-PersL behavior of the scintillators. **c** X-PersL photographs of A) CsCdCl_3_: 5%Mn^2+^, B) CsCdCl_3_: 5%Mn^2+^, 0.1%Zr^4+^, and C) CsCdCl_3_: 5%Mn^2+^, 0.1%Ti^4+^. **d** TSL images of annulus electric conduction link using the flat panel (left) and curved panel CsCdCl_3_: 5%Mn^2+^, 0.1%Zr^4+^@PDMS film (5 cm × 2.5 cm). The image read out temperature is 100 °C [144]. **e** Schematic diagram and photographs of the as-obtained NaLuF_4_: Ho^3+^ NCs or real-time and delayed-time (ML) of X-ray dose detection [145]. **f** Photographs of the time-dependent X-PersL, PSL, and TSL features of the as-obtained NaLuF_4_: Tb.^3+^ NCs [109]

Delayed imaging detection is an indirect imaging technique that involves the temporary storage of incident radiation energy and its subsequent release under external stimulation, such as pressure, temperature, or laser, resulting in delayed mechanical luminescence (ML), thermally stimulated luminescence (TSL), and photo-stimulated luminescence (PSL) (Fig. 9a). These luminescence phenomena are special in that, after the excitation light source is removed, the scintillator materials can maintain a certain level of luminescence in a dark room under external stimuli, giving delayed imaging detection potential applications in long-term optical information storage. Thus, delayed response time of the scintillator ensures that the scintillator material continues to emit light after the X-ray source is off, effectively extending the imaging time window. In 2020, Xiaogang Liu et al. [143] synthesized NaLuF_4_: Tb^3+^ NCs that exhibited persistent luminescence lasting for several days after X-ray irradiation was stopped. By combining the persistent X-ray luminescence performance with advanced computer graphics, they developed high-resolution 3D X-ray luminescence emission imaging technology, demonstrating the ability to overcome spatial detection constraints by leveraging the temporal dimension and achieve more precise acquisition of detection data from intricate geometries (Fig. 9b).

Xia et al. [144] achieved a significant breakthrough by first observing X-ray storage phenomena in MHPs, CsCdCl_3_: Mn^2+^, R^4+^ (R = Ti, Zr, Hf and Sn), after X-ray irradiation. It is demonstrated that X-ray storage capability can be effectively improved via site occupy manipulation and heterovalent substitution (Fig. 9c). Heterovalent substitution in Cd sites by R^4+^ broadens trap distributions and enhanced X-PersL performance. Finally, the integration of optimized storage scintillators onto flexible substrates has paved the way for 3D X-ray imaging under thermal stimulation (100 °C heating treatment) (Fig. 9d), effectively surpassing the constraints of traditional flat panel detectors. This work also demonstrates that the effective manipulation of trap centers through the introduction of impurity ions substitution can significantly enhance RL storage capacities. Subsequently, the strategic co-doping Dy^3+^ and Ag^+^ ions in Cs_2_NaLuCl_6_ crystals by occupying Na lattice sites showcases notable X-PersL characteristics, highlighting the effectiveness of this approach [74, 146]. Moreover, the Tb^3+^-doped Cs_2_NaScCl_6_ SC exhibits an exceptional X-PersL lasting up to 12 h upon X-ray excitation termination, allowing it to be used in a radiation storage battery which has a linear scaling capacity with radiation does or intensity. These work demonstrated that the effective construction of trap centers through the introduction of impurity ions substitution has significantly enhanced RL storage capacities, leading to improved delayed detection performance and opening new avenues for flexible 3D X-ray imaging under thermal stimulation.

Beyond thermal stimulation for releasing these captured carriers, near-infrared (980 nm) laser and pressure stimuli can also be employed to release the captured carrier, giving rise to phenomena such as PSL and ML. Consequently, by evaluating the optical properties, such as the intensity of PSL or pressure-induced luminescence, equivalent success in detection has been achieved. In 2023, Wang et al. [145] developed lanthanide ions (Ho^3+^)-doped NaLuF_4_ NCs as a delayed X-ray scintillator. It was developed that X-ray irradiation generates trapping centers in NaLuF_4_: Ho^3+^ NCs, leading to ML upon mechanical stimulation after X-ray exposure (Fig. 9e). Additionally, the ML intensity correlates linearly with the X-ray dose, facilitating the detection of delayed X-ray does radiation. Furthermore, it has been demonstrated that the delayed X-ray information can also be read out by thermal stimulation or 980 nm laser irradiation, endowing the system with multimodal readout capabilities (Fig. 9f) [109]. However, it is important to note that ML or PSL behavior has not yet been fully realized for perovskite scintillator due to their relatively low trap capture efficiency compared to fluorides. Future work should also focus on enhancing trap capture efficiency to improve the readout methods and accuracy of delayed X-ray detection.

### Trade-off between Light Yield and Response Time

The optimization of MHP scintillators necessitates a nuanced balance between enhancing light yield and modulating response time, as strategies targeting one parameter often impose trade-offs on the other. This inherent trade-off arises from the fundamental physical mechanisms governing radiative recombination, carrier dynamics, and ET pathways. For instance, nanostructuring (e.g., QDs or nanoplatelets) and layered structures improve light yield by suppressing non-radiative recombination through quantum confinement, yet their high surface-to-volume ratio introduces surface defects that act as charge traps, which prolong carrier lifetimes by trapping charges [147]. These traps will delay the radiative recombination process, increasing response time. Similarly, STE-based scintillators (such as Cu_3_Cu_2_I_5_) leverage large Stokes shifts (> 200 nm) to minimize self-absorption, achieving light yields exceeding 123,000 Ph MeV⁻^1^. However, the soft lattice and strong exciton-phonon coupling responsible for STE formation also result in broad emission bands and slow recombination kinetics (0.734 μs) [4, 148]. The decay times of STE-dominated materials typically range from micros to millis (Table 2), rendering them unsuitable for applications requiring nanosecond-scale temporal resolution. Doping with activators (e.g., Mn^2^⁺ or lanthanide ions) or constructing ET channels (e.g., MHP-dye composites) effectively reduces self-absorption by decoupling absorption and emission spectra. For instance, Mn^2^⁺-doped BA₂PbBr₄ achieves a light yield of 85,000 Ph MeV⁻^1^ but inherits the millisecond-scale decay time (727 μs) characteristic of Mn^2^⁺’s ^4^T₁ → ⁶A₁ transition [40]. Similarly, ET to organic dyes red-shifts emission but introduces additional recombination pathways governed by the dye’s intrinsic lifetime, which may still lag behind pure MHP systems. Conversely, delayed detection methods (e.g., thermally/mechanically stimulated luminescence) enable high-resolution 3D imaging by extending the temporal window for signal acquisition. Materials like CsCdCl₃:Mn^2^⁺, Zr^4+^ exhibit persistent luminescence for hours post-irradiation [144] facilitating complex geometry imaging. However, these systems inherently prioritize spatial resolution over speed, rendering them incompatible with real-time applications. Overall, the optimization of MHP scintillators constitutes an inherently multidimensional challenge, necessitating careful consideration of application-specific performance requirements. These requirements may include but are not limited to high-speed imaging capabilities, high-sensitivity detection thresholds, or delayed response characteristics in time-resolved detection systems. As such, future work must adopt a strategic approach to parameter optimization that carefully balances these competing demands while addressing the diverse operational requirements of modern X-ray detection technologies.

## Radioluminescent Engineering Strategies

The integration of RL light engineering techniques into X-ray imaging technology, which is crucial for enhancing both resolution and contrast, represents a sophisticated and complex field. It revolves around the efficient manipulation and optimization of light conversion from X-rays to visible signals. Key aspects include the development of stacked techniques, the exploitation of the waveguide effect, and polarized RL; the enhancement of transparency and the innovation of flexible films are summarized as follows.

### Stacked Technique

Traditional techniques rely on single-energy X-ray sources, forming images based on the degree of X-ray absorption by materials. However, this approach lacks the ability to provide information on the chemical composition or density within objects, limiting its accuracy and effectiveness in specific applications. To address this limitation, researchers have developed a stacked technique for multi-energy X-ray imaging (MEXI), which analyzes the attenuation of X-rays at various energy levels within an object [73]. Stacked scintillators combine layers of scintillator with distinct X-ray absorption characteristics and luminescent properties, enabling simultaneous detection across multiple energy spectra.

**Fig. 10:**
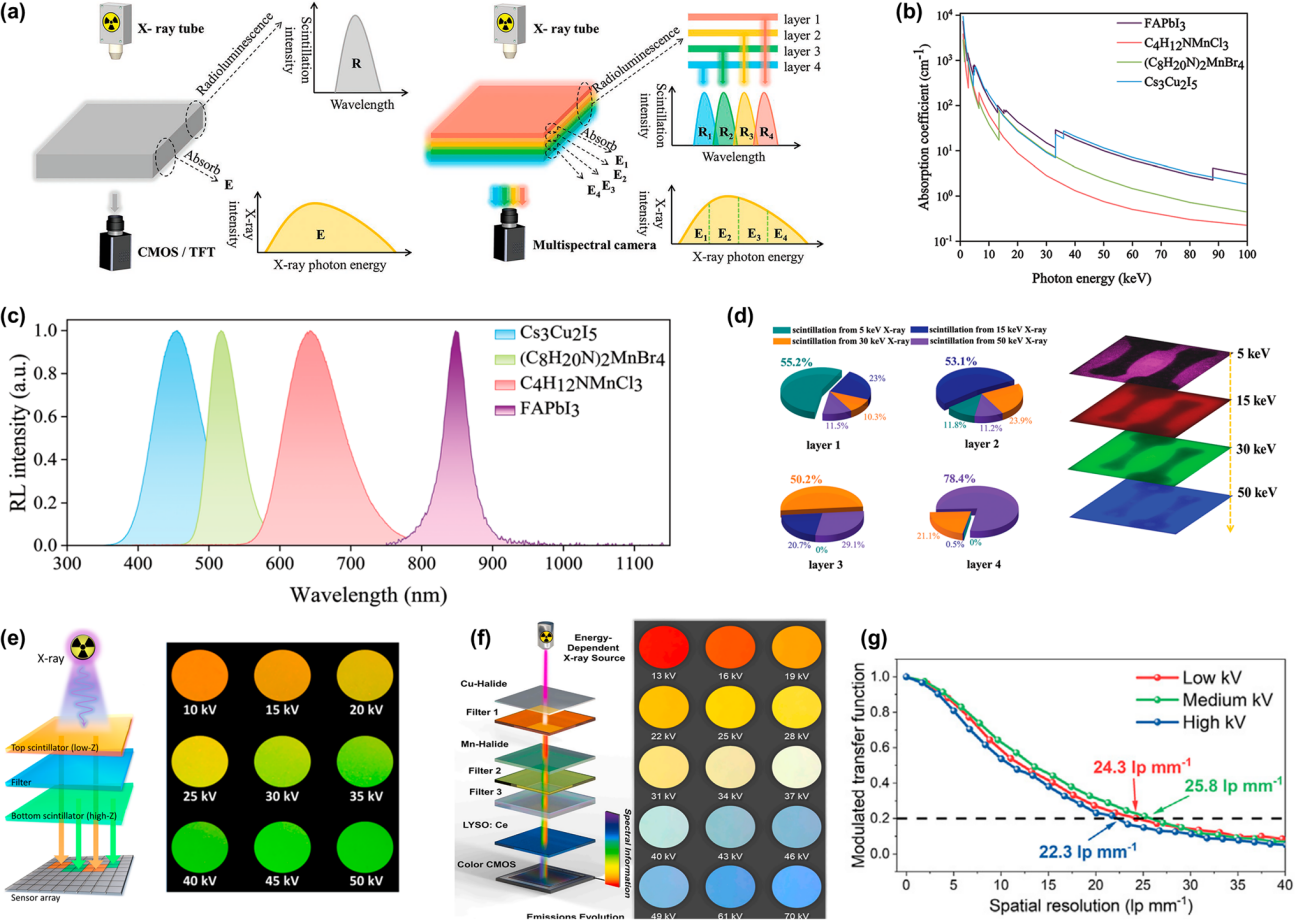
Scintillator performances of stacked MHPs. **a** Schematic of a conventional energy-integrated X-ray imaging system and large-area multi-energy FPXI system based on stacked multilayer scintillators. **b** X-ray absorption coefficients of FAPbI_3_, C_4_H_12_NMnCl_3_, (C_8_H_20_N)_2_MnBr_4_ and Cs_3_Cu_2_I_5_. **c** RL spectra of FAPbI_3_, C_4_H_12_NMnCl_3_, (C_8_H_20_N)_2_MnBr_4_, and Cs_3_Cu_2_I_5_ scintillators. **d** The proportion of the four energy X-rays contributing to the light emitted by each scintillator and the corresponding multi-energy X-ray images at four energy channels at 5, 15, 30, and 50 keV [73]. **e** Schematic of the working principle and the corresponding color evolution of the TFB sandwich structure scintillator [149]. **f** Schematic of the working principle and the corresponding color evolution of the MEXI system featuring a six-layered architecture scintillator. **g** MTF curves of the MEXI system under low-, medium-, and high X-ray tube voltages [93]

In 2022, Yang et al. [73] pioneered a multilayer stacked scintillator for large-area multispectral flat panel X-ray imaging (FPXI) (Fig. 10a). This method overcomes the limitations of traditional energy integration imaging by providing spectral information and enhancing image contrast. Here, the scintillator layers are carefully designed to have different X-ray absorption capabilities and RL wavelengths. The top layer is responsive to low-energy X-rays, emitting long-wavelength lights, while the bottom layer absorbs high-energy X-rays and produces short-wavelength lights (Fig. 10b). A prototype four-layer scintillator (FAPbI_3_/C_4_H_12_NMnCl_3_/(C_8_H_20_N)_2_MnBr_4_/Cs_3_Cu_2_I_5_; Fig. 10c) successfully resolved a "bone muscle" phantom, demonstrating energy-dependent feature discrimination (Fig. 10d). Subsequently, Mohammed et al. [149] advanced the above strategy by devising a top filter bottom (TFB) sandwich architecture (Fig. 10e), incorporating quartz as a strategic filter. The layer filters strategically remove the energy range where the absorption tails of the upper and lower scintillators intersect, thereby ensuring a clearer distinction between their emission signatures. Illustratively, X-ray images obtained at varying voltages demonstrate the efficacy of the TFB design, where each layer effectively discriminates between low- and high-energy radiation through their characteristic luminescence, enabling the differentiation of material composition and achieving a resolution of approximately 18 lp mm^−1^. Xiaoming Li et al. [150] further refined a TFB structure through meticulous modulation of scintillators, an innovation that not only elevates the performance of scintillating materials but also powerfully demonstrates the broad applicability of this technique. Recent advances by Mohammed et al. extended this paradigm to a six-layer MEXI system with interlayer optical filters (Fig. 10f) [93]. By strategically implementing interlayer filters, they ensure that the X-ray beam is tailored to pass only specific visible wavelengths to the respective scintillators, thereby maintaining non-overlapping RL. This critical design element allows each scintillator layer to capture unique, spectral signatures of the imaged object, enhancing both the clarity and precision of the data and achieving 22 lp mm⁻^1^ resolution across low-to-high X-ray doses (Fig. 10g). However, increasing the number of layers can lead to mutual excitation between layers, which may affect the accurate discrimination of X-ray energy. To mitigate this, Yang et al. [151] proposed a strategy for a multi-energy X-ray detector based on quantum-cutting perovskite scintillators. By using Yb^3^⁺-doped CsPbCl_3_ NCs in the top layer, this design induced a 570 nm absorption–emission shift and > 100% photoluminescence PLQY, suppressing crosstalk while pairing with STE-based scintillators (CsAgCl₂/Cs₃Cu₂I₅) in lower layers. The resultant non-overlapping RL spectra significantly improved material discrimination, particularly for density-similar substances.

While stacked scintillator architectures achieve multi-energy discrimination through material stratification, parallel advancements in direct detection paradigms offer distinct approaches. Tang et al. [152] developed a vertical matrix perovskite detector with electrodes aligned parallel to the X-ray beam. Low-energy photons deposited energy in shallow layers, while high-energy photons penetrated deeper regions. Using MAPbBr_3_ SCs with five vertically stacked electrodes and machine learning-derived conversion matrices, the system achieved single-shot density gradation imaging, distinguishing soft tissues from high-density composites with 0.81 × 10^5^ μC Gy_air_⁻^1^ cm⁻^3^ sensitivity and 0.82 μGy_air_ s⁻^1^ detection limits. Additionally, Shabbir et al. [153] developed a solution-processed Cs₀.₁FA₀.₉PbI₃ perovskite thin-film detector for broadband multi-energy X-ray detection. The n-i-p diode architecture (SnO_2_/Cs_0.1_FA_0.9_PbI_3_/Spiro-OMeTAD/Au) exhibited 6 × 10^4^ µC Gy⁻^1^ cm⁻^2^ sensitivity in hard X-rays (> 10 keV) with < 5 ms response times, and 5 × 10^3^ µC Gy⁻^1^ cm⁻^2^ sensitivity in soft X-rays (0.1–10 keV) with 60 dB SNR at 1.2 keV.

### Waveguide Effect

**Fig. 11:**
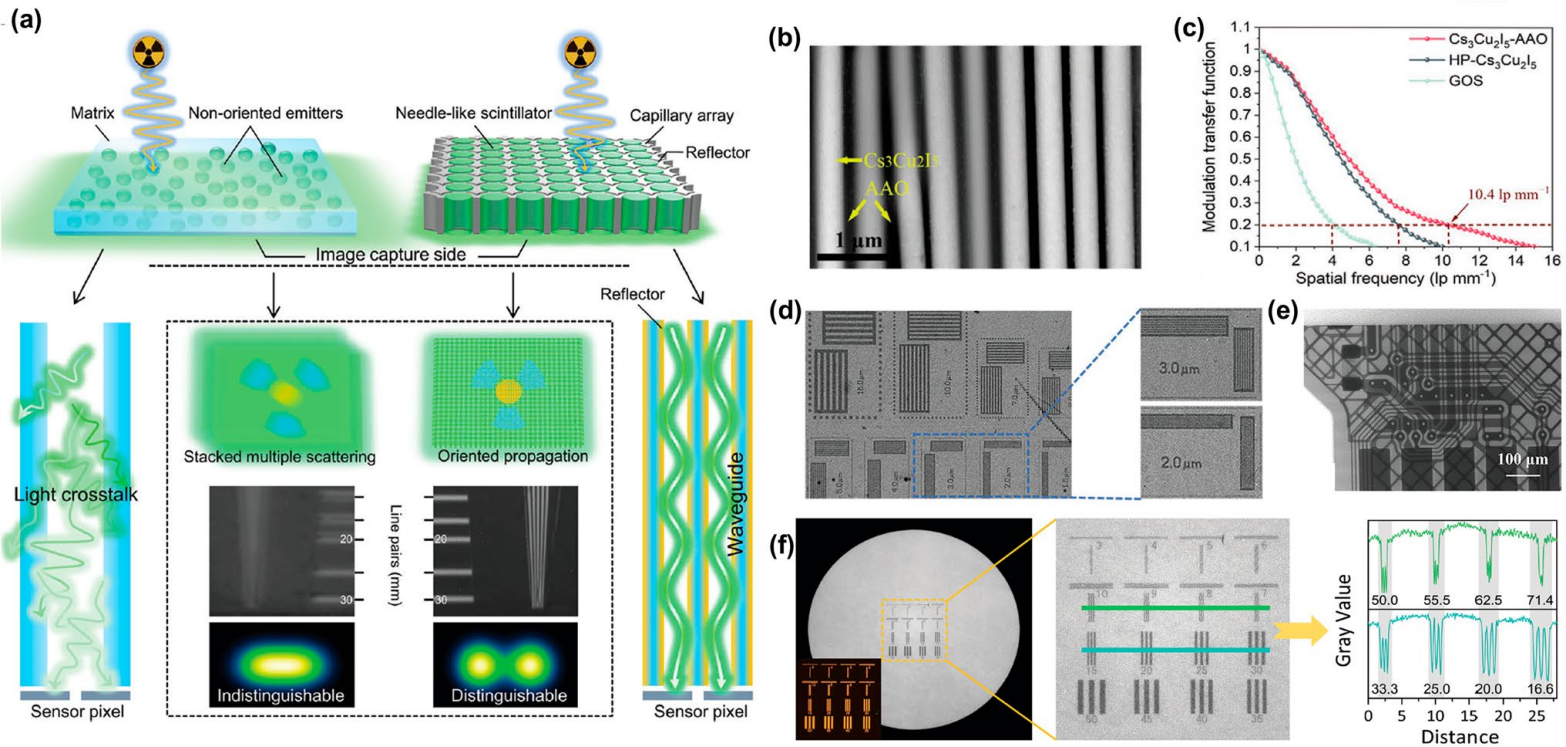
Scintillator performances of waveguided MHPs. **a** Light propagation mechanisms and results of conventional nonstructured scintillators and structured capillary needle-like array scintillators in X-ray imaging [154]. **b** Cross-sectional SEM image of Cs_3_Cu_2_I_5_-AAO scintillator. **c** The MTF values of Cs_3_Cu_2_I_5_-AAO, HP-Cs_3_Cu_2_I_5_, and GOS [155]. **d** Spatial resolution of the pixelated CsPbBr_3_-AAO arrays scintillation. **e** X-ray imaging of memory card with the pixelated CsPbBr_3_-AAO arrays [156]. **f** X-ray image of a microresolution chart. The right one is the gray value profiles along the cyan and green lines extracted from the X-ray image of the microresolution chart [154]

Waveguide effect, a physical phenomenon that controls the direction of photon propagation, has been proved to play a significant role in enhancing imaging resolution. By restricting lateral photon diffusion, it boosts the signal collection efficiency within the imaging system, thereby markedly improving resolution. Based on the principle of total internal reflection, where light incident at an angle greater than the critical angle on the interface between two media is reflected entirely within the higher refractive index medium, the waveguide effect enables light to propagate along interfaces without leakage (Fig. 11a). In X-ray scintillators, the design of nanostructures or microstructures with specific orientations facilitates the effective transmission and confinement of photons, thereby reducing lateral spread and enhancing imaging resolution.

In 2003, Eijk [157] laid the groundwork by suggesting the use of pixelated scintillator arrays embedded within a tailored matrix to hinder inter-pixel photon leakage, aiming to enhance spatial resolution. Building on this insight, Tang et al. [155] achieved a significant breakthrough by fabricating a Cs_3_Cu_2_I_5_ array scintillator. They ingeniously integrated Cs_3_Cu_2_I_5_ into an anodic aluminum oxide (AAO) matrix via a facile hot pressing process (Fig. 11b). The nanostructured AAO matrix is pivotal in constraining lateral light diffusion, thereby drastically reducing photon crossover between pixels. Notably, the fabricated Cs_3_Cu_2_I_5_-AAO scintillator exhibits superior spatial resolution (10.4 lp mm^−1^) (Fig. 11c). Yang et al. [158] further enhanced the resolution of the Cs_3_Cu_2_I_5_ scintillator by optimizing the Cs_3_Cu_2_I_5_-AAO fabrication process. This approach enables precise control over the length of the Cs_3_Cu_2_I_5_ NWs by adjusting the pore depth of the AAO matrix, offering tunability in size. As a result, they achieved high-resolution imaging with a spatial resolution of 20 lp mm^−1^. Zhang et al. [156] prepared pixelated CsPbBr_3_-AAO array scintillation screens by adsorbing synthesized CsPbBr_3_ NCs into the array voids of AAO via negative pressure filling. This method achieved an ultrahigh spatial resolution of 211 lp mm^−1^(Fig. 11d, e), further demonstrating the versatility of the AAO template approach for different pixelated MHPs scintillators. In addition to the aforementioned method of using an AAO template for alignment, Mohammed recently proposed the fabrication of a pixelated scintillator array through the integration of the low-temperature melting of manganese halide compounds within an aluminum-clad capillary template [154]. The as-obtained pixelated scintillator achieves remarkable spatial resolutions of 60.8 and 51.7 lp mm^−1^ at a MTF of 0.2, for 0.5 and 1 mm thicknesses, respectively (Fig. 11f).

Despite numerous accomplishments, the above processes often entail heating or melting, which can potentially harm the delicate perovskite crystal structure, subsequently compromising the scintillator's performance. Consequently, exploring alternative methods to safeguard the crystal integrity remains a pressing pursuit. Recently, Wang et al. [85] devised an innovative one-step in situ strategy for the growth of Cs_5_Cu_3_Cl_6_I_2_ QDs within AAO matrix, yielding a scintillator in the form of a Cs_5_Cu_3_Cl_6_I_2_@AAO array scintillator. This approach has led to the development of a pixelated scintillator array and boasts a spatial resolution of approximately 20 lp mm⁻^1^ and a remarkable detection sensitivity of 0.152 Gy s⁻^1^.

### Chiral Circularly Polarized Luminescence (CPL)

**Fig. 12:**
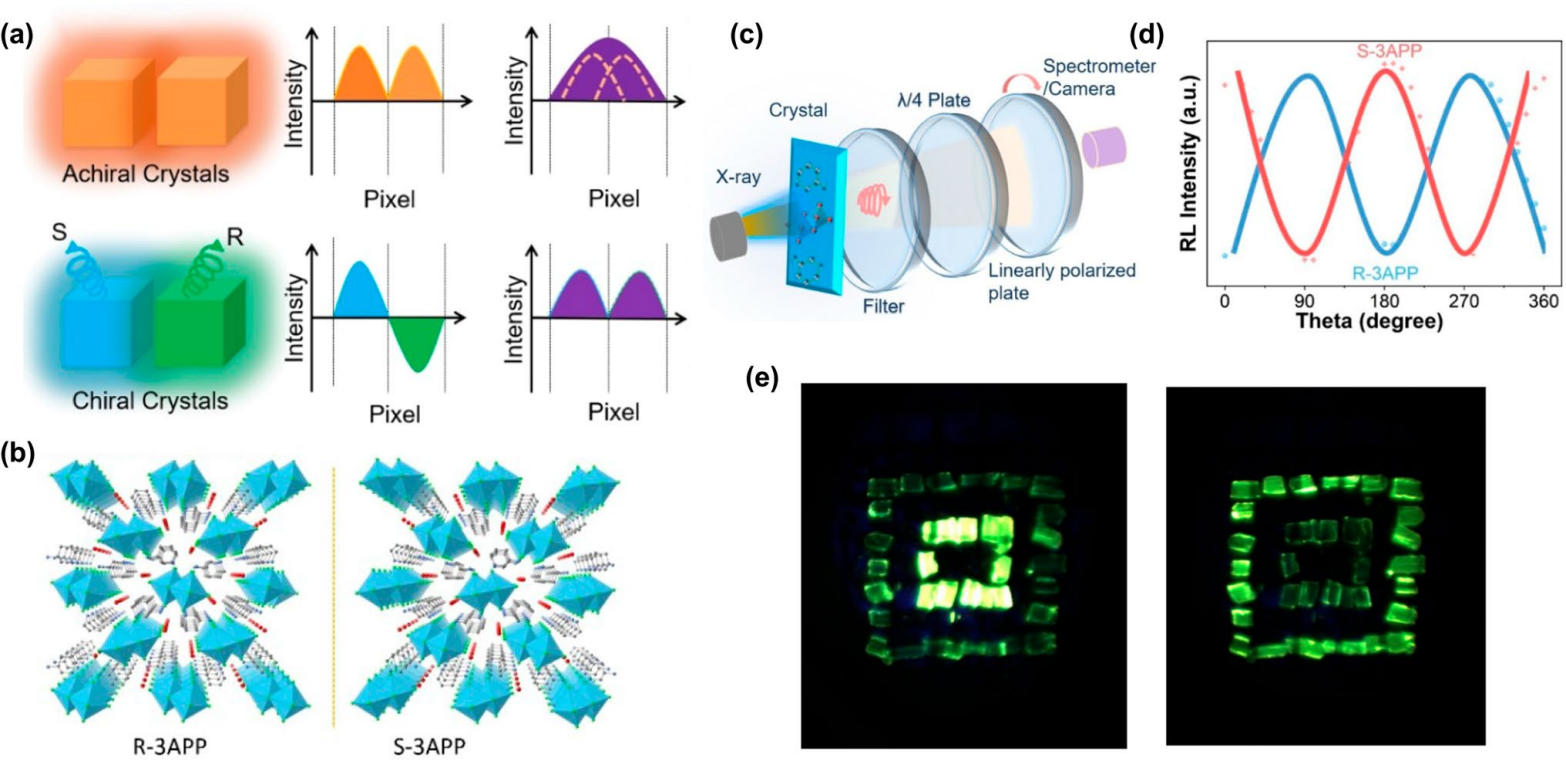
Scintillator performances of CPL MHPs. **a** Conceptual illustration of the minimization of optical crosstalk by CPRL. **b** The crystal structures of R-3APP and S-3APP. **c** Schematic illustration of the experimental setup for verifying the CPRL measurement. **d** The polarization-dependent RL of chiral S-3APP and R-3APP. **e** The left-handed and right-handed RL emission crystals show bright and dark changes when rotating the linearly polarized plate [159]

CPL materials exhibit differential intensities between left- and right-handed circularly polarized light, a trait pivotal for enhancing the resolution in imaging technologies (Fig. 12a). Conventional imaging techniques often suffer from inter-pixel crosstalk due to the isotropic propagation of light, which hampers significant improvements in resolution. The integration of CPL materials introduces a novel paradigm by generating light of a specific chirality that can be precisely controlled, thereby mitigating crosstalk and augmenting image contrast and resolution.

In recent years, advancements in organic–inorganic hybrid halide materials have propelled CPL research forward, with these materials boasting high PLQY and substantial circular polarization efficiencies (glum). Moreover, they have shown immense promise in the realm of X-ray detection and imaging. In 2022, Li et al. [159] presented a pioneering strategy to enhance X-ray imaging clarity by employing chiral MHP scintillators that emit circularly polarized radioluminescence (CPRL). These scintillators, exemplified by (R-3AP)PbBr_3_Cl·H_2_O (R-3APP) and (S-3AP)PbBr_3_Cl·H_2_O (S-3APP) (Fig. 12b), showcase the distinctive attribute of circular polarization in its RL response to X-ray irradiation. To demonstrate the CPRL enhanced the imaging quality, they constructed a simple matrix that showcased the optical differences between traditional and chiral scintillators by using a homemade polarized light collecting system (Fig. 12c). Notably, the RL intensity of S-3APP and R-3APP displays a significant polarization dependence (Fig. 12d), suggesting that the RL is intrinsically polarized with asymmetric oscillations along the propagation direction. Finally, they assembled a series of chiral scintillators by alternating left-handed and right-handed crystals. Under X-ray exposure, this assembly exhibited a distinct contrast resolution, indicating a unique light propagation pattern for each crystal (Fig. 12e). This work successful demonstrated of the concept of chiral scintillators herein opens an avenue to controlling the RL propagation, potentially minimizing the optical crosstalk that has plagued the scintillator field for a long time.

Building on this foundation, Wang et al. [160] introduced chiral organic–inorganic hybrid Mn^2+^ halide clusters with exceptional PLQY and CPL characteristics. These clusters, designated (R/S-3-aminopyrrolidine dihydrochloride)_6_(Mn_3_Cl_12_)(Cl)_6_ (R/S-1) and (R/S-1,2-diaminopropane dihydrochloride)_3_(Mn_3_Cl_12_)(R/S-2), were instrumental in advancing X-ray imaging applications, achieving high detection sensitivity and resolution. They also demonstrated that the directional arrangement of chiral ligands within these clusters was key to amplifying the CPL effect, underscoring the significance of structural design in enhancing material performance. Alexander et al. [161] synthesized chiral Mn^2+^ bromide hybrids that emit bright green CPL with near-unity PLQY and a high dissymmetry factor. These hybrids, combining [MnBr_4_]^2−^ anions with N/O–H bond-free cations, showcased excellent optical selectivity and a strong linear response to X-ray dose rate, positioning them as cost-effective candidates for CPL X-ray imaging.

In addition to indirect X-ray detection, Luo et al. [162] further expanded the horizons of self-powered direct X-ray detection with the development of a 2D lead-free double perovskite, R-MPA)_4_AgBiI_8_. This perovskite, which crystallizes in a polar space group at room temperature based on the introduction of chiral cations, results in a polar bulk photovoltaic effect under X-ray excitation, with an open-circuit voltage of 0.36 V. Without any external bias, the sensitivity and detection limit of the X-ray detection are measured to be of 46.3 μC Gy^−1^ cm^−2^ and 85 nGy_air_ s^−1^, respectively. Furthermore, under an applied bias of 50 V, the sensitivity is successfully enhanced to 946 μC Gy^−1^ cm^−2^. This work represents a landmark in the advancement of "green" radiation detectors based on the CPL effect.

### Enhancing Transparency

MHP SC scintillators have important application prospects in X-ray imaging, but due to their inherent impurity defects and the presence of polycrystalline interfaces, their transparency is low, which affects imaging resolution. Impurity defects are one of the common problems in perovskite materials, and their presence can scatter light, reducing the transparency of the material. Additionally, the presence of polycrystalline interfaces is also an important factor that affects the transparency of perovskite materials, as the interfaces can scatter light, reducing the transparency of the material. As a consequence, the resolution of imaging systems is compromised, with scattered light blurring the definition of image edges, reducing contrast, and thereby compromising the fine detail and sharpness of the final image [163, 164]. Therefore, improving the transparency of MHP SC scintillators is the key to improving the X-ray imaging resolution.

**Fig. 13:**
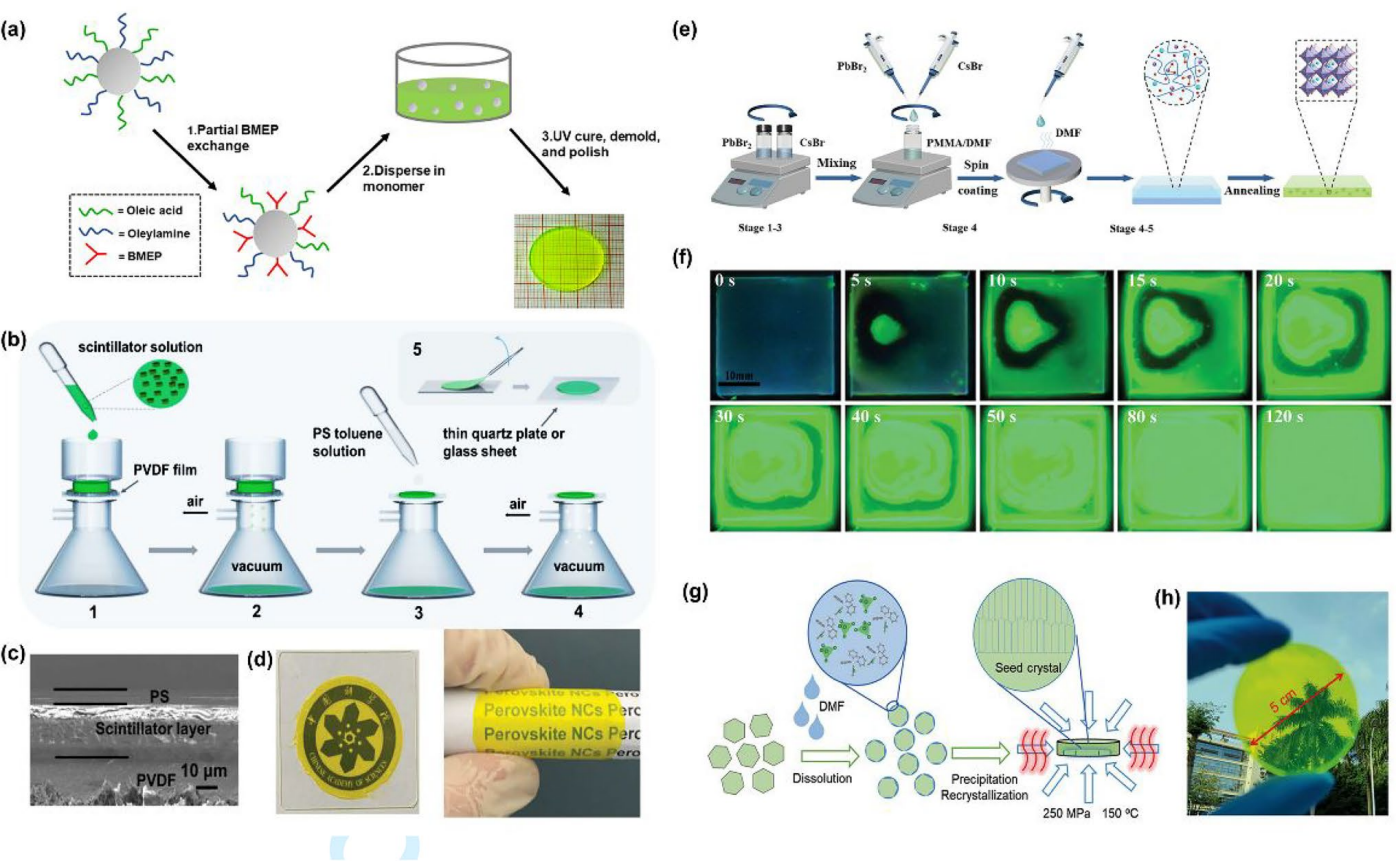
Development of the transparent MHP scintillator. **a** Schematic diagram of the surface modification and polymerization process for preparing the CsPbBr_3_/PBMA nanocomposites [166]. **b** Scheme of the scintillator film synthesis procedure by the suction filtration method and the corresponding **c** SEM and **d** optical images [169]. **e** Schematic diagram of the in situ growth of CsPbBr_3_-based polymer–ceramics. **f** Photographs of the one-step in situ growth of CsPbBr_3_ QDs from transparency matrix under UV illumination [38]. **g** Fabrication process of TPP_2_MnBr_4_ ceramic scintillator via the SCS process. **h** Photographs of as-obtained TPP_2_MnBr_4_ ceramic scintillator [170]

Recently, innovative research has suggested an approach to tackle the transparency issues by using the perovskite QDs as the scintillator. The key lies in the unique properties of these QDs, particularly their quantum size effect, which suppresses light scattering based on the Rayleigh scattering law. [165]. For instance, Ouyang et al. [166] developed a transparency X-ray scintillator by directly dispersing surface-modifying CsPbBr_3_ NCs in a transparent polybutylmethacrylate (PBMA) matrix (Fig. 13a), achieving 99.2% PLQY, 70% visible transparency. This solution-processed scintillator screen exhibits high-contrast visualization at ultralow doses (4.6 μGy_air_ s^−1^). This universal dispersion approach demonstrates material-agnostic applicability, where transparent scintillators with tailored properties can be fabricated through rational selection of perovskite compositions (e.g., CsPbX_3_, Cs_3_Cu_2_I_5_) or transparent matrices (e.g., epoxy, PMMA, PVDF), establishing a versatile paradigm for radiation scintillators [55, 167, 168]. However, the direct integration of perovskite QDs into the transparent matrix is challenged by their propensity to aggregate during composite fabrication. Such aggregation can cause inconsistencies in light emission and scattering, leading to non-uniform light distribution within the film.

To overcome these limitations, Gu et al. [169] devised a vacuum-assisted "suction filtration" strategy to fabricate 30-μm-thick CsPbBr_3_ NC films (Fig. 13b). This involved filtering perovskite NC solutions through PVDF membranes to form dense, uniform scintillator layers, followed by polystyrene (PS) gap-filling and transfer onto quartz substrates (Fig. 13c), with film thickness precisely tunable by adjusting NC solution concentration. This film not only has high density but also exhibits excellent transparency (Fig. 13d), with transparency exceeding 80% at a thickness of 30 µm. Using this ultrathin transparent perovskite scintillator film, the researchers successfully achieved a spatial resolution of approximately 862 nm, which is the highest resolution recorded to date for perovskite scintillators used in X-ray microscopy imaging. Additionally, Xu et al. [22, 38] developed a chemical anchoring strategy through one-step in situ growth of CsPbBr_3_ QDs within transparent matrices. Here, CsBr/PbBr_2_ precursors in DMF were mixed with PMMA colloids, forming intermediate clusters, and then polymer pre-curing and DMF evaporation triggered heterogeneous nucleation of perovskite NCs at these clusters, yielding a MHP film with uniformly dispersed perovskite NCs (Fig. 13e, f). This approach eliminates the light scattering caused by traditional physical blending, resulting in a homogeneous composite with high spatial resolution of 12.5 lp mm^−1^. The universality of this methodology is further validated by its successful extension to copper-based perovskite Cs_3_Cu_2_I_5_ [171]. The resulting scintillator achieves a record—high resolution of 14.3 lp mm^−1^ while maintaining comparable transparency. The perovskite QDs can also be grown from a transparent glassy network [22] and then yielded scintillators with an impressive spatial resolution of about 16.8 lp mm^−1^ and a rapid decay time of 27 ns. A crucial innovation in the above works lies in the nucleation inhibition strategy that suppresses crystal agglomeration and Ostwald ripening, ensuring uniform QD distribution. However, the pursuit of transparency introduces an inherent trade-off: The matrix materials, while enhancing light transmission, exhibit parasitic X-ray absorption. This energy loss mechanism reduces the scintillation light yield compared to perovskite single crystal.

To address the light yield transparency trade-off, transparent polycrystalline ceramics have emerged as a promising solution. For instance, Xia et al. [170] introduced an innovative technique, seed crystal-induced cold sintering (SCS) (Fig. 13g), for the fabrication of transparent MHP ceramic scintillators (Fig. 13h). This method yielded large-area, < 001 > -textured TPP_2_MnBr_4_ ceramics, and the textured poled samples atop surface direction exhibited enhanced linear transmittance for the visible light spectrum compared to the one at lateral surface direction. Finally, the as-obtained TPP_2_MnBr_4_ ceramics showcase exceptional performance with a light yield of approximately 78,000 ± 2,000 Ph MeV^−1^. The scintillators demonstrated a remarkable energy resolution of 17% for high-energy γ-rays (662 keV) and a high-resolution X-ray imaging of 15.7 lp mm^−1^. Building on these advancements, Xia et al. [172] further developed a melt-quenching method using a stoichiometric mixture of ethyltriphenylphosphonium bromide (ETPBr) and MnBr₂ as raw materials at a relatively low temperature of 200 °C to fabricate (ETP)₂MnBr₄-based polycrystalline ceramics. The resulting scintillator demonstrated high transparency (over 80% in the 500–800 nm range), a high light yield of ≈35,000 ± 2,000 Ph per MeV, a low detection limit of 10^3^nGy s⁻^1^, and a competitive spatial resolution of 13.4 lp mm⁻^1^ for X-ray imaging.

Overall, the quest to enhance transparency in MHP scintillators has driven significant innovations, from embedding QDs in transparent matrices to developing advanced polycrystalline ceramics. Each approach offers unique advantages, such as improved spatial resolution and light yield, while addressing critical challenges like light scattering and parasitic absorption. These advancements collectively pave the way for high-performance scintillators that can meet the stringent demands of modern X-ray imaging technologies, promising clearer, more detailed images with reduced radiation doses.

### Curved Scintillator Imaging

**Fig. 14:**
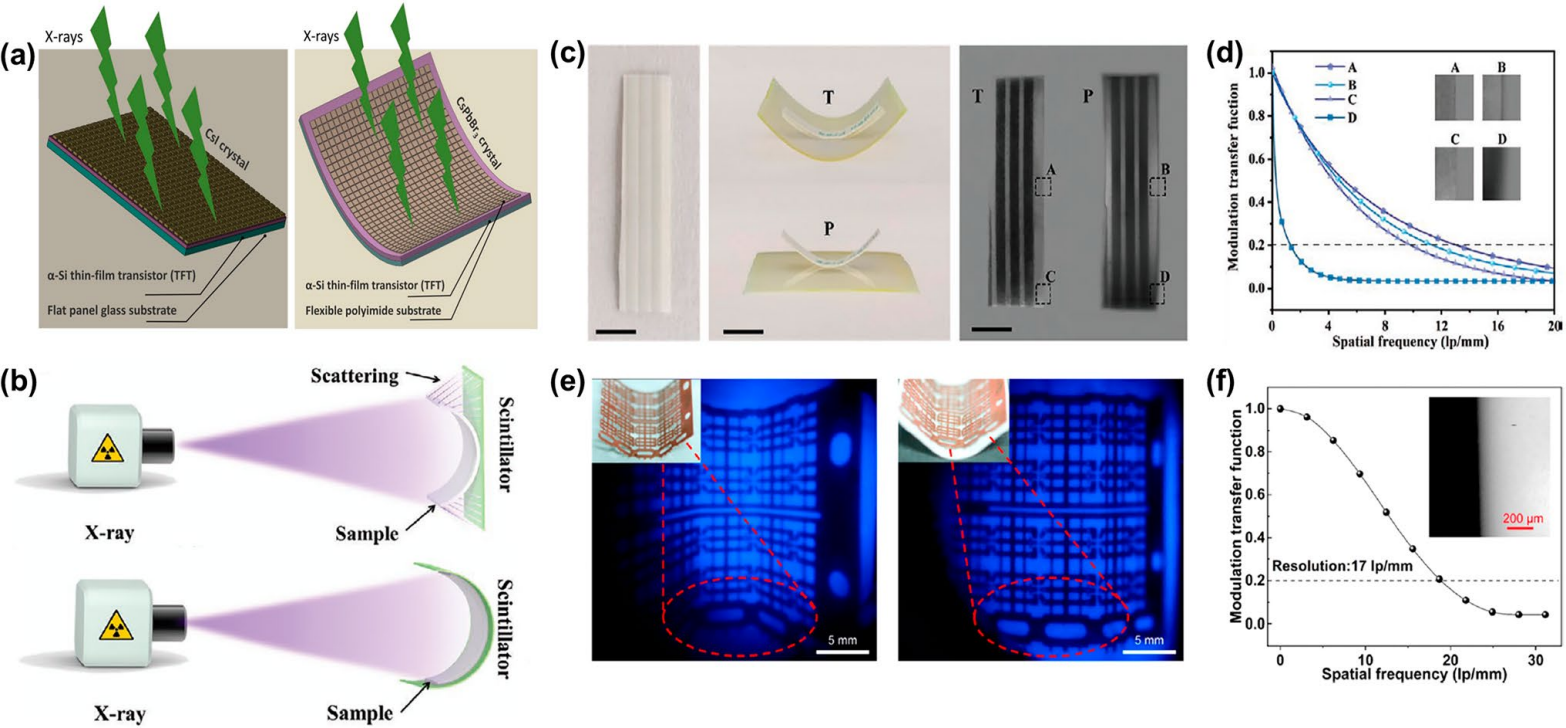
Scintillator performances of curved MHPs. **a** Detector structure sketches: fat panel glass substrate and a flexible polyimide substrate [160]. **b** The diagram of the X-ray imaging under flat and bending states. **c** The difference of the corresponding indirect X-ray imaging ways, and the corresponding X-ray images of a bend target with the attached and projection imaging, respectively, and **d** their corresponding MTF of the specified location (A, B, C, and D) of the target [38]. **e** X-ray images of flexible copper grid taken by flexible Cs_2_Cu_2_I_5_ film under flat and bending states. **f** MTF of the flexible Cs_2_Cu_2_I_5_ film [174]

Traditional imaging methodologies, predominantly grounded in planar imaging, have proved their efficacy in a wide array of applications. However, when it comes to non-destructive testing of intricate, curved, or anisotropic structures, the limitations of planar imaging become apparent, particularly at the edges. In planar imaging, the quality of the image decreases from the center to the edge due to the angle at which photons hit the outer pixels, a phenomenon known as vignetting. Typically, a series of optical lenses are employed to combat vignetting, but this approach results in a bulkier and more complex optical system. In response to these challenges, the development of curved imaging technology based on flexible scintillators has emerged as a promising solution. Curved scintillator imaging technology simplifies the imaging system and mitigates the vignetting effect at the edges, thereby excelling in non-destructive testing of complex structures. Gelinck et al. [173] proposed a concept of flexible imaging technology with a high-resolution curved image sensor made directly on a thin plastic substrate using organic photodetectors (OPD). As a proof of concept, the curved digital detector, combined with a flexible scintillator, demonstrates more uniform image quality. Moreover, Wang et al. [160] utilized the Monte Carlo method to elucidate the imaging performance of flexible CsPbBr_3_ detectors, theoretically substantiating their superior performance over conventional NaI and CsI-based flat panel detectors in terms of resolution, peak-to-total ratio, and total efficiency (Fig. 14a). However, despite the promising theoretical framework, there has been a notable absence of tangible device or experimental progress in this area.

Until 2021, Xu et al. [38] introduced a novel approach, fabricating flexible MHP QDs-based scintillator materials through in situ crystallization within polymers. The obtained polymer–ceramic film showcases remarkable flexibility, enduring bending to 90° without fracture, enabling X-ray detection on curved surfaces (Fig. 14b), as opposed to conventional projection imaging. This bypasses the scattering issue inherent in projection imaging for non-planar targets due to the gap between the target and scintillator. A comparison of imaging qualities using the attached (T) versus projection (P) methods (Fig. 14c), using a flexible circuit board, shows marked differences, particularly at locations A, B, C, and D. The MTF analysis highlights a substantial resolution disparity, with C achieving 9.5 lp mm^−1^ and D at 1.98 lp mm^−1^, underscoring the superior spatial resolution performance of curved scintillator imaging in non-planar X-ray detection (Fig. 14d). Subsequently, we achieved in situ crystallization of Cs_3_Cu_2_I_5_ perovskite within a PVDF/PMMA polymer matrix and on cellulose paper, fabricating copper-based perovskite flexible scintillators [171]. This enabled successful high-resolution imaging, thereby further affirming the significance of flexible scintillators for the detection of non-planar objects, highlighting their potential in versatile imaging applications. Additionally, Zhang et al. [175] demonstrated that in situ fabricated (TBA)CuX_2_/PVDF flexible films were efficient scintillation screens for flexible X-ray imaging, with the spatial resolution of 166 μm achieved.

In addition to the in situ growth of MHP from a flexible matrix to form a flexible scintillator, other methods, such as directly combining MHP with a polymer matrix, have been explored. However, these methods often suffer from the issue of particle aggregation. To address this challenge, Omar F. Mohammed et al. [174] proposed a solution involving the addition of ethylacetate (EA). By incorporating EA, a highly uniform flexible Cs_2_Cu_2_I_5_ film can be fabricated through either the spin-coating or blade-coating method. The presence of EA effectively inhibits the aggregation of Cs_2_Cu_2_I_5_ particles, resulting in a highly uniform flexible scintillator film that exhibits nearly 100% PLQYs (Fig. 14e). These films have demonstrated very promising scintillation properties, including a low detection limit of 48.6 nGy_air_ s^−1^ and an X-ray imaging resolution of 17 lp mm^−1^ (Fig. 14f). Moreover, Wu et al. [176] proposed that the (BTPP)_2_MnCl_4_@PDMS flexible scintillation screens achieve a high spatial resolution of 14.1 lp mm^−1^ and realize high-quality imaging results of non-planar objects. Xiao et al. reported a novel 2D RP phase Cs_2_CdCl_4_: Mn^2+^, with the cooperation of PDMS matrix, which shows a high light yield up to 88,138 Ph MeV^−1^ and a high spatial resolution of 16.1 lp mm^−1^. Overall, the development of flexible scintillator materials has revolutionized X-ray imaging, particularly for non-planar targets, by offering high light yields and exceptional spatial resolutions.

## Conclusions and Perspectives

**Fig. 15:**
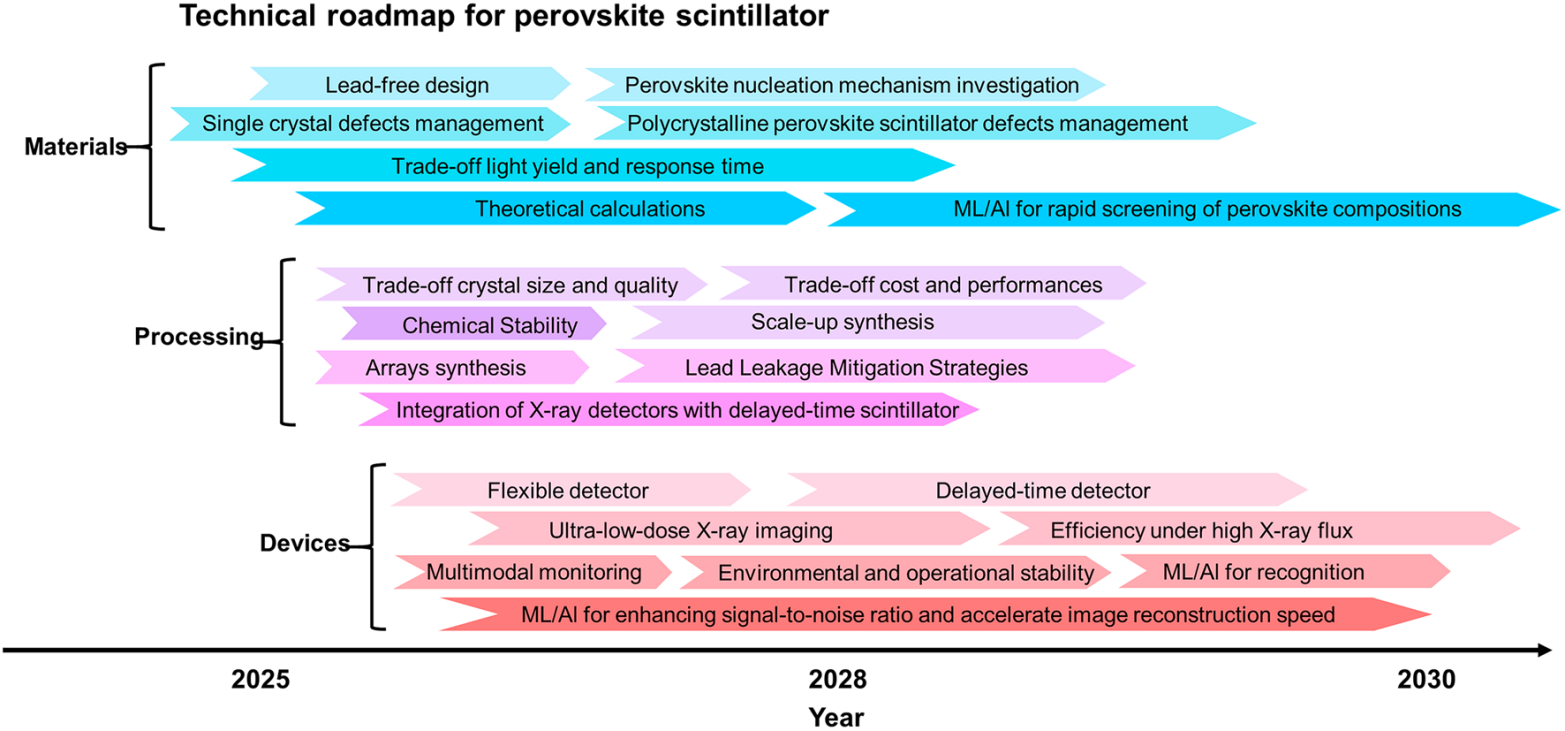
Technical roadmap of perovskite scintillators over the next five years. ML, mechanical learning; Al, artificial intelligence

To sum up, MHP scintillators have emerged as a transformative class of materials for high-resolution X-ray detection, distinguished by their exceptional X-ray attenuation capabilities, near-unity luminescence efficiency, tunable emission spectra, and ultrafast decay kinetics. These intrinsic advantages, coupled with their structural versatility and cost-effective processability, have driven unprecedented advancements in radiation detection technologies. Diverging from prior reviews, this work provides a systematic framework for understanding and optimizing MHP scintillators through two complementary lenses: intrinsic property modulation and RL engineering strategies. First, we elucidate the fundamental luminescence mechanisms governing MHP scintillators—excitonic recombination, STE emission, TADF, and LPRL—and correlate these processes with material design principles. Subsequently, the paper meticulously reviews strategies to boost the light yield of MHP scintillators, such as employing confinement effects, improving crystalline, and reducing self-absorption. Concurrently, response time modulation is addressed, highlighting the critical balance between rapid decay (e.g., nanosecond decay time for real-time angiography) and delayed luminescence (e.g., trap-mediated storage for 3D tomographic imaging). Beyond intrinsic properties, we pioneer discussions on innovative light management architectures, such as stacked scintillators for multi-energy spectral discrimination, pixelated waveguide arrays to suppress optical crosstalk, and CPRL for contrast-enhanced imaging. The integration of flexible MHP composites further expands their applicability to curved surfaces and complex geometries. However, numerous critical challenges remain unresolved for practical implementation, following the technical roadmap presented there (Fig. 15); we discuss the research challenges that must be resolved to enable successful industrial application and identify some technologies that need to developed to address these challenges.

1. The pursuit of practical applications of MHP scintillators demands urgent resolution of environmental concerns, as lead-based systems, despite their superior light yield and ultrafast decay times—pose significant ecological risks due to inherent lead toxicity. Recent advances in lead-free alternatives, such as Mn^2^⁺/Cu⁺-activated perovskites (e.g., Cs₃Cu₂I₅, Rb₂CuBr₃) [4, 158], demonstrate promising ultrahigh light yields, yet their decay times generally lag behind lead-based counterparts due to distinct recombination mechanisms: Strong spin–orbit coupling in lead-based MHPs enables fast radiative transitions, whereas lead-free systems predominantly rely on STE emission with inherently prolonged lifetimes. This fundamental trade-off among light yield, toxicity mitigation, and temporal response necessitates application-specific optimization. For instance, medical CT imaging prioritizes sub-10 ns decay and high resolution, while security screening may tolerate slower kinetics (> 50 ns) for enhanced environmental safety. Therefore, future research must implement a holistic strategy for parameter optimization that systematically reconciles competing performance metrics while addressing the multifaceted operational requirements of next-generation X-ray detectors across diverse applications.
2. Suppressing defect formation is critical for optimizing the performance of MHP SCs and NCs. However, achieving efficient defect engineering remains a significant challenge. This stems from the intrinsically low formation energy and uncontrolled nucleation kinetics during solution-based synthesis of MHPs, which promote the formation of structural defects such as lead vacancies, halide interstitials, and grain boundaries. These defects result in high defect densities in synthesized scintillators, severely degrading key performance metrics, including light yield, response speed, and environmental stability. Studying the nucleation of MHP, including the critical nucleation pathways and pinpoint defect origins (e.g., twinning, dislocations), can guiding rational control over scintillator fabrication. Therefore, further work should also be focused on the using some advanced technique, such as liquid-cell in situ TEM or SEM technique, for monitoring the detailed nucleation process of MHP. Complementing experimental efforts, machine learning and artificial intelligence, driven material design, offer transformative potential. Future research should prioritize the integration of machine learning and AI-driven approaches, specifically by combining perovskite crystal databases (e.g., Materials Project) with graph neural networks (GNNs). This methodology can systematically predict perovskite compositions that optimize both formation energy and defect tolerance metrics, ultimately enabling accelerated design of high-performance scintillators with tailored functional properties.
3. Current efforts to enhance MHP through above-mentioned chemical engineering or light management methods primarily focus on the preparation of films, MCs or NCs. For films, the presence of numerous crystal interfaces and defects at these interfaces can lead to reduced light yield and increased light scattering losses. For MCs or NCs, researchers mainly use a method of combining them with polymers to prepare so-called uniform films for scintillator screens. However, MCs can cause significant aggregation issues and light scattering losses, while NCs, due to their high surface area, have enormous surface defects that lead to a noticeable reduction in light yield. More importantly, when combined with polymers, the absorption of X-rays or other high-energy rays (such as α or β rays) by the polymers can also result in the absorption of a portion of the radiation energy, further reducing the light yield. These size limitations not only affect the optical performance of MHP materials but also limit their efficiency and reliability in practical applications. Therefore, developing new preparation methods and technologies to simultaneously reduce interface grains, minimize light scattering losses, and enhance light yield is a crucial direction for current research. Additionally, the preparation of MHP SCs can effectively address the issues mentioned above. However, current strategies for enhancing MHP SCs X-ray behaviors are still limited. Future research should focus on optimizing crystal growth protocols (e.g., Bridgman methods) and hybrid architectures (e.g., perovskite-polymer/CG composites) to enhance light yield and suppress scattering.
4. The operational stability of MHP scintillators faces critical challenges from environmental stressors (moisture/heat) and irradiation-induced degradation. While hygroscopicity-driven defect proliferation remains a primary failure mechanism, current research primarily focuses on encapsulation strategies using polymer matrices (e.g., PMMA/PVDF) or in situ synthesis of MHPs NCs to enhance environmental resistance. However, these approaches predominantly assess PL stability while critically overlooking radiation tolerance under high-dose X-ray exposure. A fundamental limitation arises from radiation-polymer interactions: High-energy photons (X/β-rays) induce bond cleavage in organic matrices, generating free radicals that accelerate material degradation. Recent advancements like in situ growth of MHPs QDs in glass networks (MHPs CG) address polymer limitations but introduce new vulnerabilities—radiation-induced structural disordering in glass compromises long-term imaging reliability. Therefore, future efforts must concurrently pursue two pathways: (i) radiation-hardened encapsulation systems resistant to cumulative radiation damage and (ii) development of intrinsically stable perovskite architectures through defect engineering and composition optimization, with mandatory validation under high radiation doses irradiation.
5. MHP scintillators' delayed X-ray detection capability enables offline imaging in complex geometries (e.g., radioactive pipelines) or hazardous environments (e.g., nuclear accident sites) through carrier trap-mediated energy storage. However, the inherent carrier trap recombination dynamics introduce prolonged decay time characteristics (ranging from minutes to hours), which inevitably compromise imaging quality through progressive signal blurring and spatial resolution degradation. This challenge requires developing advanced light management methods coupled with image processing algorithms that strategically compromise between delayed detection capacity and imaging resolution enhancement. Moreover, current methods for reading out the light signals to achieve delayed detection primarily involve pressure, laser, and heating techniques. To bridge this implementation gap, future research must prioritize the integration of MHPs scintillators with compact detector for delayed signal extraction and temporal–spatial correlation processing.
6. Machine learning and artificial intelligence are revolutionizing the field of X-ray detection by reducing image SNR and accelerating image recognition speed, significantly enhancing production efficiency and detection accuracy. On the one hand, deep learning-based image denoising algorithms, such as convolutional neural networks (CNNs) and generative adversarial networks (GANs), can automatically identify and remove noise from images while preserving important structural information, thereby improving the SNR of images. Adaptive filtering techniques, along with data augmentation and regularization methods, further enhance the robustness and generalization capabilities of image processing. On the other hand, deep learning models can rapidly extract key features from images, and optimized lightweight network architectures combined with hardware acceleration technologies enable real-time processing of X-ray images, driving the automation of the entire workflow from image acquisition to diagnostic result generation. Future work should focus on integrating these technologies to further enhance detection precision and efficiency while addressing computational complexity and model interpretability challenges.

## References

[1] X. Ou, X. Chen, X. Xu, L. Xie, X. Chen et al., Recent development in X-ray imaging technology: future and challenges. Research **2021**, 9892152 (2021). 10.34133/2021/9892152 35028585 10.34133/2021/9892152 PMC8724686

[2] Y. Wu, J. Feng, Z. Yang, Y. Liu, S. Liu, Halide perovskite: a promising candidate for next-generation X-ray detectors. Adv. Sci. **10**(1), 2205536 (2023). 10.1002/advs.202205536 10.1002/advs.202205536 PMC9811474 36453564

[3] J. Liu, B. Shabbir, C. Wang, T. Wan, Q. Ou et al., Flexible, printable soft-X-ray detectors based on all-inorganic perovskite quantum dots. Adv. Mater. **31**(30), e1901644 (2019). 10.1002/adma.201901644 31169936 10.1002/adma.201901644

[4] B. Yang, L. Yin, G. Niu, J.-H. Yuan, K.-H. Xue et al., Lead-free halide Rb_2_CuBr_3_ as sensitive X-ray scintillator. Adv. Mater. **31**(44), 1904711 (2019). 10.1002/adma.201904711 10.1002/adma.201904711 31531905

[5] Q. Zhou, W. Li, J. Xiao, A. Li, X. Han, Low-dimensional metal halide for high performance scintillators. Adv. Funct. Mater. **34**(38), 2402902 (2024). 10.1002/adfm.202402902

[6] Y.C. Kim, K.H. Kim, D.Y. Son, D.N. Jeong, J.Y. Seo et al., Printable organometallic perovskite enables large-area, low-dose X-ray imaging. Nature **550**(7674), 87–91 (2017). 10.1038/nature24032 28980632 10.1038/nature24032

[7] K. Dong, T. Jiang, G. Chen, H. Cui, S. Wang et al., Light management in 2D perovskite toward high-performance optoelectronic applications. Nano-Micro Lett. **17**(1), 131 (2025). 10.1007/s40820-024-01643-7 10.1007/s40820-024-01643-7 PMC11799501 39909995

[8] S. Tsuda, K. Saito, Spectrum–dose conversion operator of NaI(Tl) and CsI(Tl) scintillation detectors for air dose rate measurement in contaminated environments. J. Environ. Radioact. **166**, 419–426 (2017). 10.1016/j.jenvrad.2016.02.008 26952947 10.1016/j.jenvrad.2016.02.008

[9] V.V. Nagarkar, T.K. Gupta, S. Miller, Y. Klugarman, M.R. Squillante et al., Structured CsI(Tl) scintillators for X-ray imaging applications. In 1997 IEEE nuclear science symposium conference record. November 9-15, 1997, Albuquerque, NM, USA. IEEE, (1997), 226–230

[10] C. Greskovich, S. Duclos, Ceramic scintillators. Annu. Rev. Mater. Sci. **27**, 69–88 (1997). 10.1146/annurev.matsci.27.1.69

[11] C. Michail, V. Koukou, N. Martini, G. Saatsakis, N. Kalyvas et al., Luminescence efficiency of cadmium tungstate (CdWO_4_) single crystal for medical imaging applications. Crystals **10**(6), 429 (2020). 10.3390/cryst10060429

[12] C. Michail, I. Valais, I. Seferis, N. Kalyvas, S. David et al., Measurement of the luminescence properties of Gd_2_O_2_S: Pr, Ce, F powder scintillators under X-ray radiation. Radiat. Meas. **70**, 59–64 (2014). 10.1016/j.radmeas.2014.09.008

[13] S. Blahuta, B. Viana, A. Bessière, E. Mattmann, B. LaCourse, Luminescence quenching processes in Gd_2_O_2_S: Pr^3+^, Ce^3+^ scintillating ceramics. Opt. Mater. **33**(10), 1514–1518 (2011). 10.1016/j.optmat.2011.02.040

[14] G. Borghi, B.J. Peet, V. Tabacchini, D.R. Schaart, A 32 mm × 32 mm × 22 mm monolithic LYSO: Ce detector with dual-sided digital photon counter readout for ultrahigh-performance TOF-PET and TOF-PET/MRI. Phys. Med. Biol. **61**(13), 4929 (2016). 10.1088/0031-9155/61/13/4929 27286232 10.1088/0031-9155/61/13/4929

[15] D.N. ter Weele, D.R. Schaart, P. Dorenbos, Comparative study of Co-doped and non Co-doped LSO: Ce and LYSO: Ce scintillators for TOF-PET. IEEE Trans. Nucl. Sci. **62**(3), 727–731 (2015). 10.1109/TNS.2015.2431295

[16] H. Wu, Y. Ge, G. Niu, J. Tang, Metal halide perovskites for X-ray detection and imaging. Matter **4**(1), 144–163 (2021). 10.1016/j.matt.2020.11.015

[17] S. Yakunin, M. Sytnyk, D. Kriegner, S. Shrestha, M. Richter et al., Detection of X-ray photons by solution-processed lead halide perovskites. Nat. Photonics **9**(7), 444–449 (2015). 10.1038/nphoton.2015.82 28553368 10.1038/nphoton.2015.82 PMC5444515

[18] K. Shibuya, M. Koshimizu, Y. Takeoka, K. Asai, Scintillation properties of C_6_H_13_NH_3_)_2_PbI_4_: exciton luminescence of an organic/inorganic multiple quantum well structure compound induced by 20 MeV protons. Nucl. Instrum. Meth. Phys. Res. Sect. B Beam Interact. Mater. At. **194**(2), 207–212 (2002). 10.1016/S0168-583X(02)00671-7

[19] M.D. Birowosuto, D. Cortecchia, W. Drozdowski, K. Brylew, W. Lachmanski et al., X-ray scintillation in lead halide perovskite crystals. Sci. Rep. **6**, 37254 (2016). 10.1038/srep37254 27849019 10.1038/srep37254 PMC5111063

[20] F. Zhou, Z. Li, W. Lan, Q. Wang, L. Ding et al., Halide perovskite, a potential scintillator for X-ray detection. Small Meth. **4**(10), 2000506 (2020). 10.1002/smtd.202000506

[21] J.H. Heo, D.H. Shin, J.K. Park, D.H. Kim, S.J. Lee et al., High-performance next-generation perovskite nanocrystal scintillator for nondestructive X-ray imaging. Adv. Mater. (2018). 10.1002/adma.201801743 10.1002/adma.201801743 30141200

[22] H. Zhang, Z. Yang, M. Zhou, L. Zhao, T. Jiang et al., Reproducible X-ray imaging with a perovskite nanocrystal scintillator embedded in a transparent amorphous network structure. Adv. Mater. **33**(40), 2102529 (2021). 10.1002/adma.202102529 10.1002/adma.202102529 34418177

[23] H. Li, Y. Zhang, M. Zhou, H. Ding, L. Zhao et al., A solar-blind perovskite scintillator realizing portable X-ray imaging. ACS Energy Lett. **7**(9), 2876–2883 (2022). 10.1021/acsenergylett.2c01440

[24] G.E. Eperon, K.H. Stone, L.E. Mundt, T.H. Schloemer, S.N. Habisreutinger et al., The role of dimethylammonium in bandgap modulation for stable halide perovskites. ACS Energy Lett. **5**(6), 1856–1864 (2020). 10.1021/acsenergylett.0c00872

[25] E. Oksenberg, A. Merdasa, L. Houben, I. Kaplan-Ashiri, A. Rothman et al., Large lattice distortions and size-dependent bandgap modulation in epitaxial halide perovskite nanowires. Nat. Commun. **11**(1), 489 (2020). 10.1038/s41467-020-14365-2 31980620 10.1038/s41467-020-14365-2 PMC6981217

[26] K. Kim, H. Kim, J. Park, Bandgap modulation of Cs_2_AgInX_6_ (X = Cl and Br) double perovskite nano- and microcrystals *via* Cu^2+^ doping. ACS Omega **6**(41), 26952–26958 (2021). 10.1021/acsomega.1c03290 34853820 10.1021/acsomega.1c03290 PMC8628852

[27] F. Ruf, P. Rietz, M.F. Aygüler, I. Kelz, P. Docampo et al., The bandgap as a moving target: reversible bandgap instabilities in multiple-cation mixed-halide perovskite solar cells. ACS Energy Lett. **3**(12), 2995–3001 (2018). 10.1021/acsenergylett.8b01857

[28] Z. Wang, J. Qiu, X. Wang, Z. Zhang, Y. Chen et al., Two-dimensional light-emitting materials: preparation, properties and applications. Chem. Soc. Rev. **47**(16), 6128–6174 (2018). 10.1039/c8cs00332g 30059108 10.1039/c8cs00332g

[29] A. Xie, F. Maddalena, M.E. Witkowski, M. Makowski, B. Mahler et al., Library of two-dimensional hybrid lead halide perovskite scintillator crystals. Chem. Mater. **32**(19), 8530–8539 (2020). 10.1021/acs.chemmater.0c02789

[30] A. Wibowo, M.A.K. Sheikh, L.J. Diguna, M.B. Ananda, M.A. Marsudi et al., Development and challenges in perovskite scintillators for high-resolution imaging and timing applications. Commun. Mater. **4**, 21 (2023). 10.1038/s43246-023-00348-5

[31] M. Moszynski, M. Kapusta, M. Mayhugh, D. Wolski, S.O. Flyckt, Absolute light output of scintillators. IEEE Trans. Nucl. Sci. **44**(3), 1052–1061 (1997). 10.1109/23.603803

[32] S. Liu, F. Jiang, E. Song, P. Li, W. Liu et al., Controllable preparation and enhanced luminescence of Tb^3+^-activated transparent gadolinium-rich silicate glass ceramic scintillator. Ceram. Int. (2025). 10.1016/j.ceramint.2025.01.413

[33] T. He, Y. Zhou, X. Wang, J. Yin, L. Gutiérrez-Arzaluz et al., High-performance copper-doped perovskite-related silver halide X-ray imaging scintillator. ACS Energy Lett. **7**(8), 2753–2760 (2022). 10.1021/acsenergylett.2c01484

[34] E. Auffray, G. Dosovitskiy, A. Fedorov, I. Guz, M. Korjik et al., Irradiation effects on Gd_3_Al_2_Ga_3_O_12_ scintillators prospective for application in harsh irradiation environments. Radiat. Phys. Chem. **164**, 108365 (2019). 10.1016/j.radphyschem.2019.108365

[35] W. Yan, B. Duan, Z. Zhu, Y. Song, G. Song et al., Organic–inorganic hybrid perovskite scintillator for high-resolution X-ray imaging. Nucl. Instrum. Meth. Phys. Res. Sect. B Beam Interact. Mater. At. **546**, 165159 (2024). 10.1016/j.nimb.2023.165159

[36] V. Dormenev, K.T. Brinkmann, D. Kazlou, M. Moritz, R.W. Novotny et al., Scintillation properties of garnets and oxyorthosilicates with different dopants. IEEE Trans. Nucl. Sci. **70**(7), 1392–1397 (2023). 10.1109/TNS.2023.3275642

[37] F. Maddalena, A. Xie, X.Y. Chin, R. Begum, M.E. Witkowski et al., Deterministic light yield, fast scintillation, and microcolumn structures in lead halide perovskite nanocrystals. J. Phys. Chem. C **125**(25), 14082–14088 (2021). 10.1021/acs.jpcc.1c03392

[38] W. Chen, M. Zhou, Y. Liu, X. Yu, C. Pi et al., All-inorganic perovskite polymer–ceramics for flexible and refreshable X-ray imaging. Adv. Funct. Mater. **32**(2), 2107424 (2022). 10.1002/adfm.202107424

[39] Y. Li, L. Chen, B. Liu, P. Jin, R. Gao et al., Scintillation performance of two-dimensional perovskite (BA)_2_PbBr_4_ microcrystals. J. Mater. Chem. C **9**(47), 17124–17128 (2021). 10.1039/D1TC04072C

[40] W. Shao, X. Wang, Z. Zhang, J. Huang, Z. Han et al., Highly efficient and flexible scintillation screen based on manganese (II) activated 2D perovskite for planar and nonplanar high-resolution X-ray imaging. Adv. Opt. Mater. **10**(6), 2102282 (2022). 10.1002/adom.202102282

[41] N. Li, Z. Xu, Y. Xiao, Y. Liu, Z. Yang et al., Flexible, high scintillation yield Cu_3_Cu_2_I_5_ film made of ball-milled powder for high spatial resolution X-ray imaging. Adv. Opt. Mater. **10**(5), 2102232 (2022). 10.1002/adom.202102232

[42] J.J. van Blaaderen, L.A. van den Brekel, K.W. Krämer, P. Dorenbos, Scintillation and optical characterization of CsCu_2_I_3_ single crystals from 10 to 400 K. Chem. Mater. **35**(22), 9623–9631 (2023). 10.1021/acs.chemmater.3c01810 38047185 10.1021/acs.chemmater.3c01810 PMC10687859

[43] P. Ran, X. Chen, Z. Chen, Y. Su, J. Hui et al., Metal halide CsCu2I3 flexible scintillator with high photodiode spectral compatibility for X-ray cone beam computed tomography (CBCT) imaging. Laser Photonics Rev. **18**(1), 2300743 (2024). 10.1002/lpor.202300743

[44] R. Zhang, H. Xie, W. Liu, K. Zhan, H. Liu et al., High-efficiency narrow-band green-emitting manganese(II) halide for multifunctional applications. ACS Appl. Mater. Interfaces **15**(40), 47238–47249 (2023). 10.1021/acsami.3c09518 37768211 10.1021/acsami.3c09518

[45] Z. Gong, J. Zhang, X. Deng, M.-P. Ren, W.-Q. Wang et al., Near-unity broadband emissive hybrid manganese bromides as highly-efficient radiation scintillators. Aggregate **5**(5), e574 (2024). 10.1002/agt2.574

[46] B. Su, J. Jin, K. Han, Z. Xia, Ceramic wafer scintillation screen by utilizing near-unity blue-emitting lead-free metal halide (C_8_H_2_0N)_2_Cu_2_Br_4_. Adv. Funct. Mater. **33**(5), 2210735 (2023). 10.1002/adfm.202210735

[47] M. Zhou, H. Jiang, T. Hou, S. Hou, J. Li et al., Inch-size and thickness-adjustable hybrid manganese halide single-crystalline films for high resolution X-ray imaging. Chem. Eng. J. **490**, 151823 (2024). 10.1016/j.cej.2024.151823

[48] F. Zhang, Y. Zhou, Z. Chen, M. Wang, Z. Ma et al., Thermally activated delayed fluorescence zirconium-based perovskites for large-area and ultraflexible X-ray scintillator screens. Adv. Mater. **34**(43), 2204801 (2022). 10.1002/adma.202204801 10.1002/adma.202204801 36047911

[49] H. Wu, Q. Wang, A. Zhang, G. Niu, M. Nikl et al., One-dimensional scintillator film with benign grain boundaries for high-resolution and fast X-ray imaging. Sci. Adv. **9**(30), eadh1789 (2023). 10.1126/sciadv.adh1789 37506201 10.1126/sciadv.adh1789 PMC10381942

[50] Y. Zhou, J. Chen, O.M. Bakr, O.F. Mohammed, Metal halide perovskites for X-ray imaging scintillators and detectors. ACS Energy Lett. **6**(2), 739–768 (2021). 10.1021/acsenergylett.0c02430

[51] A. Jana, S. Cho, S.A. Patil, A. Meena, Y. Jo et al., Perovskite: Scintillators, direct detectors, and X-ray imagers. Mater. Today **55**, 110–136 (2022). 10.1016/j.mattod.2022.04.009

[52] C. Thalhammer, Improving the light yield and timing resolution of scintillator-based detectors for positron emission tomography. IEEE Trans. Neural Networks Learn. Syst. (2015). 10.1109/tnnls.2023.3323131

[53] M. Nikl, A. Yoshikawa, Recent R&D trends in inorganic single-crystal scintillator materials for radiation detection. Adv. Opt. Mater. **3**(4), 463–481 (2015). 10.1002/adom.201400571

[54] Y. Wang, M. Li, Z. Chai, Y. Wang, S. Wang, Perovskite scintillators for improved X-ray detection and imaging. Angew. Chem. **135**(38), e202304638 (2023). 10.1002/ange.202304638 10.1002/anie.202304638 37258939

[55] A. Anand, M.L. Zaffalon, A. Erroi, F. Cova, F. Carulli et al., Advances in perovskite nanocrystals and nanocomposites for scintillation applications. ACS Energy Lett. **9**(3), 1261–1287 (2024). 10.1021/acsenergylett.3c02763

[56] J. Ye, D. Gaur, C. Mi, Z. Chen, I.L. Fernández et al., Strongly-confined colloidal lead-halide perovskite quantum dots: from synthesis to applications. Chem. Soc. Rev. **53**(16), 8095–8122 (2024). 10.1039/D4CS00077C 38894687 10.1039/d4cs00077c

[57] W. Chen, Y. Liu, Z. Yuan, Z. Xu, Z. Zhang et al., X-ray radioluminescence effect of all-inorganic halide perovskite CsPbBr_3_ quantum dots. J. Radioanal. Nucl. Chem. **314**(3), 2327–2337 (2017). 10.1007/s10967-017-5562-x

[58] Q. Chen, J. Wu, X. Ou, B. Huang, J. Almutlaq et al., All-inorganic perovskite nanocrystal scintillators. Nature **561**(7721), 88–93 (2018). 10.1038/s41586-018-0451-1 30150772 10.1038/s41586-018-0451-1

[59] Y. Zhang, R. Sun, X. Ou, K. Fu, Q. Chen et al., Metal halide perovskite nanosheet for X-ray high-resolution scintillation imaging screens. ACS Nano **13**(2), 2520–2525 (2019). 10.1021/acsnano.8b09484 30721023 10.1021/acsnano.8b09484

[60] W. Ma, T. Jiang, Z. Yang, H. Zhang, Y. Su et al., Highly resolved and robust dynamic X-ray imaging using perovskite glass-ceramic scintillator with reduced light scattering. Adv. Sci. **8**(15), e2003728 (2021). 10.1002/advs.202003728 10.1002/advs.202003728 PMC8336613 34075729

[61] H. Zhang, Z. Yang, M. Zhou, L. Zhao, T. Jiang, H. Yang, Y. Xue, J. Qiu, Reproducible X‐ray imaging with a perovskite nanocrystal scintillator embedded in a transparent amorphous network structure. Adv. Mater. (2021). 10.1002/adma.202102529 10.1002/adma.202102529 34418177

[62] Y. Li, Y. Lei, H. Wang, Z. Jin, Two-dimensional metal halides for X-ray detection applications. Nano-Micro Lett. **15**(1), 128 (2023). 10.1007/s40820-023-01118-1 10.1007/s40820-023-01118-1 PMC10199999 37209282

[63] B. Jia, D. Chu, N. Li, Y. Zhang, Z. Yang et al., Airflow-controlled crystallization for a multi-inch 2D halide perovskite single-crystal scintillator for fast high-resolution X-ray imaging. ACS Energy Lett. **8**(1), 590–599 (2023). 10.1021/acsenergylett.2c02196

[64] M. Xia, Z. Xie, H. Wang, T. Jin, L. Liu et al., Sub-nanosecond 2D perovskite scintillators by dielectric engineering. Adv. Mater. **35**(18), e2211769 (2023). 10.1002/adma.202211769 36762587 10.1002/adma.202211769

[65] M.A. Kuddus Sheikh, F. Maddalena, D. Kowal, M. Makowski, S. Mahato et al., Effect of dual-organic cations on the structure and properties of 2D hybrid perovskites as scintillators. ACS Appl. Mater. Interfaces **16**(19), 25529–25539 (2024). 10.1021/acsami.4c01741 38698765 10.1021/acsami.4c01741 PMC11103655

[66] W. Shao, Q. Li, T. He, Y. Zhang, M. Niu et al., Synergy of organic and inorganic sites in 2D perovskite for fast neutron and X-ray imaging. Adv. Funct. Mater. **33**(40), 2301767 (2023). 10.1002/adfm.202301767

[67] W. Zhu, W. Ma, Y. Su, Z. Chen, X. Chen et al., Low-dose real-time X-ray imaging with nontoxic double perovskite scintillators. Light Sci. Appl. **9**, 112 (2020). 10.1038/s41377-020-00353-0 32637079 10.1038/s41377-020-00353-0 PMC7327019

[68] Y.-H. Liu, N.-N. Wang, M.-P. Ren, X. Yan, Y.-F. Wu et al., Zero-dimensional hybrid cuprous halide of [BAPMA] Cu_2_Br_5_ as a highly efficient light emitter and an X-ray scintillator. ACS Appl. Mater. Interfaces **15**(16), 20219–20227 (2023). 10.1021/acsami.3c00206 37062879 10.1021/acsami.3c00206

[69] F. Zhang, Y. Zhou, Z. Chen, X. Niu, H. Wang et al., Large-area X-ray scintillator screen based on cesium hafnium chloride microcrystals films with high sensitivity and stability. Laser Photonics Rev. **17**(5), 2200848 (2023). 10.1002/lpor.202200848

[70] L. Zi, J. Song, N. Wang, T. Wang, W. Li et al., X-ray quantum cutting scintillator based on CsPbCl_*x*_Br_3__*–*_x: Y^b3^+ single crystals. Laser Photonics Rev. **17**(5), 2200852 (2023). 10.1002/lpor.202200852

[71] D.-Y. Li, Y.-B. Shang, Q. Liu, H.-W. Zhang, X.-Y. Zhang et al., 0D hybrid indium halide as a highly efficient X-ray scintillation and ultra-sensitive fluorescent probe. Mater. Horiz. **10**(11), 5004–5015 (2023). 10.1039/D3MH00536D 37642515 10.1039/d3mh00536d

[72] S. Wang, W. Huang, X. Liu, S. Wang, H. Ye et al., Ruddlesden–popper perovskite nanocrystals stabilized in mesoporous silica with efficient carrier dynamics for flexible X-ray scintillator. Adv. Funct. Mater. **33**(3), 2210765 (2023). 10.1002/adfm.202210765

[73] P. Ran, L. Yang, T. Jiang, X. Xu, J. Hui et al., Multispectral large-panel X-ray imaging enabled by stacked metal halide scintillators. Adv. Mater. **34**(42), e2205458 (2022). 10.1002/adma.202205458 35963008 10.1002/adma.202205458

[74] X. Wang, X. Zhang, S. Yan, H. Liu, Y. Zhang, Nearly-unity quantum yield and 12-hour afterglow from a transparent perovskite of Cs_2_NaScCl_6_: Tb. Angew. Chem. Int. Ed. **134**(40), e202210853 (2022). 10.1002/ange.202210853 10.1002/anie.202210853 35951470

[75] Z. Zhang, Q. Han, J.W. Lau, B. Xing, Lanthanide-doped upconversion nanoparticles meet the needs for cutting-edge bioapplications: recent progress and perspectives. ACS Mater. Lett. **2**(11), 1516–1531 (2020). 10.1021/acsmaterialslett.0c00377

[76] L. Han, B. Sun, C. Guo, G. Peng, H. Chen et al., Photophysics in zero-dimensional potassium-doped cesium copper chloride Cs_3_Cu_2_Cl_5_ nanosheets and its application for high-performance flexible X-Ra_*y*_ detection. Adv. Opt. Mater. **10**(6), 2102453 (2022). 10.1002/adom.202102453

[77] W. Chen, T. Wang, T. Wang, J. Yu, S. Yao et al., Customizable scintillator of Cs_3_Cu_2_I_5_: 2% In^+^ @Paper for large-area X-ray imaging. Adv. Sci. **10**(34), e2304957 (2023). 10.1002/advs.202304957 10.1002/advs.202304957 PMC10700220 37870217

[78] V. Naresh, P.-R. Cha, N. Lee, Cs_2_NaGdCl_6_: Tb^3+^─A highly luminescent rare-earth double perovskite scintillator for low-dose X-ray detection and imaging. ACS Appl. Mater. Interfaces **16**(15), 19068–19080 (2024). 10.1021/acsami.3c17301 38587167 10.1021/acsami.3c17301

[79] W. Li, L. Yuan, H. Wu, Y. Jin, Tb^3+^-doped a zero-dimensional all-inorganic metal halide perovskite scintillator Cs_2_ScCl_5_·H_2_O for X-ray imaging. Opt. Mater. **157**, 116130 (2024). 10.1016/j.optmat.2024.116130

[80] J. Shi, Z. Wang, L. Xu, J. Wang, Z. Da et al., *In situ* growth of lead-free double perovskite micron sheets in polymethyl methacrylate for X-ray imaging. Adv. Opt. Mater. **12**(22), 2400691 (2024). 10.1002/adom.202400691

[81] Y.-Y. Wang, W.-T. Song, X.-R. Yao, X.-Y. Chen, M.-T. Cheng et al., A new polar lead-free hybrid halide X-ray scintillator. Adv. Opt. Mater. **12**(22), 2400190 (2024). 10.1002/adom.202400190

[82] T. Feng, Z.-A. Zhou, Y.-N. An, L. Chen, Y. Fu et al., Large-area transparent antimony-based perovskite glass for high-resolution X-ray imaging. ACS Nano **18**(26), 16715–16725 (2024). 10.1021/acsnano.4c01761 38876985 10.1021/acsnano.4c01761

[83] J. Li, Q. Hu, J. Xiao, Z.-G. Yan, High-stability double perovskite scintillator for flexible X-ray imaging. J. Colloid Interface Sci. **671**, 725–731 (2024). 10.1016/j.jcis.2024.05.203 38823113 10.1016/j.jcis.2024.05.203

[84] Y. Zhang, K. Li, L. Niu, J. Ren, X. Liu et al., Eu^2+^ doped cesium alkaline earth chloride nanocrystals in glass for X-ray imaging. Ceram. Int. **50**(17), 31673–31679 (2024). 10.1016/j.ceramint.2024.05.475

[85] W. Bu, Y. Yan, P. Liu, T. Wang, S. Wang et al., *In-situ* growth of Cs_5_Cu_3_Cl_6_I_2_ nanocrystals within AAO arrays for X-ray imaging. Chem. Eng. J. **492**, 151908 (2024). 10.1016/j.cej.2024.151908

[86] Y. Zhang, M. Shen, B. Cheng, W. Ma, X. Huang et al., Ultrafast scintillation at room temperature achieved in CsPbCl_3_-based single crystals through Br over-doping. J. Mater. Chem. C **12**(20), 7169–7175 (2024). 10.1039/D4TC00880D

[87] J.-A. Lai, P. Wang, B. Zheng, T. Xuan, D. Wu et al., Enhanced performance in cesium tellurium chlorine by hafnium alloying for X-ray computed tomography imaging. Adv. Opt. Mater. **12**(17), 2303297 (2024). 10.1002/adom.202303297

[88] Y. Wang, C. Wang, L. Men, Q. Hu, J. Xiao, Colloidal synthesis of hollow double perovskite nanocrystals and their applications in X-ray imaging. Inorg. Chem. **63**(12), 5734–5742 (2024). 10.1021/acs.inorgchem.4c00280 38478658 10.1021/acs.inorgchem.4c00280

[89] J. Qiu, H. Zhao, Z. Mu, J. Chen, H. Gu et al., Turning nonemissive CsPb_2_Br_5_ crystals into high-performance scintillators through alkali metal doping. Nano Lett. **24**(8), 2503–2510 (2024). 10.1021/acs.nanolett.3c04455 38258747 10.1021/acs.nanolett.3c04455

[90] W. Xiang, D. Shen, X. Zhang, X. Li, Y. Liu et al., Transparent and planar Cs_3_Cu_2_Cl_5_ crystals for micrometer-resolution X-ray imaging screen. ACS Appl. Mater. Interfaces **16**(4), 4918–4924 (2024). 10.1021/acsami.3c15764 38237115 10.1021/acsami.3c15764

[91] M. Wang, X. Qing, T. Du, C. Wu, X. Han, Te4^+^-doped Cs_2_SnCl_6_ scintillator for flexible and efficient X-ray imaging screens. J. Mater. Chem. C **12**(6), 2241–2246 (2024). 10.1039/D3TC03818A

[92] N. Liu, J. Luo, Q. Guo, P. Du, L. Gao et al., Reduced self-absorption of quasi-2D perovskites and their application in color conversion layers. Adv. Opt. Mater. **11**(1), 2202118 (2023). 10.1002/adom.202202118

[93] T. Haposan, A. Arramel, P.Y.D. Maulida, S. Hartati, A.A. Afkauni et al., All-inorganic copper-halide perovskites for large-Stokes shift and ten-nanosecond-emission scintillators. J. Mater. Chem. C **12**(7), 2398–2409 (2024). 10.1039/D3TC03977C

[94] S. Cheng, M. Nikl, A. Beitlerova, R. Kucerkova, X. Du et al., Ultrabright and highly efficient all-inorganic zero-dimensional perovskite scintillators. Adv. Opt. Mater. **9**(13), 2100460 (2021). 10.1002/adom.202100460

[95] W.J. Mir, Y. Mahor, A. Lohar, M. Jagadeeswararao, S. Das et al., Postsynthesis doping of Mn and Yb into CsPbX_3_ (X = Cl, Br, or I) perovskite nanocrystals for downconversion emission. Chem. Mater. **30**(22), 8170–8178 (2018). 10.1021/acs.chemmater.8b03066

[96] V. Pinchetti, A. Anand, Q.A. Akkerman, D. Sciacca, M. Lorenzon et al., Trap-mediated two-step sensitization of manganese dopants in perovskite nanocrystals. ACS Energy Lett. **4**(1), 85–93 (2019). 10.1021/acsenergylett.8b02052

[97] W. Liu, Q. Lin, H. Li, K. Wu, I. Robel et al., Mn^2+^-doped lead halide perovskite nanocrystals with dual-color emission controlled by halide content. J. Am. Chem. Soc. **138**(45), 14954–14961 (2016). 10.1021/jacs.6b08085 27756131 10.1021/jacs.6b08085

[98] S. Das Adhikari, A.K. Guria, N. Pradhan, Insights of doping and the photoluminescence properties of Mn-doped perovskite nanocrystals. J. Phys. Chem. Lett. **10**(9), 2250–2257 (2019). 10.1021/acs.jpclett.9b00182 30990324 10.1021/acs.jpclett.9b00182

[99] S. Paul, E. Bladt, A.F. Richter, M. Döblinger, Y. Tong et al., Manganese-doping-induced quantum confinement within host perovskite nanocrystals through ruddlesden–popper defects. Angew. Chem. Int. Ed. **59**(17), 6794–6799 (2020). 10.1002/anie.201914473 10.1002/anie.201914473 PMC7186832 32003102

[100] G. Huang, C. Wang, S. Xu, S. Zong, J. Lu et al., Postsynthetic doping of MnCl_2_ molecules into preformed CsPbBr_3_ perovskite nanocrystals* vi*a a halide exchange-driven cation exchange. Adv. Mater. **29**(29), 1700095 (2017). 10.1002/adma.201700095 10.1002/adma.201700095 28585275

[101] H. Xu, W. Liang, Z. Zhang, C. Cao, W. Yang et al., 2D perovskite Mn^2+^-doped Cs_2_CdBr_2_Cl_2_ scintillator for low-dose high-resolution X-ray imaging. Adv. Mater. **35**(26), e2300136 (2023). 10.1002/adma.202300136 36971078 10.1002/adma.202300136

[102] K. Li, W. Zhang, L. Niu, Y. Ye, J. Ren et al., Lead-free cesium manganese halide nanocrystals embedded glasses for X-ray imaging. Adv. Sci. **10**(4), 2204843 (2023). 10.1002/advs.202204843 10.1002/advs.202204843 PMC9896042 36461760

[103] D.J. Kiebala, R. Style, D. Vanhecke, C. Calvino, C. Weder et al., Sub-micrometer mechanochromic inclusions enable strain sensing in polymers. Adv. Funct. Mater. **33**(50), 2304938 (2023). 10.1002/adfm.202304938

[104] B. Wang, X. Ouyang, X. He, Z. Deng, Y. Zhou et al., High-resolution X-ray imaging of Mn enhanced lead-free halide scintillators in pixelated array structures. Adv. Opt. Mater. **11**(17), 2300388 (2023). 10.1002/adom.202300388

[105] N. Varnakavi, R. Rajavaram, K. Gupta, P.-R. Cha, N. Lee, Scintillation performance of Mn(II)-doped Cs_2_NaBiCl_6_ double perovskite nanocrystals for X-Ra_*y*_ imaging applications. Adv. Opt. Mater. **12**(9), 2301868 (2024). 10.1002/adom.202301868

[106] C. Wang, Z.-G. Yan, Y. Wang, J. Zhu, C. Peng et al., All-inorganic ruddlesden–popper perovskite Cs_2_CdCl_4_: Mn for low-dose and flexible X-ray imaging. ACS Mater. Lett. **6**(4), 1429–1438 (2024). 10.1021/acsmaterialslett.3c01665

[107] P. Pei, R. Wei, B. Wang, J. Su, Z. Zhang et al., An advanced tunable multimodal luminescent La_4_GeO_8_: Eu^2+^, Er^3+^ phosphor for multicolor anticounterfeiting. Adv. Funct. Mater. **31**(31), 2102479 (2021). 10.1002/adfm.202102479

[108] M.P. Davydova, L. Meng, M.I. Rakhmanova, Z. Jia, A.S. Berezin et al., Strong magnetically-responsive circularly polarized phosphorescence and X-ray scintillation in ultrarobust Mn(II)–organic helical chains. Adv. Mater. **35**(35), 2303611 (2023). 10.1002/adma.202303611 10.1002/adma.202303611 37358067

[109] H. Tang, Z. Jia, Y. Xu, Y. Liu, Q. Lin, Enhanced photoluminescence quantum yield of metal halide perovskite microcrystals for multiple optoelectronic applications. Small **20**(4), 2304336 (2024). 10.1002/smll.202304336 10.1002/smll.202304336 37712103

[110] Z. Wang, Y. Wei, C. Liu, Y. Liu, M. Hong, Mn^2+^-activated Cs_3_Cu_2_I_5_ nano-scintillators for ultra-high resolution flexible X-Ra_*y*_ imaging. Laser Photonics Rev. **17**(6), 2200851 (2023). 10.1002/lpor.202200851

[111] L. Li, Z. Fan, J. Zhang, D. Fan, X. Liu et al., Yellow emissive CsCu_2_I_3_ nanocrystals induced by Mn^2+^ for high-resolution X-ray imaging. Inorg. Chem. **62**(49), 19848–19855 (2023). 10.1021/acs.inorgchem.3c03724 38032318 10.1021/acs.inorgchem.3c03724

[112] J. Barrio, A. Pedersen, S.C. Sarma, A. Bagger, M. Gong et al., FeNC oxygen reduction electrocatalyst with high utilization penta-coordinated sites. Adv. Mater. **35**(14), e2211022 (2023). 10.1002/adma.202211022 36739474 10.1002/adma.202211022

[113] J.-X. Zheng, Z.-A. Zhou, T. Feng, H. Li, C.-H. Sun et al., Hydrophobic long-chain two-dimensional perovskite scintillators for underwater X-ray imaging. Rare Met. **43**(1), 175–185 (2024). 10.1007/s12598-023-02421-x

[114] N. Kawano, M. Akatsuka, H. Kimura, D. Nakauchi, T. Kato et al., Scintillation properties of Mn-doped methylammonium lead chloride crystals. J. Mater. Sci. Mater. Electron. **32**(10), 12903–12910 (2021). 10.1007/s10854-020-04480-7

[115] X. Li, H. Guo, Y. Li, C. Lin, L. Xie, Enhancing persistent radioluminescence in perovskite scintillators through trap defect modulation. Mater. Chem. Front. **8**(13), 2539–2548 (2024). 10.1039/D4QM00039K

[116] B. Su, K. Han, Z. Xia, Mn^2+^-doped Cs_2_ZnBr_4_ scintillator for X-ray imaging. J. Mater. Chem. C **11**(24), 8052–8061 (2023). 10.1039/D2TC04249E

[117] K.E. Yorov, W.J. Mir, X. Song, L. Gutiérrez-Arzaluz, R. Naphade et al., Mn^4+^-doped fluoride nanocrystals enable high-resolution red-emitting X-ray imaging screens. ACS Mater. Lett. **4**(11), 2273–2281 (2022). 10.1021/acsmaterialslett.2c00746

[118] L.-J. Xu, X. Lin, Q. He, M. Worku, B. Ma, Highly efficient eco-friendly X-ray scintillators based on an organic manganese halide. Nat. Commun. **11**(1), 4329 (2020). 10.1038/s41467-020-18119-y 32859920 10.1038/s41467-020-18119-y PMC7455565

[119] J. Jin, K. Han, Y. Hu, Z. Xia, Zn^2+^ doping in organic manganese(II) bromide hybrid scintillators toward enhanced light yield for X-ray imaging. Adv. Opt. Mater. **11**(14), 2300330 (2023). 10.1002/adom.202300330

[120] J. Zhang, X. Wang, W.-Q. Wang, X. Deng, C.-Y. Yue et al., Near-unity green luminescent hybrid manganese halides as X-ray scintillators. Inorg. Chem. **63**(5), 2647–2654 (2024). 10.1021/acs.inorgchem.3c03924 38262040 10.1021/acs.inorgchem.3c03924

[121] X. Wang, X. Zhang, Y. Liu, Y. Zhang, Shape-on-demand synthesis of luminescent (ETP)_2_MnBr_4_ glass scintillator. Chem. Eng. J. **483**, 149239 (2024). 10.1016/j.cej.2024.149239

[122] Y. Xu, Z. Li, G. Peng, F. Qiu, Z. Li et al., Organic cation design of manganese halide hybrids glass toward low-temperature integrated efficient, scaling, and reproducible X-ray detector. Adv. Opt. Mater. **11**(13), 2300216 (2023). 10.1002/adom.202300216

[123] S. Wang, S. Feng, R. Li, J. Jin, J. Wu et al., Multiexciton generation from a 2D organic-inorganic hybrid perovskite with nearly 200% quantum yield of red phosphorescence. Adv. Mater. **35**(18), e2211992 (2023). 10.1002/adma.202211992 36807946 10.1002/adma.202211992

[124] F. Montanarella, K.M. McCall, K. Sakhatskyi, S. Yakunin, P. Trtik et al., Highly concentrated, zwitterionic ligand-capped Mn^2+^: CsPb(Br_*x*_Cl_1-__*x*_)_3_ nanocrystals as bright scintillators for fast neutron imaging. ACS Energy Lett. **6**(12), 4365–4373 (2021). 10.1021/acsenergylett.1c01923 34917771 10.1021/acsenergylett.1c01923 PMC8669634

[125] F. Wang, X. Liu, Multicolor tuning of lanthanide-doped nanoparticles by single wavelength excitation. Acc. Chem. Res. **47**(4), 1378–1385 (2014). 10.1021/ar5000067 24611606 10.1021/ar5000067

[126] Y. Wu, W. Wu, Combinations of superior inorganic phosphors for level-tunable information hiding and encoding. Adv. Opt. Mater. **9**(17), 2100281 (2021). 10.1002/adom.202100281

[127] B. Liu, C. Li, P. Yang, Z. Hou, J. Lin, 808-nm-light-excited lanthanide-doped nanoparticles: rational design, luminescence control and theranostic applications. Adv. Mater. **29**(18), 1605434 (2017). 10.1002/adma.201605434 10.1002/adma.201605434 28295673

[128] G. Gao, A. Turshatov, I.A. Howard, D. Busko, R. Joseph et al., Up-conversion fluorescent labels for plastic recycling: a review. Adv. Sustain. Syst. **1**(5), 1600033 (2017). 10.1002/adsu.201600033

[129] L. Lei, Y. Wang, A. Kuzmin, Y. Hua, J. Zhao et al., Next generation lanthanide doped nanoscintillators and photon converters. eLight **2**(1), 17 (2022). 10.1186/s43593-022-00024-0

[130] G. Pan, X. Bai, D. Yang, X. Chen, P. Jing et al., Doping lanthanide into perovskite nanocrystals: highly improved and expanded optical properties. Nano Lett. **17**(12), 8005–8011 (2017). 10.1021/acs.nanolett.7b04575 29182877 10.1021/acs.nanolett.7b04575

[131] M. Gandini, I. Villa, M. Beretta, C. Gotti, M. Imran et al., Efficient, fast and reabsorption-free perovskite nanocrystal-based sensitized plastic scintillators. Nat. Nanotechnol. **15**(6), 462–468 (2020). 10.1038/s41565-020-0683-8 32424340 10.1038/s41565-020-0683-8

[132] Z. Wang, S. Li, G. Ren, S. Yao, D. Zhu et al., Flexible and reabsorption-free perovskite scintillators for low-dose X-ray detection and high-resolution imaging. ACS Photonics **11**(8), 3003–3011 (2024). 10.1021/acsphotonics.4c00117

[133] Z. Yang, J. Yao, L. Xu, W. Fan, J. Song, Designer bright and fast CsPbBr_3_ perovskite nanocrystal scintillators for high-speed X-ray imaging. Nat. Commun. **15**(1), 8870 (2024). 10.1038/s41467-024-53263-9 39402070 10.1038/s41467-024-53263-9 PMC11473900

[134] H. Si, Z. Zhang, Q. Liao, G. Zhang, Y. Ou et al., A-site management for highly crystalline perovskites. Adv. Mater. **32**(4), e1904702 (2020). 10.1002/adma.201904702 31709645 10.1002/adma.201904702

[135] W.-G. Li, X.-D. Wang, Y.-H. Huang, D.-B. Kuang, Ultrasound-assisted crystallization enables large-area perovskite quasi-monocrystalline film for high-sensitive X-ray detection and imaging. Adv. Mater. **35**(31), 2210878 (2023). 10.1002/adma.202210878 10.1002/adma.202210878 37146980

[136] Z. Xiao, Q. Dong, C. Bi, Y. Shao, Y. Yuan et al., Solvent annealing of perovskite-induced crystal growth for photovoltaic-device efficiency enhancement. Adv. Mater. **26**(37), 6503–6509 (2014). 10.1002/adma.201401685 25158905 10.1002/adma.201401685

[137] J. Zhang, W. Liang, W. Yu, S. Yu, Y. Wu et al., A two-stage annealing strategy for crystallization control of CH_3_NH_3_PbI_3_ films toward highly reproducible perovskite solar cells. Small **14**(26), 1800181 (2018). 10.1002/smll.201800181 10.1002/smll.201800181 29806184

[138] S. Cui, J. Wang, H. Xie, Y. Zhao, Z. Li et al., Rubidium ions enhanced crystallinity for ruddlesden-popper perovskites. Adv. Sci. **7**(24), 2002445 (2020). 10.1002/advs.202002445 10.1002/advs.202002445 PMC7740094 33344132

[139] V. Naresh, S. Singh, H. Soh, J. Lee, N. Lee, Dual-phase CsPbBr_3_–CsPb2Br5 perovskite scintillator for sensitive X-ray detection and imaging. Mater. Today Nano **23**, 100364 (2023). 10.1016/j.mtnano.2023.100364

[140] V.B. Mykhaylyk, H. Kraus, M. Saliba, Bright and fast scintillation of organolead perovskite MAPbBr_3_ at low temperatures. Mater. Horiz. **6**(8), 1740–1747 (2019). 10.1039/C9MH00281B

[141] S. Mahato, M. Makowski, S. Bose, D. Kowal, M.A. Kuddus Sheikh et al., Improvement of light output of MAPbBr 3 single crystal for ultrafast and bright cryogenic scintillator. J. Phys. Chem. Lett. **15**(14), 3713–3720 (2024). 10.1021/acs.jpclett.4c00379 38546293 10.1021/acs.jpclett.4c00379 PMC11017313

[142] S. Kishimoto, K. Shibuya, F. Nishikido, M. Koshimizu, R. Haruki et al., Subnanosecond time-resolved X-ray measurements using an organic-inorganic perovskite scintillator. Appl. Phys. Lett. **93**(26), 261901 (2008). 10.1063/1.3059562

[143] X. Ou, X. Qin, B. Huang, J. Zan, Q. Wu et al., High-resolution X-ray luminescence extension imaging. Nature **590**(7846), 410–415 (2021). 10.1038/s41586-021-03251-6 33597760 10.1038/s41586-021-03251-6

[144] X. Zhou, K. Han, Y. Wang, J. Jin, S. Jiang et al., Energy-trapping management in X-ray storage phosphors for flexible 3D imaging. Adv. Mater. **35**(16), e2212022 (2023). 10.1002/adma.202212022 36807928 10.1002/adma.202212022

[145] L. Lu, S. Peng, L. Zhao, M. Zhang, J. Xiao et al., Visualized X-ray dosimetry for multienvironment applications. Nano Lett. **23**(18), 8753–8760 (2023). 10.1021/acs.nanolett.3c02826 37712849 10.1021/acs.nanolett.3c02826

[146] N. Zhang, R. Zhang, X. Xu, F. Wang, Z. Sun et al., X-ray-activated long afterglow double-perovskite scintillator for detection and extension imaging. Adv. Opt. Mater. **11**(16), 2300187 (2023). 10.1002/adom.202300187

[147] W. Bian, Y. Lin, T. Wang, X. Yu, J. Qiu et al., Direct identification of surface defects and their influence on the optical characteristics of upconversion nanoparticles. ACS Nano **12**(4), 3623–3628 (2018). 10.1021/acsnano.8b00741 29617571 10.1021/acsnano.8b00741

[148] C. Wang, X. Mao, Z. Wang, X. Xu, W. Guo et al., Synthesis and exciton dynamics of a one-dimensional organic copper halide perovskite. J. Phys. Chem. C **126**(10), 4959–4964 (2022). 10.1021/acs.jpcc.1c10402

[149] W. Shao, T. He, J.-X. Wang, Y. Zhou, P. Yuan et al., Transparent organic and metal halide tandem scintillators for high-resolution dual-energy X-ray imaging. ACS Energy Lett. **8**(6), 2505–2512 (2023). 10.1021/acsenergylett.3c00784

[150] X. Hu, S. Luo, J. Leng, C. Wang, Y. Chen et al., Density-discriminating chromatic X-ray imaging based on metal halide nanocrystal scintillators. Sci. Adv. **9**(37), eadh5081 (2023). 10.1126/sciadv.adh5081 37713492 10.1126/sciadv.adh5081 PMC10881070

[151] J. Hui, P. Ran, Y. Su, L. Yang, X. Xu et al., Stacked scintillators based multispectral X-ray imaging featuring quantum-cutting perovskite scintillators with 570 nm absorption-emission shift. Adv. Mater. **37**(10), e2416360 (2025). 10.1002/adma.202416360 39871685 10.1002/adma.202416360

[152] J. Pang, S. Zhao, X. Du, H. Wu, G. Niu et al., Vertical matrix perovskite X-ray detector for effective multi-energy discrimination. Light Sci. Appl. **11**(1), 105 (2022). 10.1038/s41377-022-00791-y 35449122 10.1038/s41377-022-00791-y PMC9023493

[153] B. Shabbir, J.C. Yu, T. Warnakula, R.A.W. Ayyubi, J.A. Pollock et al., Printable perovskite diodes for broad-spectrum multienergy X-ray detection. Adv. Mater. **35**(20), e2210068 (2023). 10.1002/adma.202210068 36852617 10.1002/adma.202210068

[154] W. Shao, T. He, L. Wang, J.-X. Wang, Y. Zhou et al., Capillary manganese halide needle-like array scintillator with isolated light crosstalk for micro-X-ray imaging. Adv. Mater. **36**(21), 2312053 (2024). 10.1002/adma.202312053 10.1002/adma.202312053 38340045

[155] X. Zhao, T. Jin, W. Gao, G. Niu, J. Zhu et al., Embedding Cs_3_Cu_2_I_5_ scintillators into anodic aluminum oxide matrix for high-resolution X-Ra_*y*_ imaging. Adv. Opt. Mater. **9**(24), 2101194 (2021). 10.1002/adom.202101194

[156] H. Li, H. Yang, R. Yuan, Z. Sun, Y. Yang et al., Ultrahigh spatial resolution, fast decay, and stable X-Ra_*y*_ scintillation screen through assembling CsPbBr_3_ nanocrystals arrays in anodized aluminum oxide. Adv. Opt. Mater. **9**(24), 2101297 (2021). 10.1002/adom.202101297

[157] W.E. van Eijk Carel, Inorganic scintillators in medical imaging. Phys. Med. Biol. **47**(8), R85–R106 (2002). 10.1088/0031-9155/47/8/201 12030568 10.1088/0031-9155/47/8/201

[158] J. Hui, P. Ran, Y. Su, L. Yang, X. Xu et al., High-resolution X-ray imaging based on solution-phase-synthesized Cs_3_Cu_2_I_5_ scintillator nanowire array. J. Phys. Chem. C **126**(30), 12882–12888 (2022). 10.1021/acs.jpcc.2c03453

[159] M. Li, Y. Wang, L. Yang, Z. Chai, Y. Wang et al., Circularly polarized radioluminescence from chiral perovskite scintillators for improved X-ray imaging. Angew. Chem. Int. Ed. **134**(37), e202208440 (2022). 10.1002/ange.202208440 10.1002/anie.202208440 35859274

[160] M. Wang, X. Wang, B. Zhang, F. Li, H. Meng et al., Chiral hybrid manganese(ii) halide clusters with circularly polarized luminescence for X-ray imaging. J. Mater. Chem. C **11**(9), 3206–3212 (2023). 10.1039/D2TC05379A

[161] M.P. Davydova, L. Meng, M.I. Rakhmanova, I.Y. Bagryanskaya, V.S. Sulyaeva et al., Highly emissive chiral Mn(II) bromide hybrids for UV-pumped circularly polarized LEDs and scintillator image applications. Adv. Opt. Mater. **11**(8), 2202811 (2023). 10.1002/adom.202202811

[162] J. Wu, S. You, P. Yu, Q. Guan, Z.-K. Zhu et al., Chirality inducing polar photovoltage in a 2D lead-free double perovskite toward self-powered X-ray detection. ACS Energy Lett. **8**(6), 2809–2816 (2023). 10.1021/acsenergylett.3c00629

[163] S. Cho, S. Kim, J. Kim, Y. Jo, I. Ryu et al., Hybridisation of perovskite nanocrystals with organic molecules for highly efficient liquid scintillators. Light Sci. Appl. **9**, 156 (2020). 10.1038/s41377-020-00391-8 32963768 10.1038/s41377-020-00391-8 PMC7477552

[164] T. Jiang, W. Ma, H. Zhang, Y. Tian, G. Lin et al., Highly efficient and tunable emission of lead-free manganese halides toward white light-emitting diode and X-ray scintillation applications. Adv. Funct. Mater. **31**(14), 2009973 (2021). 10.1002/adfm.202009973

[165] Y. Wei, H. Ebendorff-Heidepriem, J. Zhao, Recent advances in hybrid optical materials: integrating nanoparticles within a glass matrix. Adv. Opt. Mater. **7**(21), 1900702 (2019). 10.1002/adom.201900702

[166] J. Nie, C. Li, S. Zhou, J. Huang, X. Ouyang et al., High photoluminescence quantum yield perovskite/polymer nanocomposites for high contrast X-ray imaging. ACS Appl. Mater. Interfaces **13**(45), 54348–54353 (2021). 10.1021/acsami.1c15613 34735128 10.1021/acsami.1c15613

[167] B. Wang, P. Li, Y. Zhou, Z. Deng, X. Ouyang et al., Cs_3_Cu_2_I_5_ perovskite nanoparticles in polymer matrix as large-area scintillation screen for high-definition X-ray imaging. ACS Appl. Nano Mater. **5**(7), 9792–9798 (2022). 10.1021/acsanm.2c01996

[168] A. Giuri, A.C. Chekkallur, M. Calora, R. Mastria, L. Martinazzoli et al., 3D printed ultra-fast plastic scintillators based on perovskite-photocurable polymer composite. Adv. Funct. Mater. **35**(12), 2417653 (2025). 10.1002/adfm.202417653

[169] X. Wu, Z. Guo, S. Zhu, B. Zhang, S. Guo et al., Ultrathin, transparent, and high density perovskite scintillator film for high resolution X-ray microscopic imaging. Adv. Sci. **9**(17), e2200831 (2022). 10.1002/advs.202200831 10.1002/advs.202200831 PMC9189653 35478488

[170] K. Han, K. Sakhatskyi, J. Jin, Q. Zhang, M.V. Kovalenko et al., Seed-crystal-induced cold sintering toward metal halide transparent ceramic scintillators. Adv. Mater. **34**(17), e2110420 (2022). 10.1002/adma.202110420 35231955 10.1002/adma.202110420

[171] X. Hao, L. Nie, X. Zhu, G. Zeng, C. Liu et al., High-resolution X-ray image from copper-based perovskite hybrid polymer. ACS Appl. Mater. Interfaces **16**(22), 29210–29216 (2024). 10.1021/acsami.4c03401 38770774 10.1021/acsami.4c03401

[172] B. Li, Y. Xu, X. Zhang, K. Han, J. Jin et al., Zero-dimensional luminescent metal halide hybrids enabling bulk transparent medium as large-area X-ray scintillators. Adv. Opt. Mater. **10**(10), 2102793 (2022). 10.1002/adom.202102793

[173] A.J.J.M. van Breemen, M. Simon, O. Tousignant, S. Shanmugam, J.-L. van der Steen et al., Curved digital X-ray detectors. NPJ Flex. Electron. **4**, 22 (2020). 10.1038/s41528-020-00084-7

[174] Y. Zhou, X. Wang, T. He, H. Yang, C. Yang et al., Large-area perovskite-related copper halide film for high-resolution flexible X-ray imaging scintillation screens. ACS Energy Lett. **7**(2), 844–846 (2022). 10.1021/acsenergylett.2c00075

[175] L. Lian, X. Wang, P. Zhang, J. Zhu, X. Zhang et al., Highly luminescent zero-dimensional organic copper halides for X-ray scintillation. J. Phys. Chem. Lett. **12**(29), 6919–6926 (2021). 10.1021/acs.jpclett.1c01946 34282920 10.1021/acs.jpclett.1c01946

[176] W. Li, Y. Li, Y. Wang, Z. Zhou, C. Wang et al., Highly efficient and flexible scintillation screen based on organic Mn(II) halide hybrids toward planar and nonplanar X-ray imaging. Laser Photonics Rev. **18**(2), 2300860 (2024). 10.1002/lpor.202300860

